# Multi-omics approach highlights differences between RLP classes in *Arabidopsis thaliana*

**DOI:** 10.1186/s12864-021-07855-0

**Published:** 2021-07-20

**Authors:** C. E. Steidele, R. Stam

**Affiliations:** Chair of Phytopathology, TUM School of Life Sciences Weihenstephan, Technical University of Munich, Emil-Ramann- Straße 2, 85354 Freising, Germany

## Abstract

**Background:**

The Leucine rich-repeat (LRR) receptor-like protein (RLP) family is a complex gene family with 57 members in *Arabidopsis thaliana*. Some members of the RLP family are known to be involved in basal developmental processes, whereas others are involved in defence responses. However, functional data is currently only available for a small subset of RLPs, leaving the remaining ones classified as RLPs of unknown function.

**Results:**

Using publicly available datasets, we annotated RLPs of unknown function as either likely defence-related or likely fulfilling a more basal function in plants. Then, using these categories, we can identify important characteristics that differ between the RLP subclasses. We found that the two classes differ in abundance on both transcriptome and proteome level, physical clustering in the genome and putative interaction partners. However, the classes do not differ in the genetic di versity of their individual members in accessible pan-genome data.

**Conclusions:**

Our work has several implications for work related to functional studies on RLPs as well as for the understanding of RLP gene family evolution. Using our annotations, we can make suggestions on which RLPs can be identified as potential immune receptors using genetics tools and thereby complement disease studies. The lack of differences in nucleotide diversity between the two RLP subclasses further suggests that non-synonymous diversity of gene sequences alone cannot distinguish defence from developmental genes. By contrast, differences in transcript and protein abundance or clustering at genomic loci might also allow for functional annotations and characterisation in other plant species.

**Supplementary Information:**

The online version contains supplementary material available at 10.1186/s12864-021-07855-0.

## Background

Plants are caught in ever ongoing evolutionary interactions with their pathogens, that have, dependent on their nature, been described as arms races or trench warfare, each with their own underlying evolutionary dynamics [1]. In either case, plants need to evolve resistance mechanisms in order to survive, while pathogens need to simultaneously evolve to overcome these resistances and remain virulent, which in turn necessitates the plant’s defences to evolve again. This leads to the hypothesis that defence-associated genes should be faster evolving than, for example, development-associated genes. On a phylogenetic scale this can be illustrated by very large, diverse and expanded resistance associated gene-families. Most known are the intracellular receptor genes of the NLR family (nucleotide-binding domain and leucine-rich repeat containing receptor family). This family, but also other leucine-rich repeat (LRR)-containing defence-associated genes, drastically diversified over the course of evolution. Indeed, NLRs are much more diverse than for example the defensin gene family which is known to have dual roles in defence as well as development [2]. The enormous variation in NLRs between species and also variation in how these modular receptors are built-up have been discussed in many different papers [3, 4].

How much diversity exists in defence gene families within a species is a less-studied topic however. Recently, polymorphisms and significant copy number variations have been documented within the NLR family in 64 resequenced *Arabidopsis thaliana* accessions [5]. Another study investigating sequence polymorphisms in NLRs from a single tomato species found that NLRs experience different selective pressures dependent on the geographical location of the population [6]. These studies therefore highlight that defence-associated gene families appear to be highly diverse but do not allow comparisons between defence- and development-associated genes in the same gene family.

Besides the NLRs, plants have evolved a plethora of plasma-membrane bound or associated receptors to monitor their environment, but also as a communication tool within the plant itself to facilitate processes like stomatal patterning for example. The different plasma-membrane located receptors can be divided into two major groups, receptor-like kinases (RLKs) with an intracellular signalling domain and receptor-like proteins (RLPs), which only contain a small or absent cytoplasmic tail. Besides the differentiation between RLKs and RLPs, the receptors can be categorized according to their extracellular domains. These domains can facilitate binding and recognition of the corresponding ligands or enable interaction with other proteins to maintain or finetune signalling [7]. In Arabidopsis more than 600 RLKs are annotated [8] and 57 LRR-RLPs, referred to as RLPs in this study, are identified and numbered in consecutive order according to their gene numbers along the Arabidopsis genome [9, 10]. Members of the RLP family have been shown to be involved in both developmental and defence mechanisms, making them ideally suited to investigate whether functional differences lead to differences in rates of evolution.

Of the 57 annotated RLPs in *Arabidopsis thaliana*, 2 RLPs are experimentally validated to be associated with developmental functions (RLP10/CLV2, RLP17/TMM), and 6 with defence functions (RLP1, 3, 23, 30, 32, 42). CLAVATA2(CLV2)/RLP10 seems to be a unique RLP as it plays a role both in developmental and defence-related processes. The best characterised function of CLV2 is in regulation of shoot apical meristem (SAM) maintenance, but it also plays a role in regulation of root apical meristem (RAM) maintenance, regulation of the protoxylem formation, organ development and plant-microbe interactions [11]. Additionally, two other RLPs (RLP2 and 12) can rescue the *clv2*-phenotype when the corresponding genes are expressed under the clv2-promoter [12]. RLP17, also named TOO MANY MOUTH (TMM), is involved in the regulation of the patterning of stomata, micropores to facilitate gas exchange which are located in the epidermis of plant leaves [13, 14].

Fritz-Laylin et al. (2005) used a comparative approach with several criteria including global alignability, genomic organization and sequence identity to identify PUTATIVE DEVELOPMENTAL ORTHOLOGS (PDOs) in Arabidopsis and rice. Based on this classification 4 RLPs could be identified: PDO1/RLP51, PDO2/RLP4, PDO3/RLP10/CLV2, PDO4/RLP17/TMM. Furthermore, they could show that based on phylogenetic comparisons, 47 of 57 AtRLPs group together in so-called superclades. They found that the PDOs do not fall into those superclades, nor do RLP29, 44, 46, 55, 57. Thus, for these RLPs a putative function in development was hypothesized [10]. It was later shown that RLP44 mediates the response to pectin modification by activating brassinosteroid signaling [15] and is important for the regulation of xylem fate [16]. PDO1/RLP51 is the underlying gene of the snc2-1D locus (for suppressor of npr1, constitutive 2-1D), a semidominant gain-of-function *Arabidopsis thaliana* mutant with dwarf morphology and constitutively activated defense responses including high salicylic acid and PATHOGENESIS-RELATED (PR) genes levels [17]. Therefore, we refer to those 9 RLPs (RLP4, 10/CLV2, 17/TMM, 29, 44, 46, 51, 55, 57) as PDOs.

Several RLPs have been shown to fulfill important roles in defence against pathogens. Plants have evolved a two-layered, pathogen-activated immune system to detect and fight off invading pathogens: pattern-triggered immunity (PTI) or surface immunity and effector-triggered immunity (ETI) or intracellular immunity. According to the current and simplified paradigm, pathogen associated molecular patterns (PAMPs) are recognized by cell-surface localized pattern recognition receptors and larger pathogen-secreted proteins, called effectors, are typically recognized by intracellular NLR-receptors [18–21]. There is some debate as to whether the separation of the recognised molecules (PAMPs vs. effectors) can be made that strictly [21, 22]. Several LRR-RLPs have been demonstrated to facilitate immune responses to help protecting the plant against different pathogens.

For example, RLP1/ReMAX (RECEPTOR of eMAX) can detect the ENIGMATIC MAMP OF XANTHOMONAS (eMAX) [23, 24] and RLP23 detects a widespread, but conserved twenty amino acid long epitope in NECROSIS AND ETHYLENE INDUCING (NEP) - LIKE PROTEINS (NLPs) [25]. This so-called nlp20 motif is present in NLPs from fungi, oomycetes and bacteria [26]. The currently unidentified SCLEROTINIA CULTURE FILTRATE ELICITOR 1 (SCFE1) is perceived *via* RLP30 [27]. RLP32 recognizes the structural fold of the bacterial translation initiation factor − 1 (Inf-1) present in all proteobacteria [31] and RLP42/RBPG1 detects several endopolygalacturonases from *Botrytis cinerea* and *Aspergillus niger* [28]. Finally, RLP3 is the causal gene of the quantitative resistance locus RFO2 in Arabidopsis conferring resistance against the vascular wilt fungus *Fusarium oxysporum* forma specialis *matthioli* [29]. As these 6 RLPs (RLP1, 3, 23, 30, 32 and 42) play important roles in the defence against various pathogens we will refer to them as VDRs (validated defence RLPs) in the remainder of this manuscript.

RLPs lack an obvious intracellular signalling domain and thus require additional interaction partners. For the VDR RLP23 it was shown that the short cytoplasmic tail has, if at all, only an auxillary but not essential function in nlp20-mediated ethylene signalling [30]. The VDRs RLP1, RLP23, RLP30, RLP32 and RLP42 all require BRASSINOSTEROID-INSENSITIVE KINASE 1 (BAK1) and SUPPRESSOR OF BIR1 (SOBIR1) for full function. The aforementioned RLPs are constitutively associated with SOBIR1 at the plant plasma membrane, then upon ligand perception BAK1 is recruited to the complex [23, 25, 27, 28, 31]. The interaction with SOBIR1 is mediated via a stretch of negatively charged amino acids, Aspartate (D) and Glutamate (E), in the extracellular juxtamembrane region, just before the transmembrane domain and a conserved GxxxG motif within the transmembrane region [30].

The PDO RLP10/CLV2 interacts with the kinase CORYNE (CRN) and together they can form a multimer with the LRR-kinase CLAVATA 1(CLV1) [32]. RLP17/TMM forms a receptor complex with the ERECTA RECEPTOR KINASES (ER) or ER-LIKE 1 (ERL1) to regulate stomatal patterning [33]. Though these analyses are far from complete, they seem to suggest distinct evolutionary trajectories for PDOs and VDRs.

Over the last decades, a large number of publicly accessible datasets have become available for *A. thaliana* research. These data sets range from (reference) genome data (The Arabidopsis Information Resource, TAIR, [34]) and gene expression atlasses [35] to the 1001 Arabidopsis genome project [36]. Very recently, a full *A. thaliana* transcriptome and proteome database was published [37] as well as a copy number variant atlas, cataloging presence and absence variation between over 1100 *A. thaliana* accessions [38]. The availability of these data sets for the first time allows comparisons of gene diversity and gene families on many levels.

In this paper, we utilize the publicly available *A. thaliana* reference genome, the gene expression atlas, an Arabidopsis transcriptome and proteome database, the sequencing data from the 1001 Arabidopsis genome project as well as a copy number variant atlas to gain a deeper understanding of the function and putative role of the RLP family in Arabidopsis. Knowing that the RLP family contains both developmental and defence-associated members, we specifically focus on comparing those two classes. We investigate the two subfamilies on different levels, ranging from phylogenetic relationship, gene expression in induced and native states, proteome analyses, single-nucleotide polymorphisms to presence-absence variation. Our results show distinguishable characteristics between defence and development associated RLPs.

## Results

### Defence- and development-associated RLPs cluster differently in the phylogenetic tree

**Fig. 1:**
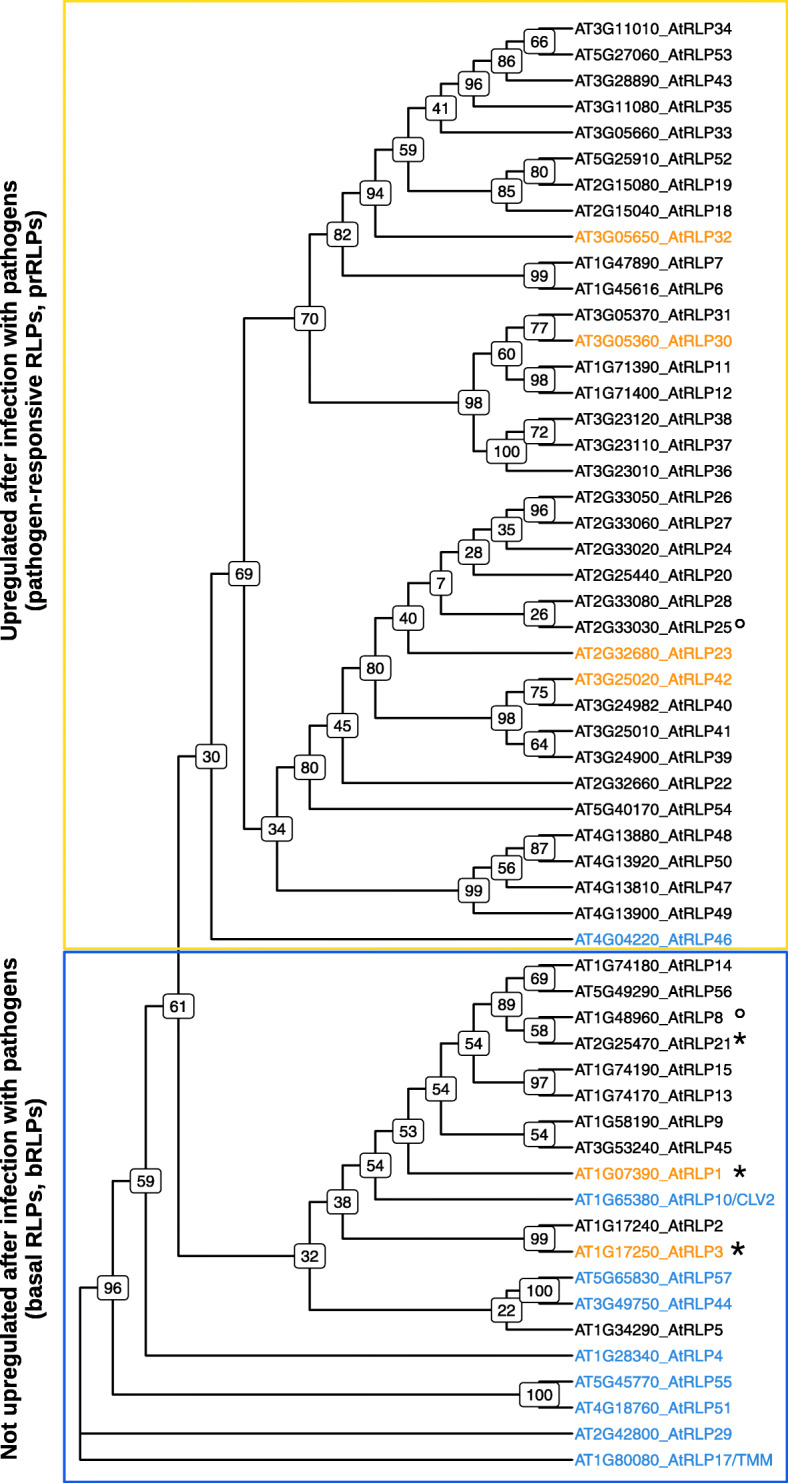
Phylogenetic tree of the conserved C3-F domain of RLPs. The tree was generated using RaxML with 1000 bootstraps. The basal RLPs (bRLPs) which are not upregulated after pathogen treatment and the pathogen-responsive RLPs (prRLPs) which are upregulated after pathogen treatment cluster together within the phylogenetic tree.Highlighted in blue are the putative developmental orthologs (PDOs) and in yellow the validated defence RLPs (VDRs). Boxed in yellow are the pathogen-responsive RLPs (prRLPs) that are at least 2.5x upregulated with a p-value of 0.001 after infection with various pathogens (except AtRLP6, 47 and 48 which are only 1.5x upregulated). Boxed in blue are the basal RLPs (bRLPs) which were not upregulated by pathogen infection (<|2.5|, *p*-value = 0.001). Used datasets are AT_AFFY_ATH1-0 and AT_mRNAseq_ARABI_GL-3. *AtRLP1, AtRLP3 and AtRLP21 showed an upregulation after pathogen treatment. °AtRLP25 is not up or down regulated at all and AtRLP8 was not present in the used datasets

First we wanted to know whether we could split the RLP family in a defence-associated and a development-associated fraction. The most straightforward way to infer RLP functions would be if genes with similar functions e.g. defence or conserved roles, would share higher sequence similarity and thus cluster together in phylogenetic trees. Four papers studied the phylogeny of RLPs previously [9, 10, 39, 40]. All of them used up to 100 bootstraps and at the time of publication, not many RLPs were functionally annotated. We redid the phylogenetic analysis as previously performed by Wang et al. [9] who used the conserved C3-F domain, with 1000 bootstraps using RaxML [41]. Our tree resembles the phylogeny by Wang et al., [9] with high support values for most internal branches (Fig. 1), confirming the validity of this tree. We used the tree and annotated the aforementioned PDOs (RLP4, 10/CLV2, 17/TMM, 29, 44, 46, 51, 55, 57) and VDRs (RLP1, 3, 23, 30, 32 and 42). The PDOs, except RLP46, are all on the basal branches of the phylogenetic tree, whereas the defence-associated RLPs are more scattered across the tree and also populate the larger non-basal part. This is in line with previous publications where already a higher number of RLPs was predicted to be associated with defence and where it was further shown that 47 out of the analyzed 57 RLPs cluster within superclades where at least one member was defence-associated [10].

### Phylogenetic clustering of RLPs correlates mostly with changes to protein expression levels after infection with pathogens

Based on the findings above, we hypothesized that RLPs on the upper branches of the tree are more likely to beassociated with defence. To expand the annotation data of the RLPs, we used the Genevestigator software [35]. The expression of those RLPs after pathogen treatment was checked in two different datasets containing expression data for treatment of *A. thaliana* with several bacterial and filamentous pathogens. Thirty-five RLPs showed upregulated gene expression patterns after treatment with pathogens in at least one of the different pathogen infection datasets, whereas 17 RLPs showed no changes in expression after pathogen treatment in any of the examined data sets. We found that all previously identified defence-associated RLPs are upregulated, whereas only one of the previously annotated PDOs show changes after infection (RLP46). Interestingly, when we superimpose the expression data on the phylogeny we see a clear, but not perfect separation of upregulated RLPs in all higher branches (Fig. 1, yellow box) yet almost no difference in the expression levels after pathogen infection of the RLPs clustering in the lower, basal branches (Fig. 1, blue box). The only four exceptions are the two VDRsd, RLP1 and RLP3, as well as RLP21 which are all basal in the tree, but show a gene upregulation after infections. In contrast, RLP46, which was previously annotated as developmental [10] and thus grouped as a PDO, in our examination clearly shows upregulation after pathogen treatments and is thus rightly assigned to the upper cluster. RLP25 shows no changes in expression in any of the examined datasets and RLP8 was missing from the data.

When combined, these data suggest that the upper part of the phylogenetic tree most likely contains defence-associated RLPs that are all derived from more ancestral, putative developmental-related RLPs. In the remainder of this manuscript we will therefore refer to the upper part of the phylogeny as prRLPs (pathogen-responsive RLPs) and the lower part as bRLPs (basal RLPs).

### Pathogen-responsive RLPs are species specific in Arabidopsis and tomato

After grouping the Arabidopsis RLPs into two classes with a hypothetical association to defence-responses (prRLPs) and a more conserved, putative development-related function (bRLPs), we wondered whether this division can also be seen outside the species. Kang and Yeom [42] recently published a completely updated annotation of all RLPs in tomato (*Solanum lycopersicum).* Similar to Wang et al. [9] and our phylogeney presented above, they generated a phylogenetic tree from the C3-F domain, using all available RLPs for both tomato and Arabidopsis. Interestingly, the bRLPs can be found both as poly- and paraphyletic groups with the annotated tomato RLPs. The Arabidopsis prRLPs all form a single monophyletic group (Figure S1), thus indicating that the Arabidopsis prRLPs derive from species-specific family expansions.

### Basal RLPs are more likely to lack common protein-interacting motifs

Known defence-associated RLPs constitutively interact with SOBIR1 [25, 43] and for this interaction two motifs are important: a stretch of negatively charged amino acids in the extracellular juxtamembrane region and a conserved GxxxG-motif in the transmembrane domain [30, 44].

**Fig. 2:**
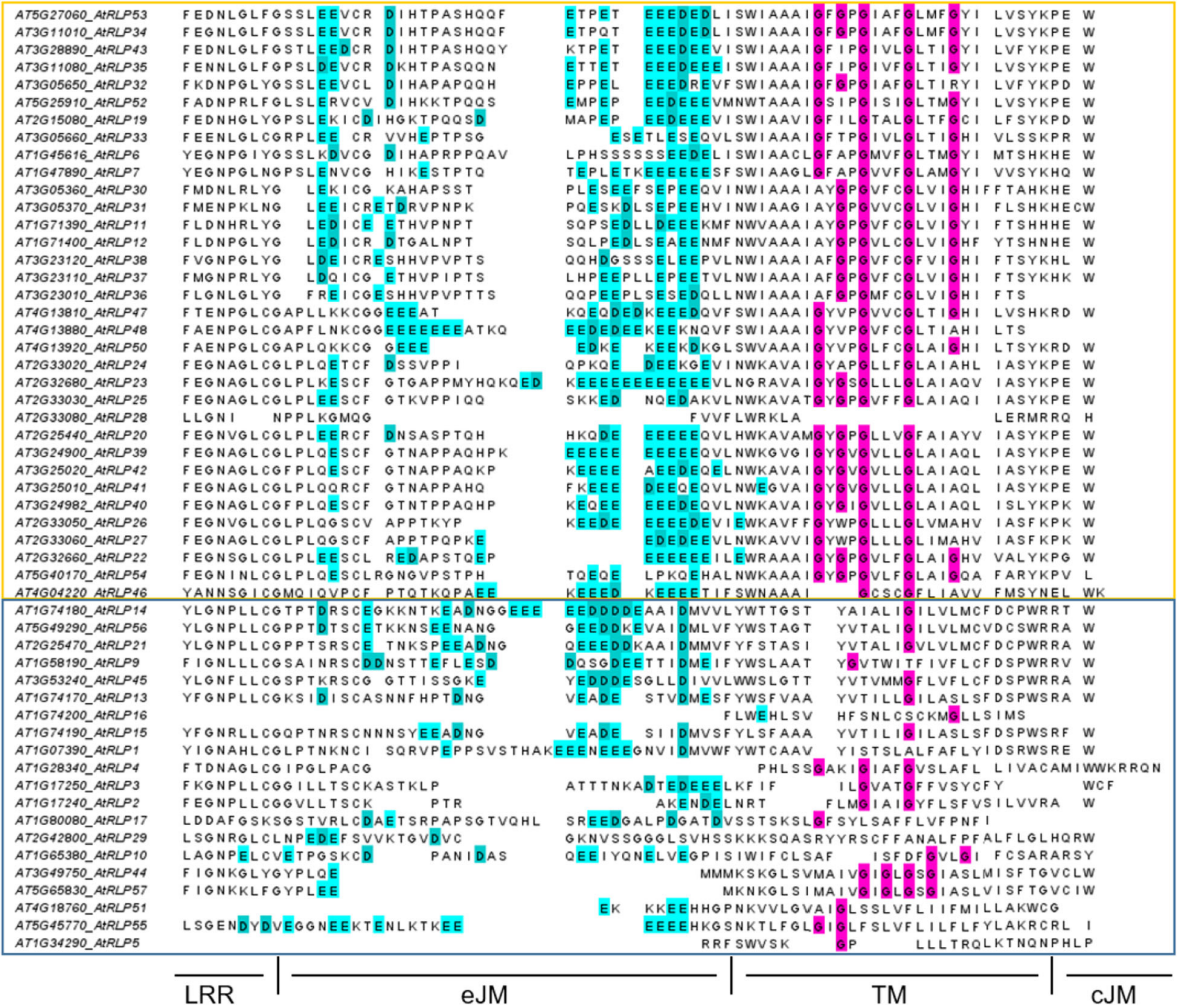
Alignment of the extracellular juxtamembrane, transmembrane and cytoplasmic region of the RLPs. Most of the prRLPs (boxed in yellow) have both motifs required for interaction with SOBIR1, the negatively charged amino acid stretch in the eJM and the GxxxG-motif in the TM, except RLP28 lacking both motifs and RLP46 having only two Gs. Most of the bRLPs (boxed in blue) lack the GxxxG-motif, except RLP44 and RLP57, but the latter lack a dominant negatively-charged amino acid stretch in the eJM. All sequences were aligned using MUSCLE and the sequences were ordered manually to fit the phylogenetic tree. The last Leucine rich repeat-domain (LRR), as well as the extracellular juxtamembrane (eJM), the transmembrane domain (TM) and the cellular juxtamembrane (cJM) are indicated. Color coded in magenta are the Glycines (G) in the TM and in cyan the Aspartates (D) and Glutamates (E) in the eJM

All of the VDRs possess the conserved stretch of negatively charged amino acids (Aspartate (D) and Glutamate (E)). Only RLP1 lacks the GxxxG-motif, but it was shown that it can still interact with SOBIR1 [30]. From the bRLPs only RLP55 has a pronounced stretch of Aspartate and Glutamate. RLP17/TMM and RLP29 contain neither the negatively charged amino acids nor the GxxxG-motif. We expanded these analyses and investigated the presence of these motifs in the complete prRLP set and the non-pathogen responsive bRLP set and found that with only one exception all pathogen-responsive RLPs contain both motifs, whereas one or in some cases even both motifs appear to be absent in the bRLP set (Fig. 2). This suggests that SOBIR1-dependency evolved in relation to a function in pathogen defence.

### Basal and pathogen-responsive RLPs differ in their transcriptomic profiles

Based on our findings so far, we wanted to know if besides phylogenetic separation the two groups, prRLPs and bRLPs, show other globally different characteristics. For example, one can hypothesize that defence-associated and non-defence-associated RLPs also show different transcript levels in native states.

**Fig. 3:**
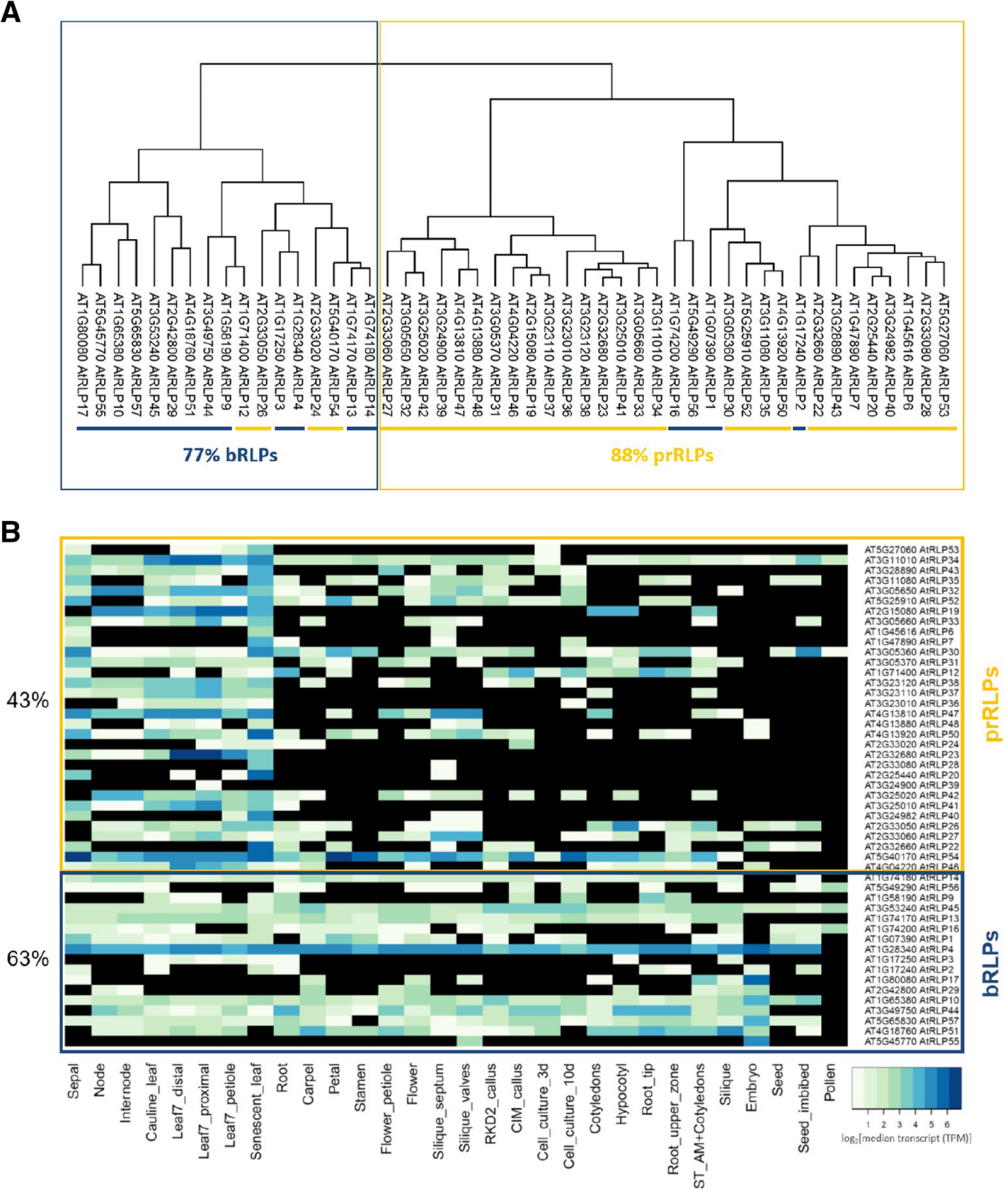
Transcriptomic clustering. (**A**) The dendrograms represent the hierarchical clustering of the transcripts of RLP-genes in various tissues after imputation of missing values [37]. (**B**) The heatmap shows the absence/presence of RLP transcripts in various uninduced tissues in Arabidopsis [37]. Transcript abundance is indicated by the coloring code as log_2_ of median transcript (TPM, transcript per million). Black means no transcript was found. The presence of each gene transcript over all tested tissues was calculated and the average for each set (prRLPs and bRLPs) is shown in percentage. Boxed in yellow are prRLPs and in blue bRLPs

Therefore, we examined the steady state expression levels of all RLPs in different tissues. We obtained such expression data, consisting of different tissue samples from the Arabidopsis proteome project [37] and looked for similar expression patterns using a hierarchical clustering method. Figure 3 A shows a clustering into a predominantly pathogen-responsive cluster (88 % of the genes are prRLPs) and one cluster with mainly bRLPs (77 % of the genes are bRLPs). It should be noted that no information was available for RLP5, RLP8, RLP11, RLP15, RLP18, RLP21, RLP25 and RLP49.

For many RLPs expression data was only available for very few of the analyzed tissues. In Fig. 3B, we therefore show a heatmap depicting the gene expression levels. Closer inspection shows that the fraction of RLPs with a detectable transcript differs significantly between prRLPs and bRLPs, with a lower fraction detected in the prRLPs: 43 % vs. 63 % (Student’s t-test, p = 0.036). Thus, basal gene expression levels between RLP classes differ.

### Basal and pathogen-responsive RLPs differ in their proteomic profiles

**Fig. 4:**
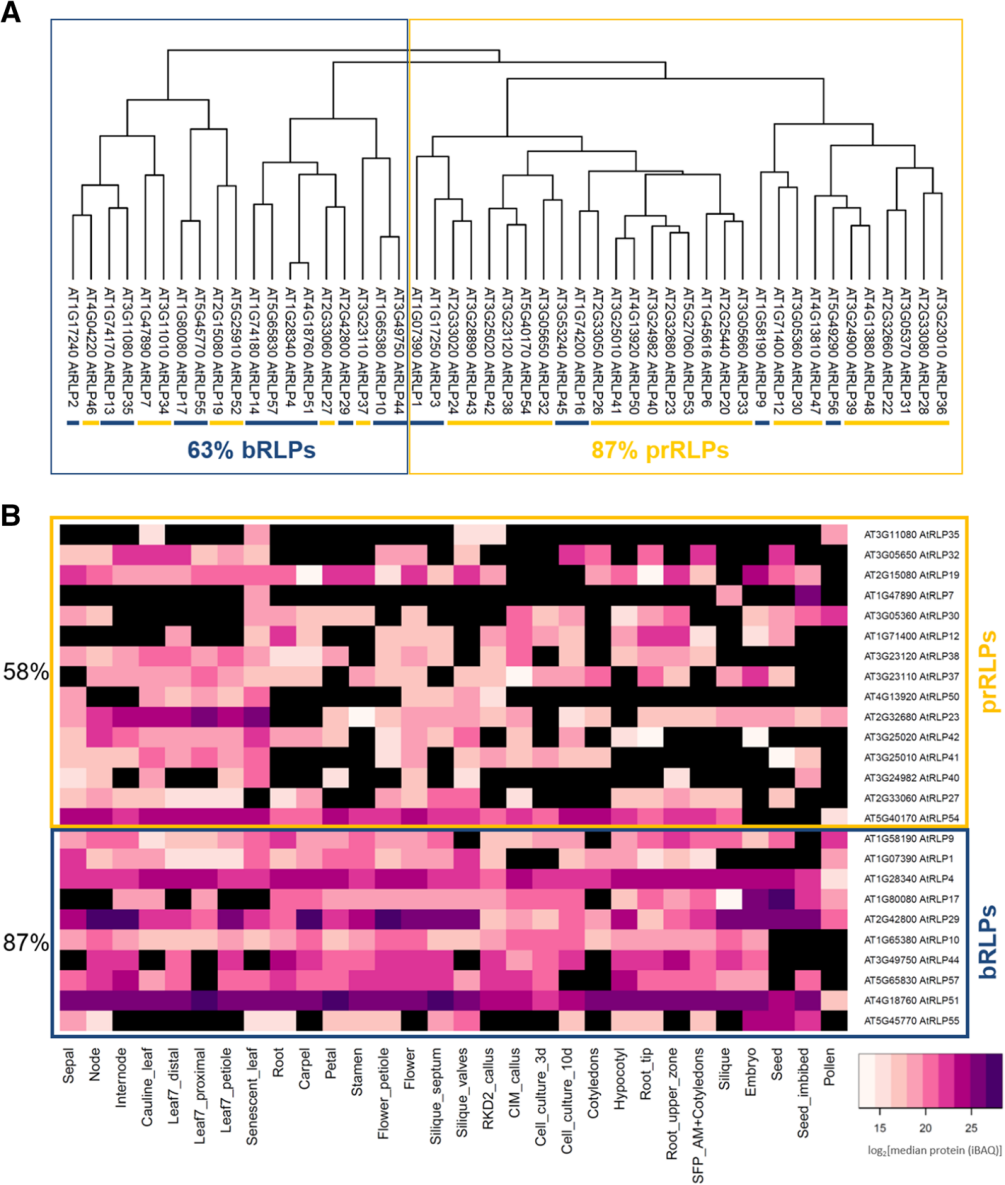
Proteomic clustering. (**A**) The dendrograms represent the hierarchical clustering of the translated RLP-proteins in various tissues after imputation of missing values [37]. (**B**) The heatmap shows the absence/presence of RLP protein in various uninduced tissues in Arabidopsis [37]. Protein abundance is indicated by coloring code as log_2_ of median protein quantities (Intensity Based Absolute Quantification, iBAQ). Black means no protein was found. The presence of each protein over all tested tissues was calculated and the average for each set (prRLPs and bRLPs) is shown in percentage. Boxed in yellow are prRLPs and in blue bRLPs

Knowing that there seem to be tissue-specific differences in expression between the two RLP classes, we further investigated whether these differences can also be observed at the proteome level. When calculating hierarchical clustering on protein abundance, we also observed a clustering of the pr- and bRLPs, although it is less obvious than on the expression level (Fig. 4 A).

Similar to the transcriptome data, the proteome data show significant differences between the fraction of RLPs present in the pathogen-responsive fraction (prRLPs 58 %) versus the basal fraction (bRLPs 87 %) (Student’s t-test, *p* = 0,0005) (Fig. 4B).

### Pathogen-responsive RLPs are more likely to be encoded in a gene cluster

**Fig. 5:**
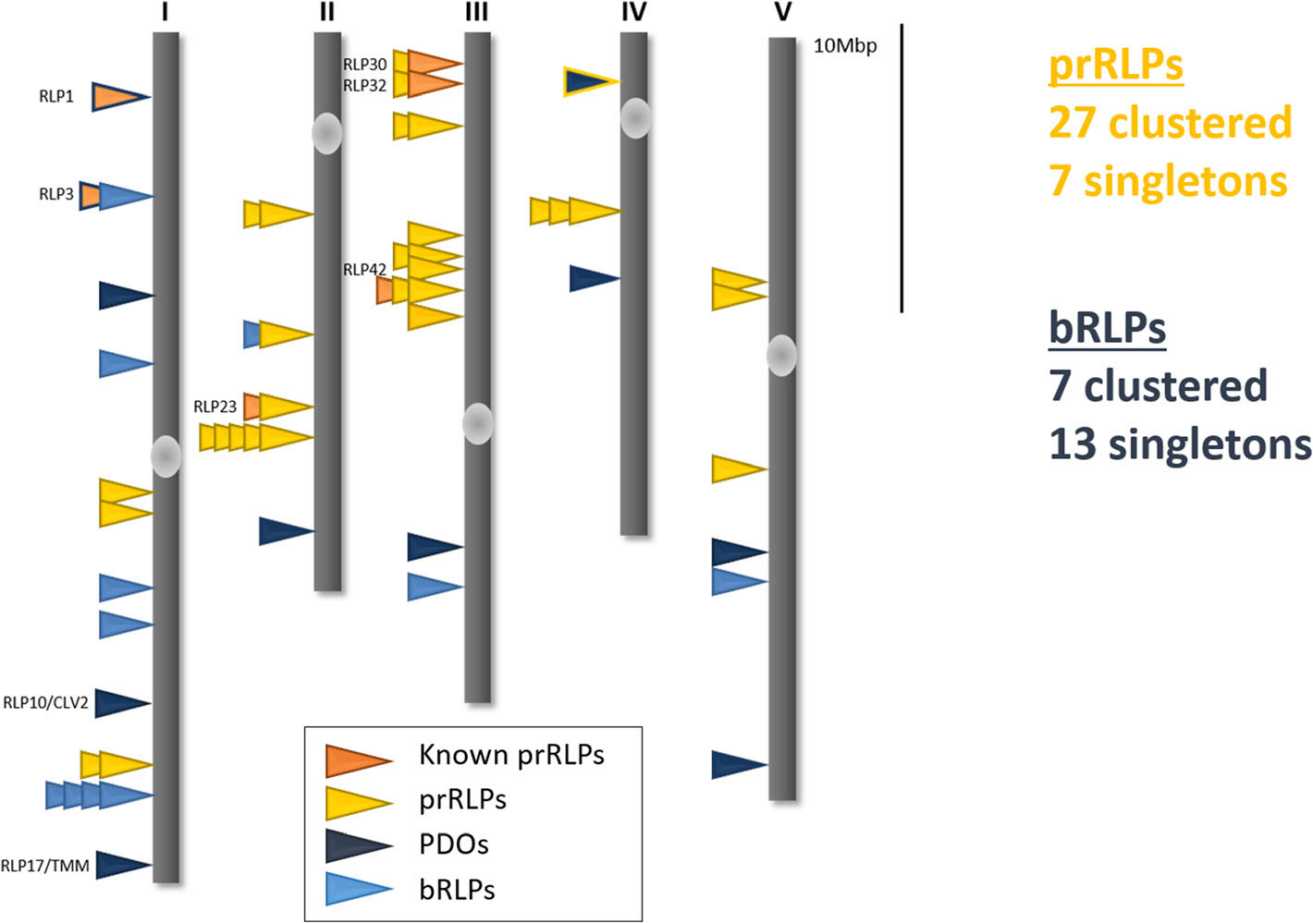
Genomic localization of RLPs. The genomic distribution is schematically depicted. 27 prRLPs are clustered and 7 are encoded as single genes, whereas 7 bRLPs are encoded in clusters and 13 as singletons (*χ*^2^-test, p = 0.003). VDRs are marked in orange, prRLPs in yellow, PDOs in dark blue and bRLPs in light blue. Figure is adapted from [39]

It has been hypothesized that the physical location of defence-associated genes, like those in the NLRs and RLP families, allows for more rapid evolution and recombination and that as such, these gene families evolved in clusters on the genome. Indeed, genes in both families are often co-occurring and clustered on the genome [45], yet singleton RLPs have also been reported. In order to test whether prRLPs more often occur in clusters and other RLPs more often as singletons, we reassessed available genome annotation data and defined RLP clusters (Fig. 5: Figure S2). This show that the fraction of clustered RLPs is higher in the pathogen-responsive RLPs (27 clustered, 7 singletons) than in the basal (7 clustered, 13 singletons) ( *χ*^2^ test, *p* = 0.003), confirming our hypothesis.

### CNVs affect both classes of RLPs similarly

Gene clustering is expected to allow easier generation of copy number variation (CNV) on affected chromosomal loci. In order to test whether defence RLPs differ significantly in CNV compared to other RLPs, we downloaded the CNV database generated by Zmienko et al. [38], who defined CNVs as full as well as partial duplication of a gene or gene fragment. Interestingly, while CNVs are particularly widespread for NLRs [5], just over half of the RLPs [32] showed one or more CNV events. RLPs that occur in clusters are significantly more often affected by CNV events with 71 % of clustered RLPs showing CNVs against only 40 % of singleton RLPs (*χ*^2^-test, *p* = 0.05). Clustering therefore seems to affect the potential for CNV in these genes. There is a tendency that prRLPs more often show CNV events (*χ*^2^-test, *p* = 0.14).

### RLPs show a broad range of single nucleotide polymorphisms (SNPs)

With the knowledge that pathogen-responsive RLPs are often found in clusters, and that this clustering might lead to an observed increase in CNV, we wanted to test whether prRLPs are in general showing higher numbers of polymorphisms or signatures for positive or balancing selection. Analyzing the sequencing data from 1135 Arabidopsis accessions revealed that 22 out of 57 RLPs have no SNPs in coding regions. 41 % (14/34) of the prRLPs and 33 % (7/21) of the bRLPS have no such SNPs. Thus, the fractions are not significantly different (*χ*^2^-test, *p* = 0.87). Looking specifically in the clustered and non-clustered RLPs revealed no difference in the absence or presence of SNPs between RLPs which are encoded as a single gene or those in pairs or larger clusters (40 % [8/20] vs. 39 % [14/34]).

**Fig. 6:**
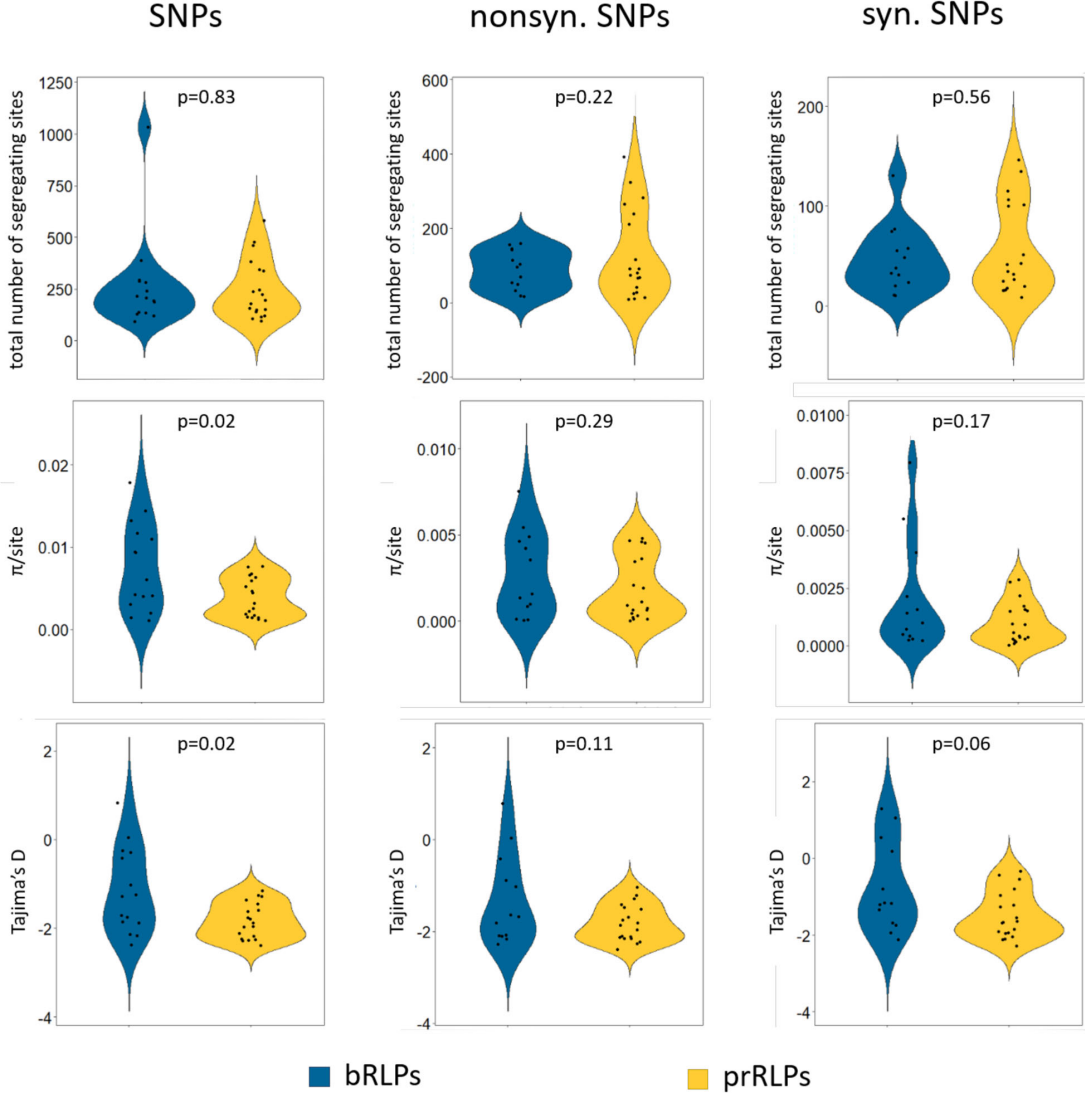
Single nucleotide polymorphisms (SNPs). We used the available sequence information of more than 1000 Arabidopsis thaliana accessions from the 1001 Genome project [36] and calculated the total number of segregating sites, π/site and Tajima’s D value for all SNPs, as well as nonsynonymous and synonymous SNPs using the Popgenome package [52]. *P*-values are calculated using Student’s t-test

Seeing that there are no significant differences between the total number of segregating sites, we looked whether other parameters are different between the two RLP classes and split the analyses into synonymous (e.g. not causing an amino acid change) and non-synonymous (causing an amino acid change) SNPs. To our surprise, the nucleotide diversity measured as π/site is significantly larger in the bRLPs (Fig. 6). Tajima’s D, which can be used as a proxy to estimate evolutionary pressure on the genes, is generally low in all RLPs. This is an indication of an abundance of rare alleles (singleton SNPs) being present and therefore suggests purifying selection or a recent bottleneck. Low Tajima’s D values have been reported for the majority of genes in *A. thaliana* [36]. Interestingly, whereas higher Tajima’s D would be expected for defence-associated genes under diversifying or balancing selection, there are only two RLPs with Tajima’s D values above 0, both of them belong to the bRLPs (RLP2, 0.822; RLP15 0.05). Lastly, we found no significant differences in the ratio of non-synonymous over synonymous polymorphisms between the two classes (Figure S3). Thus, it appears that on the level of DNA polymorphisms, prRLPs and bRLPs cannot be differentiated.

## Discussion

RLPs form a diverse gene family that has been associated with both developmental and defence-associated processes. In this paper, we have combined publicly available data sets in order to make a classification of RLPs and predict putative roles in either defence or more basal, most likely development-associated processes.

The phylogenetic tree based on the C-terminal conserved domains C3 to F together with gene expression data collected by the Genevestigator database showed a clear, although not perfect separation into RLPs upregulated (prRLPs) and not regulated (bRLPs) after various pathogen infections.

This separation is further confirmed by analysis performed by Fritz-Laylin et al. [10]. In their analysis, the RLP sequences of *Arabidopsis thaliana* and rice were compared and based on different criteria, such as homology and genomic localization, a set of nine putative developmental orthologues (PDOs) was defined. This set includes the well-studied CLV2/RLP10 and TMM/RLP17 proteins. Additionally, RLP44 mediates the response to pectin modification by activating brassinosteroid signaling [15] and is important for the regulation of xylem fate [16]. These 9 PDOs are all, except RLP46, not upregulated after pathogen treatment and cluster within the bRLPs.

Interestingly, our defined bRLPs are more closely related to tomato RLPs than to the Arabidopsis prRLPs [42], which form a unique clade, indicating that each species might have a unique set of receptors to fight off invading pathogens yet share commonalities in their basal processes. Similar observations were made by Steinbrenner [46] who suggested that pathogen defences evolved in a lineage-specific manner.

RLPs lack an intracellular signalling domain and therefore need interaction partners for downstream signalling. The confirmed defence-associated RLPs constitutively interact with the adaptor-kinase SOBIR1 and recruit BAK1 in a ligand-dependent manner [25, 30]. The signalling of those RLPs is SOBIR1-dependent, whereas the known PDOs (CLV2/RLP10, TMM/RLP17, RLP44) function independently from SOBIR1 but can be pulled down in overexpression experiments together with SOBIR1 [47]. Two protein-interacting motifs are required for RLP-SOBIR1 interaction, which is a negatively-charged stretch of amino-acids in the extracellular juxtamembrane region and a GxxxG-motif in the transmembrane domain [30]. Alignment of these regions showed that in most of the prRLPs both of the motifs are present, whereas in the bRLPs they are less common or completely absent, suggesting that interaction with SOBIR1 is defence-specific.

We analysed the transcriptomic and proteomic expression profiles of the RLPs in 30 different samples representing different tissues and different development stages [37] and compared the prRLPs with the bRLPs. Both the transcriptome and the proteome showed differences between bRLPs and prRLPs, revealing that the bRLPs are more ubiquitously expressed and transcribed compared to the prRLPs.

Over the past years a number of studies have been published that aimed to identify receptors involved in early pathogen defence responses, so called pattern recognition receptors (PRRs) (for example [25, 27]). Our data showed that all known RLPs reported to function as PRRs show similar patterns in transcriptome and proteome data, especially regarding the presence of the respective protein in an uninduced state. Constitutive presence of a cell surface receptor hence appears as a hallmark of PRRs and is a prerequisite to measure early responses to potential immunogenic elicitors from pathogens. Based on these combined data we can thus predict further RLPs that may act as PRRs and can potentially be identified using early immune response assays. Expression data show upregulation of certain RLP-genes upon pathogen stimulus, indicating that those respective pathogens might harbour the immunogenic motif recognized by the respective PRR. This is true for already identified PRRs like RLP23, which recognizes nlp20, a 20 amino acid long peptide present in NECROSIS AND ETHYLENE PRODUCING (NEP)-LIKE PROTEINS (NLPs) [26]. NLPs are widespread among bacteria, fungi and oomycetes and the expression data on Genevestigator shows that RLP23 is highly upregulated after treatment with pathogens harboring an NLP.

Our predictions can be found in table S1. All of the predicted RLPs belong to the prRLPs and the protein is present in an uninduced state. Each of these, except RLP54, are encoded in a cluster of at least two genes, attributes that we have assigned to be typical for PRRs.

The gene expression atlas on Genevestigator [35] further revealed that some RLPs (RLP4, 19, 21, 26/27, 32, 49/50, 53/34, 54) are likely to be targeted by bacterial effectors as they showed downregulation of gene expression after treatment with a wild-type bacterial strain and a strong upregulation after infection with bacterial strains deficient in effector secretion. Additionally, no RLP was upregulated after wounding, maybe indicating that RLPs are not able to sense damage-associated molecular patterns (DAMPs).

Besides the previously mentioned observation that the prRLPs seem to have evolved species specifically in *A. thaliana*, the bRLPs and prRLPs show a number of interesting genomic differences that illustrate possible differences in their evolutionary trajectory. We do not observe clear differences between the classes in terms of nucleotide diversity. This might be because some of the bRLPs have dual roles (like CLV2) [11], or because the cluster of bRLPs also contains some defence-associated RLPs. Nucleotide diversity and Tajima’s D differ between bRLPs and prRLPs but this difference seems to be mainly driven by two highly diverse bRLPs.

It might not come as a surprise that RLPs do not generally exhibit high diversity, as illustrated by the lack of SNPs in some RLPs and generally low Tajima’s D values, because developmental processes are assumed to be conserved and VDRs in *A. thaliana* are detecting conserved PAMPs. Yet the stark differences in the amount of polymorphisms in some prRLPs as well as some bRLPs might indicate specific roles for these more diverse RLPs.

We do find that prRLPs are significantly more often encoded in gene clusters than bRLPs and that prRLPs are more often affected by CVNs. Recently, such intragenic recombinations have been shown to play a major role in the maintenance of stable polymorphisms in an important NLR resistance gene against the pathogen *P. infestans* in the wild potato species *Solanum americanum* [48], as well as in the RLP locus Hcr9, conferring resistance against the fungal pathogen *Cladosporium fulvum* in wild tomato [49].

Overall, by combining several public resources, we enhance current knowledge of the RLP gene family in Arabidopsis. We were able to group the RLPs into two hypothetical classes, a pathogen-responsive sub-family and a basal sub-family, each with their own characteristics. Further research on the identification of the role of RLPs in various cellular processes will help to better understand the observed differences within the RLPs and prove our suggested hypothetical grouping into prRLPs and bRLPs. For now, this distinguishment could provide an interesting starting point for comparative studies in other plant species and might help researchers working on the biology of RLPs.

## Methods

### Phylogenetic analyses

The phylogenetic tree of the Arabidopsis’ RLPs was made using the conserved C3-F domain, as previously reported by Wang et al. [9]. The phylogeny was made using RaxML-HPC [41] with the GTR model and thousand bootstraps (raxmlHPC -f a -m PROTCATGTR -# 1000). The b- and prRLPs were assigned based on the gene expression data available on Genevestigator [35]. We checked for genes which were at least 2.5x upregulated with a p-value of 0.001 after infection with various pathogens. The used datasets were AT_AFFY_ATH1−0 and AT_mRNAseq_ARABI_GL−3.

The phylogenetic tree of the RLPs from tomato and Arabidopsis was made by Kang and Yeom [42] using the amino acid sequences of the C3-F domains using PhyMl.

### Domain alignment

The full-length protein sequence of all RLPs was aligned using MUSCLE [50] using the default settings and the sequences were afterwards ordered manually to fit the phylogenetic tree and trimmed to the last LRR-domain, the extracellular juxtamembrane region, the transmembrane domain and the intracellular juxtamembrane region as it was done by [44]. The resulting multiple sequence alignments can be found in the supplementary materials.

### Transcriptome and proteome data clustering

Both transcriptome and proteome data for 30 *Arabidopsis thaliana* tissues were obtained from the Arabidopsis Proteome project (https://www.proteomicsdb.org/). Detailed experimental procedures on data generation and normalization can be found in the accompanying paper [37]. In that paper, transcriptome and proteome data were obtained from different *Arabidopsis* tissues. The transcriptome and proteome data were log normalised over the median and merged into two tables. Missing data were imputed around the mean using random numbers drawn from the lower part of the normal distribution with the standard settings for width (0.3) and downward shift (1.8) in the Perseus software (v1.6.5.0) [51] Width: Defines the width of the Gaussian distribution relative to the standard deviation of measured values. Down shift: Specifies the amount by which the distribution used for the random numbers is shifted downwards as described on: http://coxdocs.org/doku.php?id=perseus:user:activities:MatrixProcessing.

### Imputation:ReplaceMissingFromGaussian

For each of the two data sets (normalized and imputed transcriptomic and proteomic data) we calculated the Pearson correlations between the normalised TPM values for the transcriptome and normalised iBAQ values for the proteome in R, using cor, followed by clustering using hclust (method = complete for proteome and ward.d2 for transcriptome) and plotting as.dendogram. The normalized data and scripts for clustering can be found in the supplementary materials.

### Genomic clustering and CNV analysis

The genomic clustering is based on the analysis done by Tör et al. [39] and the start sites of each gene were taken from their annotation. To test whether the observed number of bRLPs and prRLPs in clusters differed from expected values, we used the *χ*^2^-test (chisq.test) as implemented in the R stats package.

For CNV analyses, we used the CNV definition and the dataset as described in [38]. The genomic coordinates of the CNVs were extracted from the supplementary data and converted to bed format. Next, we used bedtools intersect -wo to find overlapping regions. CNVs were counted for bRLPs and prRLPs and their distribution was compared with expected ratios using the *χ*^2^-test in R as described before. All above mentioned RLP annotations and scripts for the calculations can be found in the supplementary information online.

### Genetic diversity analyses of 1001 genome data

The sequencing data for 1135 Arabidopsis accessions was downloaded from the 1001 genomes project homepage (https://1001genomes.org/) and the Col-0 reference genome was downloaded from (https://plants.ensembl.org/).

The nucleotide diversity statistics were calculated with the R package PopGenome [52] using the functions diversity.stats and neutrality.stats. All statistics were calculated for all sites and separately for the synonymous and nonsynonymous sites using the subsites function. To obtain comparable π/site values, the π-value was divided by the gene length. Scripts can be found in the supplementary information online.

## Supplementary Information

**Additional file 1:**

**Additional file 2:**

**Additional file 3:**

**Additional file 4:**

**Additional file 5:**

## Data Availability

Intermediate data files, such as multiple sequence alignments, mentioned in the manuscript as well as the R script used for the analysis of the transcriptome, proteome, 1001 genome diversity as well as those used to calculate the correlations between the expected classification and specific features (clustering, upregulation, etc. ) are uploaded as supplementary materials.

## Abbreviations

bRLP: basal RLP
CNV: Copy Number Variants
LRR: Leucine Rich Repeat
NLR: Nucleotide-binding domain and Leucine-rich repeat containing Receptor family
PAMP: Pathogen Associated Molecular Pattern
PDO: Putative Developmental Orthologues
PRR: Pattern Recognition Receptor
prRLP: pathogen regulated RLP
RLP: Receptor Like Protein
RLK: Receptor-Like Kinase
TMP: Transcripts per million
VDR: Validated Defence RLPs
