# Supplementary material for: Multi-omics approach highlights differences between RLP classes in Arabidopsis thaliana
Source: BMC Genomics. 2021 Jul 20;22:557. doi: 10.1186/s12864-021-07855-0 (PMC8290556; doi:10.1186/s12864-021-07855-0)
Supplement: Supplementary file 1 — Additional file 1: [file 12864_2021_7855_MOESM1_ESM.pdf]

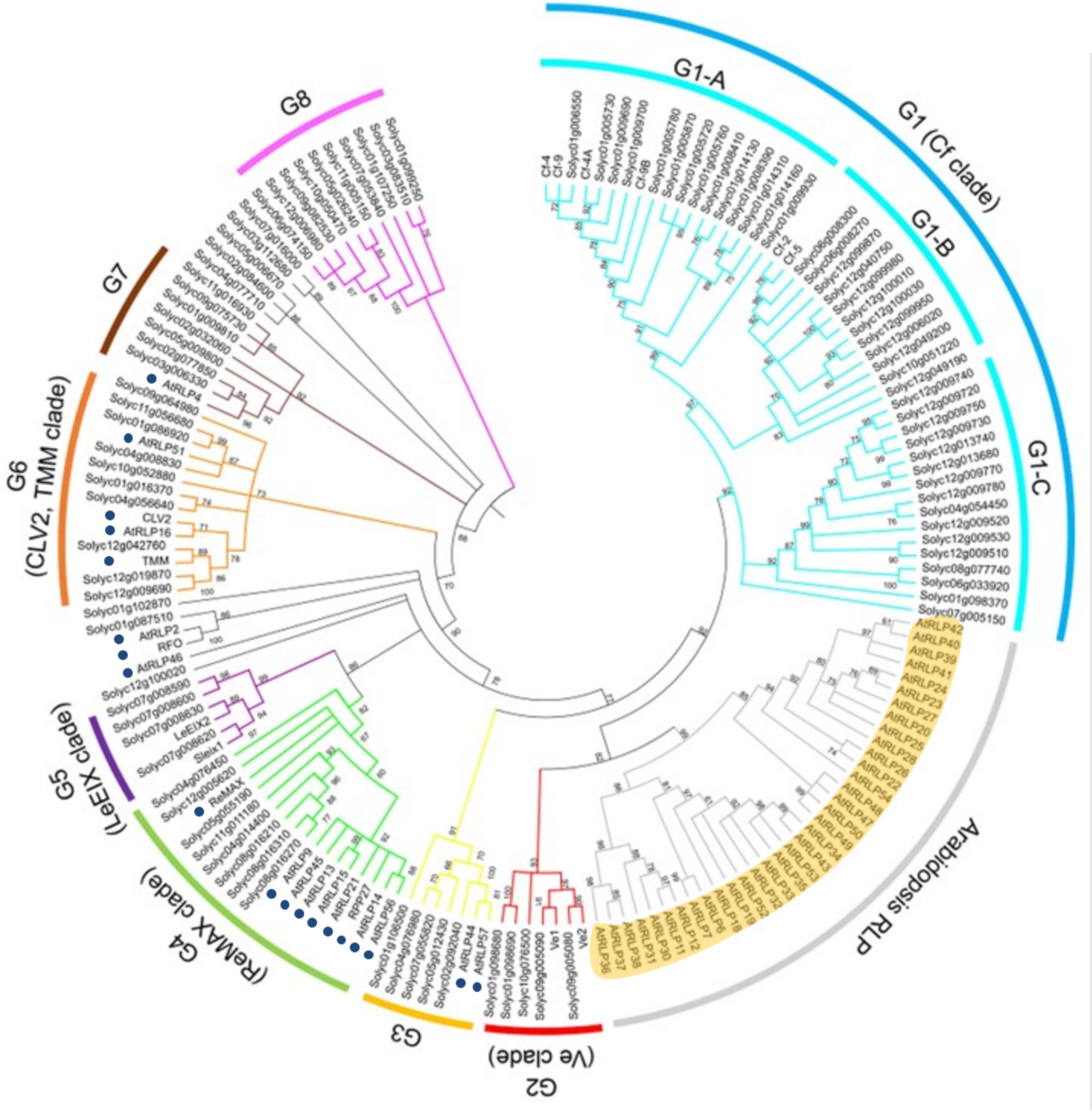

Figure S1: The phylogenetic tree of tomato and Arabidopsis RLPs.
The phylogenetic analysis was published by Kang and Yeom 2018 based on the C3-F domains. The Arabidopsis prRLPs (highlighted in yellow) form a monophyletic clade, whereas the Arabidopsis bRLPs cluster as poly- and paraphyletic groups with the annotated tomato RLPs (marked with blue dots). Bootstrap values > 60% are indicated above branches.
