# Supplementary material for: Multi-omics approach highlights differences between RLP classes in Arabidopsis thaliana
Source: BMC Genomics. 2021 Jul 20;22:557. doi: 10.1186/s12864-021-07855-0 (PMC8290556; doi:10.1186/s12864-021-07855-0)
Supplement: Supplementary file 2 — Additional file 2: [file 12864_2021_7855_MOESM2_ESM.pdf]

single genes	
**RLP**	**ATG**
AtRLP1	AT1G07390
AtRLP17	AT1G80080
AtRLP29	AT2G42800
AtRLP36	AT3G23010
AtRLP39	AT3G24900
AtRLP43	AT3G28890
AtRLP44	AT3G49750
AtRLP45	AT3G53240
AtRLP46	AT4G04220
AtRLP51	AT4G18760
AtRLP52	AT5G25910
AtRLP53	AT5G27060
AtRLP54	AT5G40170
AtRLP55	AT5G45770
AtRLP56	AT5G49290
AtRLP57	AT5G65830

paired genes	
**RLP**	**ATG**
AtRLP2	AT1G17240
AtRLP3	AT1G17250
AtRLP4	AT1G28340
AtRLP5	AT1G34290
AtRLP6	AT1G45616
AtRLP7	AT1G47890
AtRLP11	AT1G71390
AtRLP12	AT1G71400
AtRLP18	AT2G15040
AtRLP19	AT2G15080
AtRLP20	AT2G25440
AtRLP21	AT2G25470
AtRLP22	AT2G32660
AtRLP23	AT2G32680
AtRLP30	AT3G05360
AtRLP31	AT3G05370
AtRLP32	AT3G05650
AtRLP33	AT3G05660
AtRLP34	AT3G11010
AtRLP35	AT3G11080
AtRLP37	AT3G23110
AtRLP38	AT3G23120

higher order groups	
**RLP**	**ATG**
AtRLP8	AT1G54480
AtRLP9	AT1G58190
AtRLP10	AT1G65380
AtRLP13	AT1G74170
AtRLP14	AT1G74180
AtRLP15	AT1G74190
AtRLP16	AT1G74200
AtRLP24	AT2G33020
AtRLP25	AT2G33030
AtRLP26	AT2G33050
AtRLP27	AT2G33060
AtRLP28	AT2G33080
AtRLP40	AT3G24982
AtRLP41	AT3G25010
AtRLP42	AT3G25020
AtRLP47	AT4G13810
AtRLP48	AT4G13880
AtRLP50	AT4G13920

Figure S2: Genomic organization of RLPs in Arabidopsis.
