# Supplementary material for: Multi-omics approach highlights differences between RLP classes in Arabidopsis thaliana
Source: BMC Genomics. 2021 Jul 20;22:557. doi: 10.1186/s12864-021-07855-0 (PMC8290556; doi:10.1186/s12864-021-07855-0)
Supplement: Supplementary file 3 — Additional file 3: [file 12864_2021_7855_MOESM3_ESM.pdf]

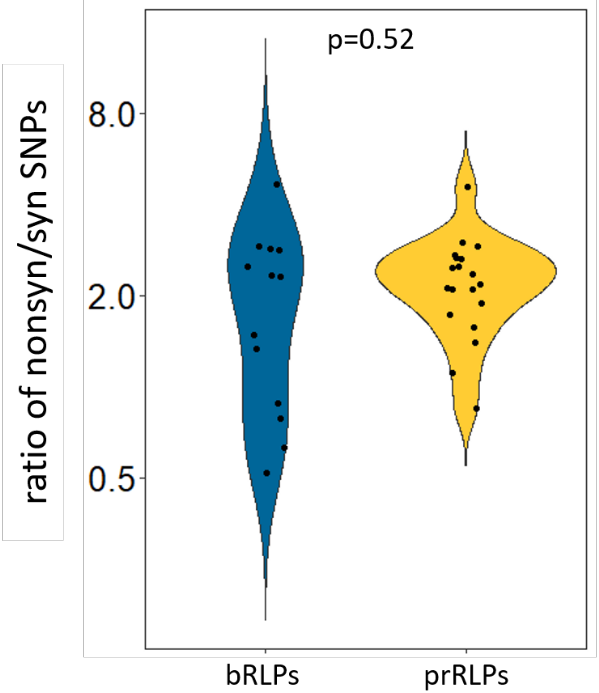

Figure S3: Ratio of non-synonymous over synonymous SNPs on a logarithmic scale (y axis) as calculated with PopGenome for the bRLPs and prRLPs (x axis).
