# Supplementary material for: Multi-omics approach highlights differences between RLP classes in Arabidopsis thaliana
Source: BMC Genomics. 2021 Jul 20;22:557. doi: 10.1186/s12864-021-07855-0 (PMC8290556; doi:10.1186/s12864-021-07855-0)
Supplement: Supplementary file 4 — Additional file 4: [file 12864_2021_7855_MOESM4_ESM.pdf]

Table S1: Hypothetical PRRs with associated pathogens that might harbour an immunogenic motif, which is recognized by the respective RLP.

RLP	pathogen	genera
RLP12	Plectosphaerella cucumerina	fungus
	Botrytis cinerea	fungus
	Blumeria graminis	fungus
RLP19	Blumeria graminis	fungus
	Plectosphaerella cucumerina	fungus
	Phytophthora infestans	oomycete
	Botrytis cinerea	fungus
RLP27	Pseudomonas syringae	bacteria
RLP35	Botrytis cinerea	fungus
	Colletotrichum incanum	fungus
	Pseudomonas syringae	bacteria
RLP37/38	Blumeria graminis	fungus
	Erysiphe cichoracearum	fungus
	Hyaloperonospora arabidopsidis	oomycete
	Colletotrichum incanum	fungus
RLP40	Pseudomonas syringae	bacteria
	Blumeria graminis	fungus
RLP41	Erysiphe cichoracearum	fungus
	Golovinomyces/Erysiphe orontii	fungus
	Plectosphaerella cucumerina	fungus
	Sclerotinia sclerotiorum	fungus
	Turnip mosaic virus	virus
RLP50	Blumeria graminis	fungus
	Golovinomyces/Erysiphe orontii	fungus
	Pseudomonas syringae	bacteria
	Sclerotinia sclerotiorum	fungus
RLP54	Botrytis cinerea	fungus
	Pseudomonas syringae	bacteria
