# Supplementary material for: Multi-omics approach highlights differences between RLP classes in Arabidopsis thaliana
Source: BMC Genomics. 2021 Jul 20;22:557. doi: 10.1186/s12864-021-07855-0 (PMC8290556; doi:10.1186/s12864-021-07855-0)
Supplement: Supplementary file 5 — Additional file 5: [file 12864_2021_7855_MOESM5_ESM.xz › SupplementaryData/Expression_data_genevestigator.pdf]

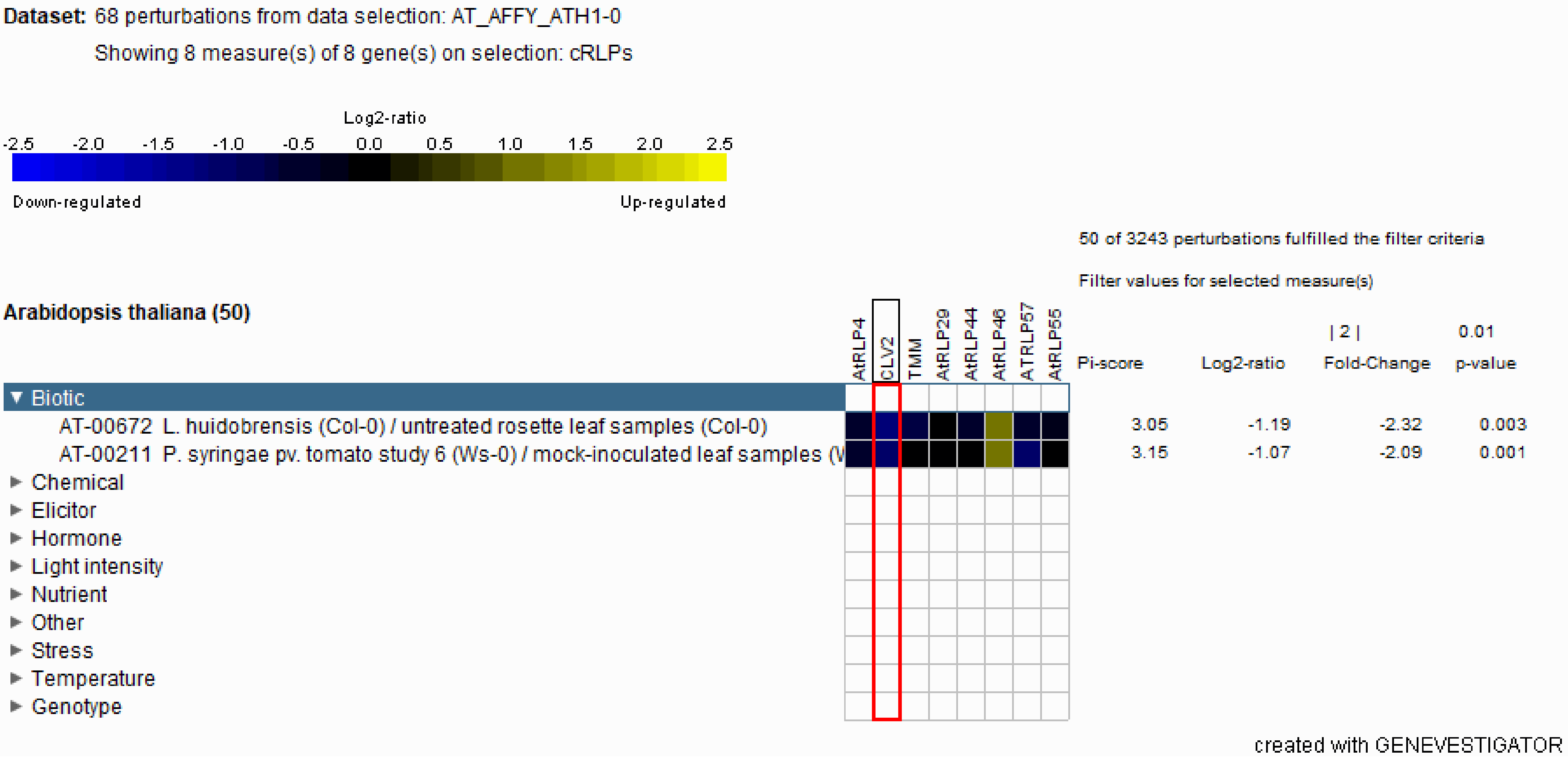

Dataset: 68 perturbations from data selection: AT_AFFY_ATH1-0
Showing 8 measure(s) of 8 gene(s) on selection: cRLPs
Log2-ratio
-2.5 -2.0 -1.5 -1.0 -0.5 0.0 0.5 1.0 1.5 2.0 2.5
Down-regulated
Up-regulated
50 of 3243 perturbations fulfilled the filter criteria
Filter values for selected measure(s)
Arabidopsis thaliana (50)
AtRLP4
CLV2
TMM
AtRLP29
AtRLP44
AtRLP46
ATRLP57
AtRLP55
| 2 |
0.01
Pi-score
Log2-ratio
Fold-Change
p-value
Biotic
AT-00672 L. huidobrensis (Col-0) / untreated rosette leaf samples (Col-0)
3.05 -1.19 -2.32 0.003
AT-00211 P. syringae pv. tomato study 6 (Ws-0) / mock-inoculated leaf samples (W
3.15 -1.07 -2.09 0.001
Chemical
Elicitor
Hormone
Light intensity
Nutrient
Other
Stress
Temperature
Genotype
created with GENEVESTIGATOR

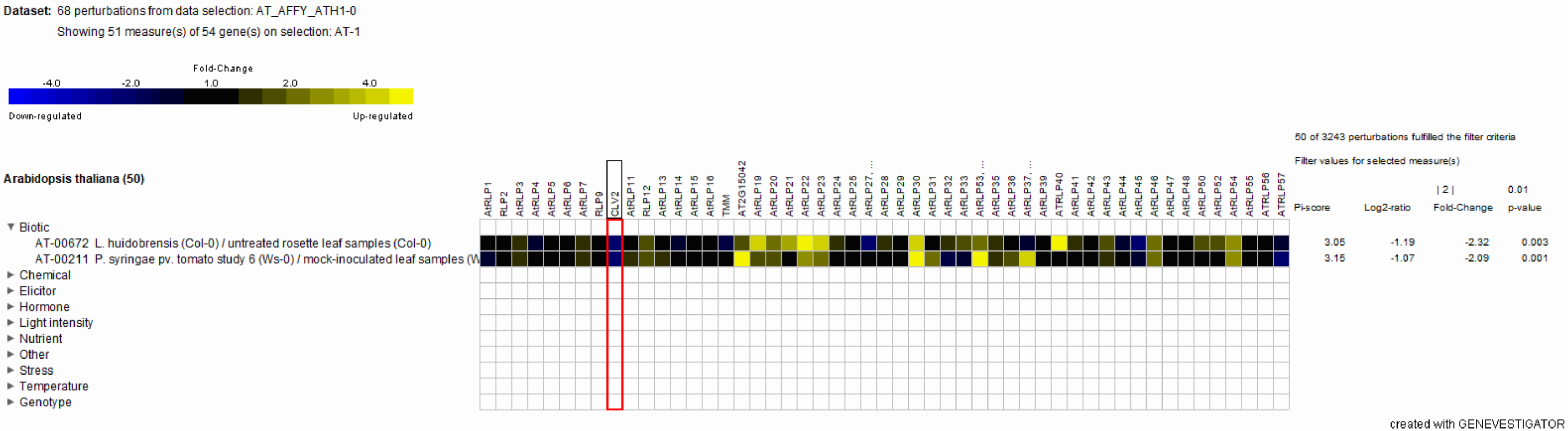
Dataset: 68 perturbations from data selection: AT_AFFY_ATH1-0
Showing 51 measure(s) of 54 gene(s) on selection: AT-1
Fold-Change
-4.0
-2.0
1.0
2.0
4.0
Down-regulated
Up-regulated
50 of 3243 perturbations fulfilled the filter criteria
Filter values for selected measure(s)
Arabidopsis thaliana (50)
AtRLP1 RLP2 AtRLP3 AtRLP4 AtRLP5 AtRLP6 AtRLP7 RLP9 CLV2 AtRLP11 RLP12 AtRLP13 AtRLP14 AtRLP15 AtRLP16 TMM AT2G15042 AtRLP19 AtRLP20 AtRLP21 AtRLP22 AtRLP23 AtRLP24 AtRLP25 AtRLP27, ... AtRLP28 AtRLP29 AtRLP30 AtRLP31 AtRLP32 AtRLP33 AtRLP53, ... AtRLP35 AtRLP36 AtRLP37, ... AtRLP39 ATRLP40 AtRLP41 AtRLP42 AtRLP43 AtRLP44 AtRLP45 AtRLP46 AtRLP47 AtRLP48 AtRLP50 AtRLP52 AtRLP54 AtRLP55 ATRLP56 ATRLP57
Pi-score
Log2-ratio
| 2 |
Fold-Change
0.01
p-value
Biotic
AT-00672 L. huidobrensis (Col-0) / untreated rosette leaf samples (Col-0)
3.05
-1.19
-2.32
0.003
AT-00211 P. syringae pv. tomato study 6 (Ws-0) / mock-inoculated leaf samples (W
3.15
-1.07
-2.09
0.001
Chemical
Elicitor
Hormone
Light intensity
Nutrient
Other
Stress
Temperature
Genotype
created with GENEVESTIGATOR

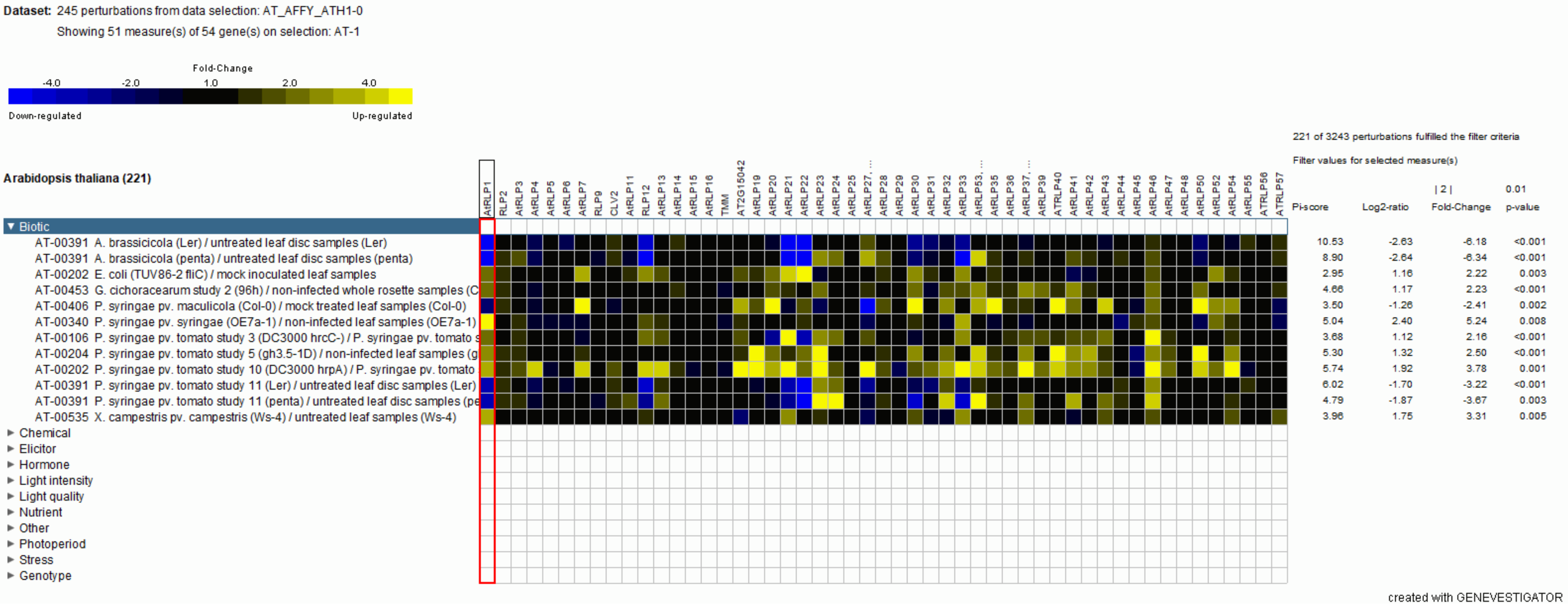

Dataset: 245 perturbations from data selection: AT_AFFY_ATH1-0
Showing 51 measure(s) of 54 gene(s) on selection: AT-1
Fold-Change
-4.0 -2.0 1.0 2.0 4.0
Down-regulated
Up-regulated
221 of 3243 perturbations fulfilled the filter criteria
Filter values for selected measure(s)
Arabidopsis thaliana (221)
AtRLP1 RLP2 AtRLP3 AtRLP4 AtRLP5 AtRLP6 AtRLP7 RLP9 CLV2 AtRLP11 RLP12 AtRLP13 AtRLP14 AtRLP15 AtRLP16 TMM AT2G15042 AtRLP19 AtRLP20 AtRLP21 AtRLP22 AtRLP23 AtRLP24 AtRLP25 AtRLP27, ... AtRLP28 AtRLP29 AtRLP30 AtRLP31 AtRLP32 AtRLP33 AtRLP53, ... AtRLP35 AtRLP36 AtRLP37, ... AtRLP39 ATRLP40 AtRLP41 AtRLP42 AtRLP43 AtRLP44 AtRLP45 AtRLP46 AtRLP47 AtRLP48 AtRLP50 AtRLP52 AtRLP54 AtRLP55 ATRLP56 ATRLP57
Pi-score
Log2-ratio
| 2 |
Fold-Change
0.01
p-value
Biotic
AT-00391 A. brassicicola (Ler) / untreated leaf disc samples (Ler) 10.53 -2.63 -6.18 <0.001
AT-00391 A. brassicicola (penta) / untreated leaf disc samples (penta) 8.90 -2.64 -6.34 <0.001
AT-00202 E. coli (TUV86-2 fliC) / mock inoculated leaf samples 2.95 1.16 2.22 0.003
AT-00453 G. cichoracearum study 2 (96h) / non-infected whole rosette samples (C 4.66 1.17 2.23 <0.001
AT-00406 P. syringae pv. maculicola (Col-0) / mock treated leaf samples (Col-0) 3.50 -1.26 -2.41 0.002
AT-00340 P. syringae pv. syringae (OE7a-1) / non-infected leaf samples (OE7a-1) 5.04 2.40 5.24 0.008
AT-00106 P. syringae pv. tomato study 3 (DC3000 hrcC-) / P. syringae pv. tomato s 3.68 1.12 2.16 <0.001
AT-00204 P. syringae pv. tomato study 5 (gh3.5-1D) / non-infected leaf samples (g 5.30 1.32 2.50 <0.001
AT-00202 P. syringae pv. tomato study 10 (DC3000 hrpA) / P. syringae pv. tomato 5.74 1.92 3.78 0.001
AT-00391 P. syringae pv. tomato study 11 (Ler) / untreated leaf disc samples (Ler) 6.02 -1.70 -3.22 <0.001
AT-00391 P. syringae pv. tomato study 11 (penta) / untreated leaf disc samples (pe 4.79 -1.87 -3.67 0.003
AT-00535 X. campestris pv. campestris (Ws-4) / untreated leaf samples (Ws-4) 3.96 1.75 3.31 0.005
Chemical
Elicitor
Hormone
Light intensity
Light quality
Nutrient
Other
Photoperiod
Stress
Genotype
created with GENEVESTIGATOR

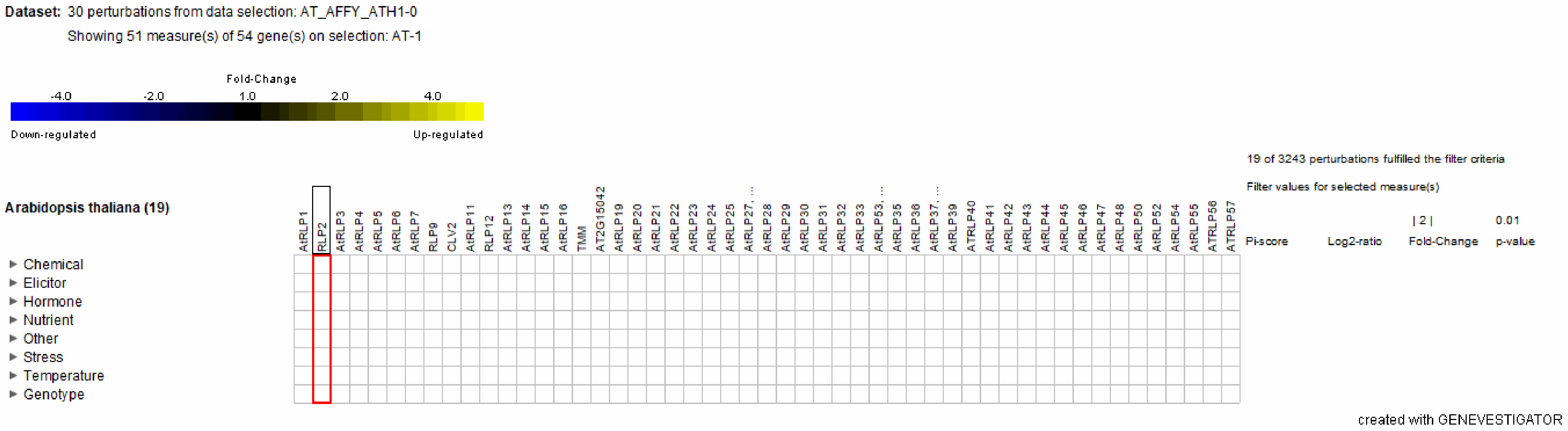

Dataset: 30 perturbations from data selection: AT_AFFY_ATH1-0
Showing 51 measure(s) of 54 gene(s) on selection: AT-1
Fold-Change
-4.0
-2.0
1.0
2.0
4.0
Down-regulated
Up-regulated
19 of 3243 perturbations fulfilled the filter criteria
Filter values for selected measure(s)
Arabidopsis thaliana (19)
AtRLP1
RLP2
AtRLP3
AtRLP4
AtRLP5
AtRLP6
AtRLP7
RLP9
CLV2
AtRLP11
RLP12
AtRLP13
AtRLP14
AtRLP15
AtRLP16
TMM
AT2G15042
AtRLP19
AtRLP20
AtRLP21
AtRLP22
AtRLP23
AtRLP24
AtRLP25
AtRLP27, ...
AtRLP28
AtRLP29
AtRLP30
AtRLP31
AtRLP32
AtRLP33
AtRLP53, ...
AtRLP35
AtRLP36
AtRLP37, ...
AtRLP39
ATRLP40
AtRLP41
AtRLP42
AtRLP43
AtRLP44
AtRLP45
AtRLP46
AtRLP47
AtRLP48
AtRLP50
AtRLP52
AtRLP54
AtRLP55
ATRLP56
ATRLP57
Pi-score
Log2-ratio
| 2 |
Fold-Change
0.01
p-value
Chemical
Elicitor
Hormone
Nutrient
Other
Stress
Temperature
Genotype
created with GENEVESTIGATOR

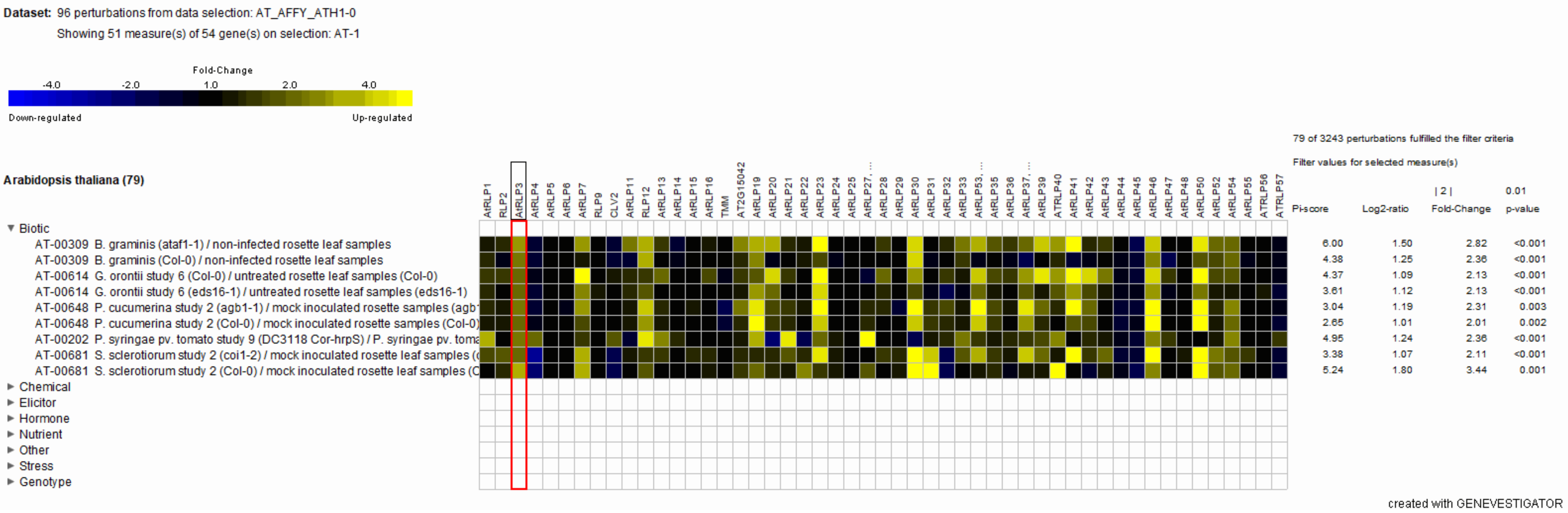
Dataset: 96 perturbations from data selection: AT_AFFY_ATH1-0
Showing 51 measure(s) of 54 gene(s) on selection: AT-1
Fold-Change
-4.0 -2.0 1.0 2.0 4.0
Down-regulated
Up-regulated
79 of 3243 perturbations fulfilled the filter criteria
Filter values for selected measure(s)
Arabidopsis thaliana (79)
AtRLP1 RLP2 AtRLP3 AtRLP4 AtRLP5 AtRLP6 AtRLP7 RLP9 CLV2 AtRLP11 RLP12 AtRLP13 AtRLP14 AtRLP15 AtRLP16 TMM AT2G15042 AtRLP19 AtRLP20 AtRLP21 AtRLP22 AtRLP23 AtRLP24 AtRLP25 AtRLP27, ... AtRLP28 AtRLP29 AtRLP30 AtRLP31 AtRLP32 AtRLP33 AtRLP53, ... AtRLP35 AtRLP36 AtRLP37, ... AtRLP39 ATRLP40 AtRLP41 AtRLP42 AtRLP43 AtRLP44 AtRLP45 AtRLP46 AtRLP47 AtRLP48 AtRLP50 AtRLP52 AtRLP54 AtRLP55 ATRLP56 ATRLP57
| 2 | 0.01
Pi-score Log2-ratio Fold-Change p-value
Biotic
AT-00309 B. graminis (ataf1-1) / non-infected rosette leaf samples 6.00 1.50 2.82 <0.001
AT-00309 B. graminis (Col-0) / non-infected rosette leaf samples 4.38 1.25 2.36 <0.001
AT-00614 G. orontii study 6 (Col-0) / untreated rosette leaf samples (Col-0) 4.37 1.09 2.13 <0.001
AT-00614 G. orontii study 6 (eds16-1) / untreated rosette leaf samples (eds16-1) 3.61 1.12 2.13 <0.001
AT-00648 P. cucumerina study 2 (agb1-1) / mock inoculated rosette samples (agb1 3.04 1.19 2.31 0.003
AT-00648 P. cucumerina study 2 (Col-0) / mock inoculated rosette samples (Col-0) 2.65 1.01 2.01 0.002
AT-00202 P. syringae pv. tomato study 9 (DC3118 Cor-hrpS) / P. syringae pv. toma 4.95 1.24 2.36 <0.001
AT-00681 S. sclerotiorum study 2 (coi1-2) / mock inoculated rosette leaf samples (c 3.38 1.07 2.11 <0.001
AT-00681 S. sclerotiorum study 2 (Col-0) / mock inoculated rosette leaf samples (C 5.24 1.80 3.44 0.001
Chemical
Elicitor
Hormone
Nutrient
Other
Stress
Genotype
created with GENEVESTIGATOR

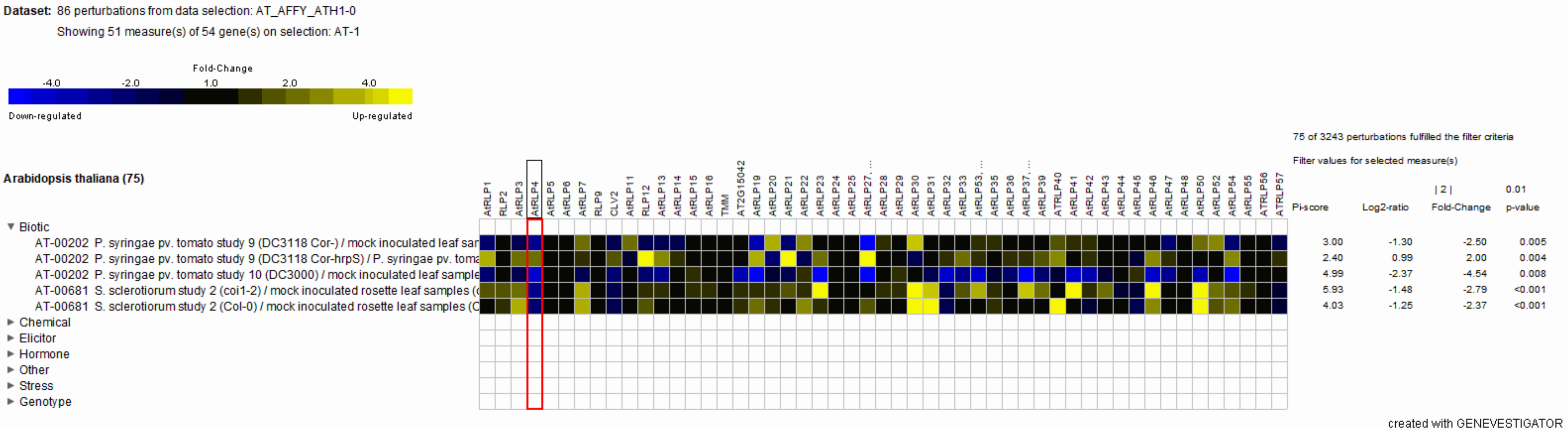

Dataset: 86 perturbations from data selection: AT_AFFY_ATH1-0
Showing 51 measure(s) of 54 gene(s) on selection: AT-1
Fold-Change
-4.0
-2.0
1.0
2.0
4.0
Down-regulated
Up-regulated
75 of 3243 perturbations fulfilled the filter criteria
Filter values for selected measure(s)
Arabidopsis thaliana (75)
AtRLP1
RLP2
AtRLP3
AtRLP4
AtRLP5
AtRLP6
AtRLP7
RLP9
CLV2
AtRLP11
RLP12
AtRLP13
AtRLP14
AtRLP15
AtRLP16
TMM
AT2G15042
AtRLP19
AtRLP20
AtRLP21
AtRLP22
AtRLP23
AtRLP24
AtRLP25
AtRLP27, ...
AtRLP28
AtRLP29
AtRLP30
AtRLP31
AtRLP32
AtRLP33
AtRLP53, ...
AtRLP35
AtRLP36
AtRLP37, ...
AtRLP39
ATRLP40
AtRLP41
AtRLP42
AtRLP43
AtRLP44
AtRLP45
AtRLP46
AtRLP47
AtRLP48
AtRLP50
AtRLP52
AtRLP54
AtRLP55
ATRLP56
ATRLP57
| 2 |
0.01
Pi-score
Log2-ratio
Fold-Change
p-value
Biotic
AT-00202 P. syringae pv. tomato study 9 (DC3118 Cor-) / mock inoculated leaf sar
AT-00202 P. syringae pv. tomato study 9 (DC3118 Cor-hrpS) / P. syringae pv. toma
AT-00202 P. syringae pv. tomato study 10 (DC3000) / mock inoculated leaf sample
AT-00681 S. sclerotiorum study 2 (coi1-2) / mock inoculated rosette leaf samples (
AT-00681 S. sclerotiorum study 2 (Col-0) / mock inoculated rosette leaf samples (C
3.00 -1.30 -2.50 0.005
2.40 0.99 2.00 0.004
4.99 -2.37 -4.54 0.008
5.93 -1.48 -2.79 <0.001
4.03 -1.25 -2.37 <0.001
Chemical
Elicitor
Hormone
Other
Stress
Genotype
created with GENEVESTIGATOR

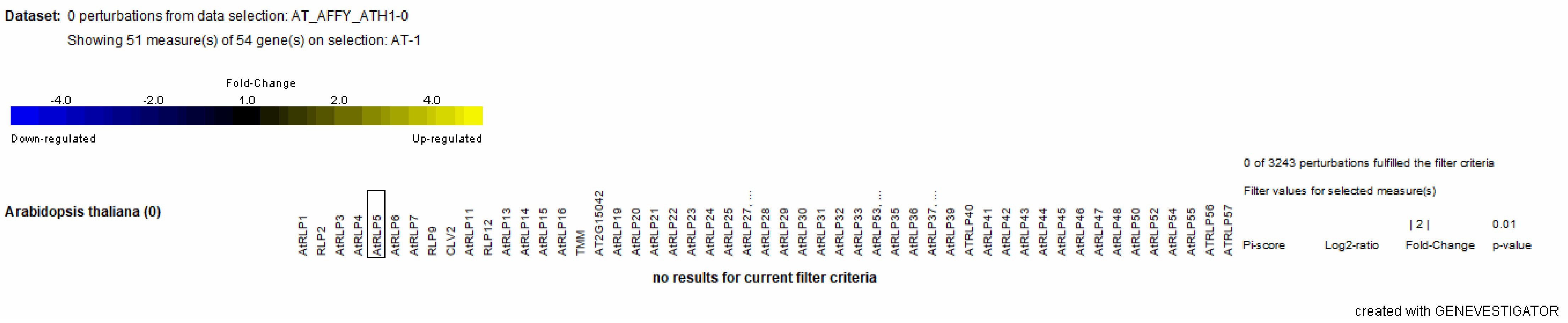

Dataset: 0 perturbations from data selection: AT_AFFY_ATH1-0
Showing 51 measure(s) of 54 gene(s) on selection: AT-1
Fold-Change
-4.0
-2.0
1.0
2.0
4.0
Down-regulated
Up-regulated
0 of 3243 perturbations fulfilled the filter criteria
Filter values for selected measure(s)
Arabidopsis thaliana (0)
AtRLP1
RLP2
AtRLP3
AtRLP4
AtRLP5
AtRLP6
AtRLP7
RLP9
CLV2
AtRLP11
RLP12
AtRLP13
AtRLP14
AtRLP15
AtRLP16
TMM
AT2G15042
AtRLP19
AtRLP20
AtRLP21
AtRLP22
AtRLP23
AtRLP24
AtRLP25
AtRLP27, ...
AtRLP28
AtRLP29
AtRLP30
AtRLP31
AtRLP32
AtRLP33
AtRLP53, ...
AtRLP35
AtRLP36
AtRLP37, ...
AtRLP39
ATRLP40
AtRLP41
AtRLP42
AtRLP43
AtRLP44
AtRLP45
AtRLP46
AtRLP47
AtRLP48
AtRLP50
AtRLP52
AtRLP54
AtRLP55
ATRLP56
ATRLP57
Pi-score
Log2-ratio
| 2 |
Fold-Change
0.01
p-value
no results for current filter criteria
created with GENEVESTIGATOR

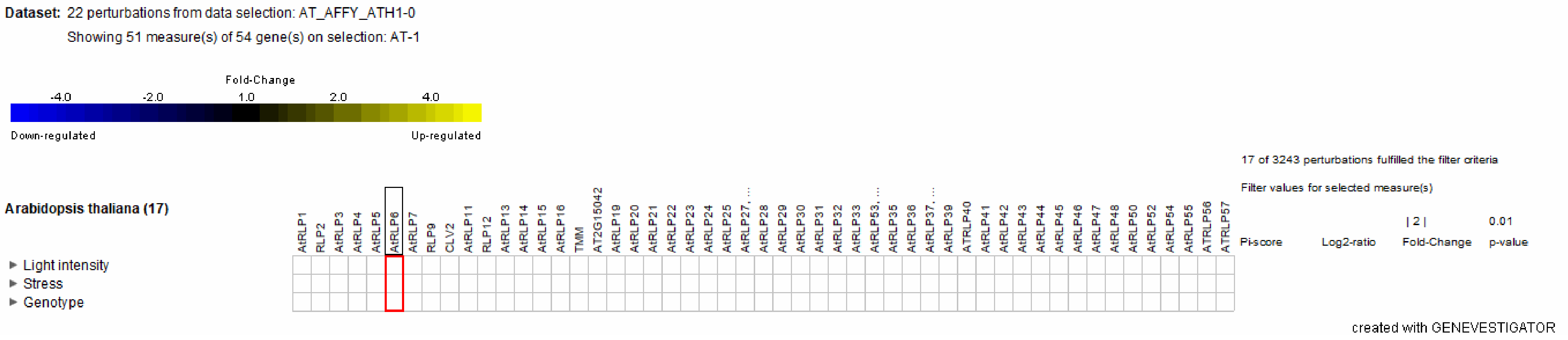
Dataset: 22 perturbations from data selection: AT_AFFY_ATH1-0
Showing 51 measure(s) of 54 gene(s) on selection: AT-1
Fold-Change
-4.0
-2.0
1.0
2.0
4.0
Down-regulated
Up-regulated
17 of 3243 perturbations fulfilled the filter criteria
Filter values for selected measure(s)
Arabidopsis thaliana (17)
AtRLP1
RLP2
AtRLP3
AtRLP4
AtRLP5
AtRLP6
AtRLP7
RLP9
CLV2
AtRLP11
RLP12
AtRLP13
AtRLP14
AtRLP15
AtRLP16
TMM
AT2G15042
AtRLP19
AtRLP20
AtRLP21
AtRLP22
AtRLP23
AtRLP24
AtRLP25
AtRLP27, ...
AtRLP28
AtRLP29
AtRLP30
AtRLP31
AtRLP32
AtRLP33
AtRLP53, ...
AtRLP35
AtRLP36
AtRLP37, ...
AtRLP39
ATRLP40
AtRLP41
AtRLP42
AtRLP43
AtRLP44
AtRLP45
AtRLP46
AtRLP47
AtRLP48
AtRLP50
AtRLP52
AtRLP54
AtRLP55
ATRLP56
ATRLP57
Pi-score
Log2-ratio
| 2 |
Fold-Change
0.01
p-value
Light intensity
Stress
Genotype
created with GENEVESTIGATOR

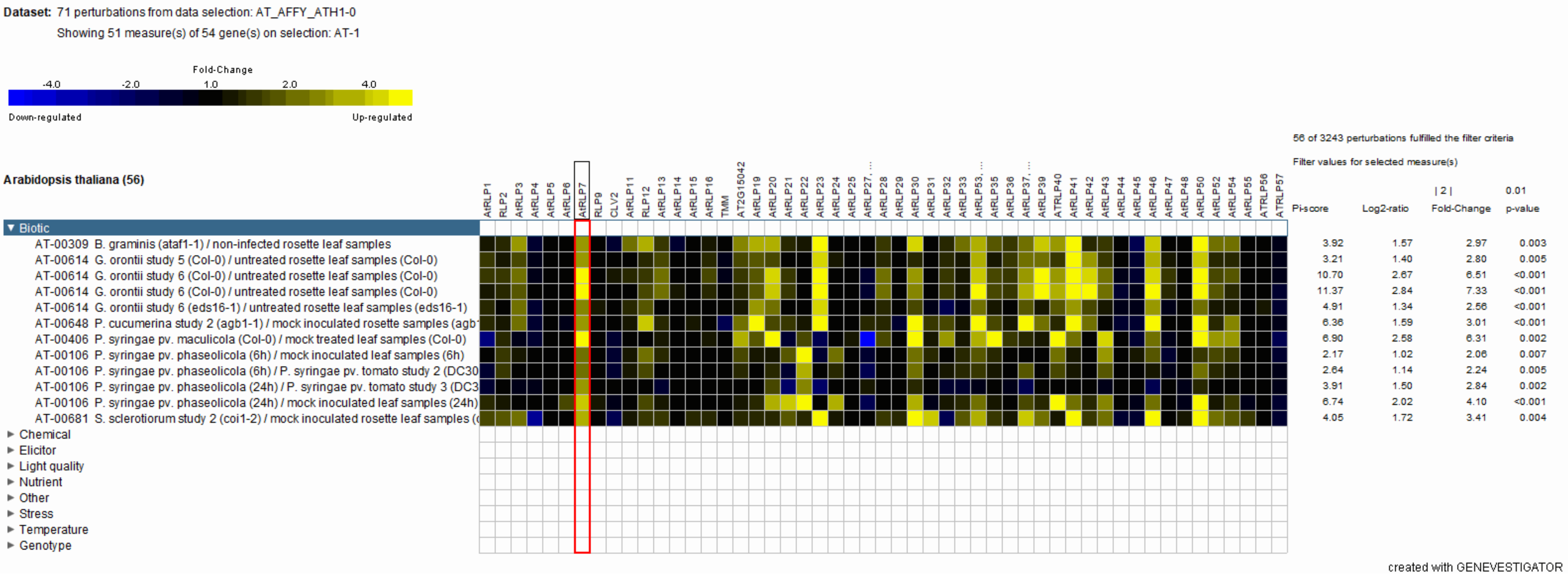

Perturbation	Pi-score	Log2-ratio	Fold-Change (|2|)	p-value (0.01)
AT-00309 B. graminis (ataf1-1) / non-infected rosette leaf samples	3.92	1.57	2.97	0.003
AT-00614 G. orontii study 5 (Col-0) / untreated rosette leaf samples (Col-0)	3.21	1.40	2.80	0.005
AT-00614 G. orontii study 6 (Col-0) / untreated rosette leaf samples (Col-0)	10.70	2.67	6.51	<0.001
AT-00614 G. orontii study 6 (Col-0) / untreated rosette leaf samples (Col-0)	11.37	2.84	7.33	<0.001
AT-00614 G. orontii study 6 (eds16-1) / untreated rosette leaf samples (eds16-1)	4.91	1.34	2.56	<0.001
AT-00648 P. cucumerina study 2 (agb1-1) / mock inoculated rosette samples (agb1	6.36	1.59	3.01	<0.001
AT-00406 P. syringae pv. maculicola (Col-0) / mock treated leaf samples (Col-0)	6.90	2.58	6.31	0.002
AT-00106 P. syringae pv. phaseolicola (6h) / mock inoculated leaf samples (6h)	2.17	1.02	2.06	0.007
AT-00106 P. syringae pv. phaseolicola (6h) / P. syringae pv. tomato study 2 (DC30	2.64	1.14	2.24	0.005
AT-00106 P. syringae pv. phaseolicola (24h) / P. syringae pv. tomato study 3 (DC3	3.91	1.50	2.84	0.002
AT-00106 P. syringae pv. phaseolicola (24h) / mock inoculated leaf samples (24h)	6.74	2.02	4.10	<0.001
AT-00681 S. sclerotiorum study 2 (coi1-2) / mock inoculated rosette leaf samples ((	4.05	1.72	3.41	0.004

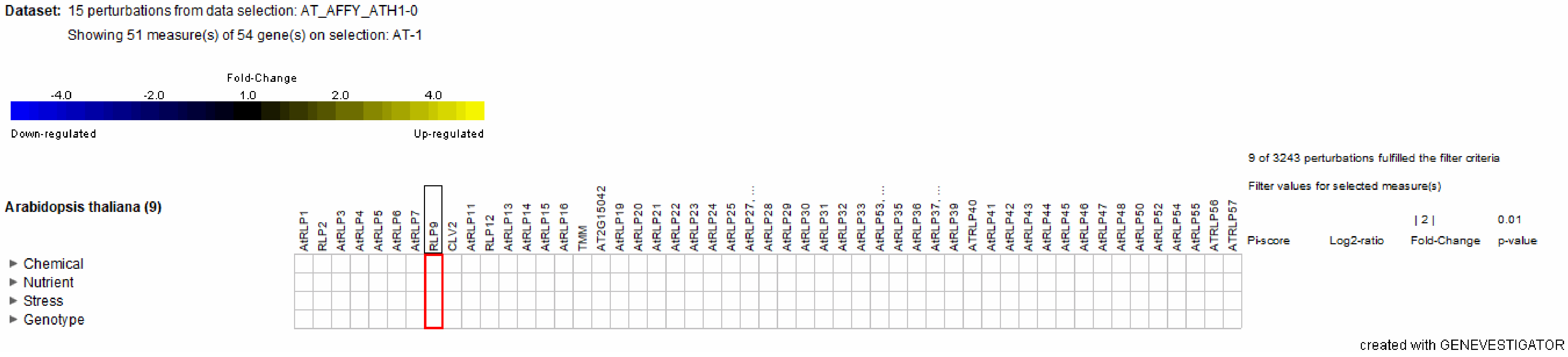
Dataset: 15 perturbations from data selection: AT_AFFY_ATH1-0
Showing 51 measure(s) of 54 gene(s) on selection: AT-1
Fold-Change
-4.0
-2.0
1.0
2.0
4.0
Down-regulated
Up-regulated
9 of 3243 perturbations fulfilled the filter criteria
Filter values for selected measure(s)
Arabidopsis thaliana (9)
AtRLP1
RLP2
AtRLP3
AtRLP4
AtRLP5
AtRLP6
AtRLP7
RLP9
CLV2
AtRLP11
RLP12
AtRLP13
AtRLP14
AtRLP15
AtRLP16
TMM
AT2G15042
AtRLP19
AtRLP20
AtRLP21
AtRLP22
AtRLP23
AtRLP24
AtRLP25
AtRLP27, ...
AtRLP28
AtRLP29
AtRLP30
AtRLP31
AtRLP32
AtRLP33
AtRLP53, ...
AtRLP35
AtRLP36
AtRLP37, ...
AtRLP39
ATRLP40
AtRLP41
AtRLP42
AtRLP43
AtRLP44
AtRLP45
AtRLP46
AtRLP47
AtRLP48
AtRLP50
AtRLP52
AtRLP54
AtRLP55
ATRLP56
ATRLP57
Pi-score
Log2-ratio
| 2 |
Fold-Change
0.01
p-value
Chemical
Nutrient
Stress
Genotype
created with GENEVESTIGATOR

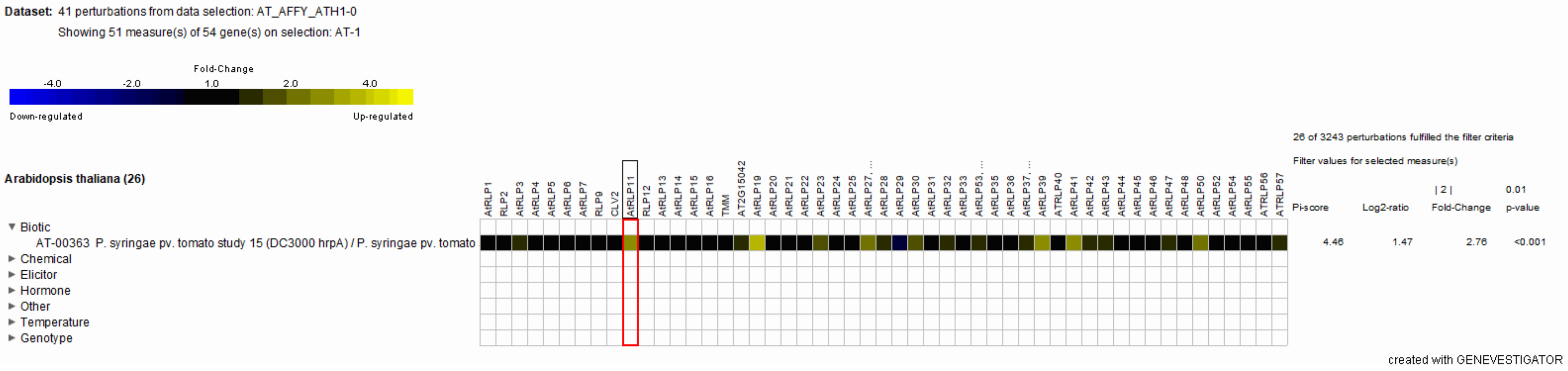

Dataset: 41 perturbations from data selection: AT_AFFY_ATH1-0
Showing 51 measure(s) of 54 gene(s) on selection: AT-1
Fold-Change
-4.0
-2.0
1.0
2.0
4.0
Down-regulated
Up-regulated
26 of 3243 perturbations fulfilled the filter criteria
Filter values for selected measure(s)
Arabidopsis thaliana (26)
AtRLP1 RLP2 AtRLP3 AtRLP4 AtRLP5 AtRLP6 AtRLP7 RLP9 CLV2 AtRLP11 RLP12 AtRLP13 AtRLP14 AtRLP15 AtRLP16 TMM AT2G15042 AtRLP19 AtRLP20 AtRLP21 AtRLP22 AtRLP23 AtRLP24 AtRLP25 AtRLP27, ... AtRLP28 AtRLP29 AtRLP30 AtRLP31 AtRLP32 AtRLP33 AtRLP53, ... AtRLP35 AtRLP36 AtRLP37, ... AtRLP39 ATRLP40 AtRLP41 AtRLP42 AtRLP43 AtRLP44 AtRLP45 AtRLP46 AtRLP47 AtRLP48 AtRLP50 AtRLP52 AtRLP54 AtRLP55 ATRLP56 ATRLP57
Pi-score
Log2-ratio
| 2 |
Fold-Change
0.01
p-value
Biotic
AT-00363 P. syringae pv. tomato study 15 (DC3000 hrpA) / P. syringae pv. tomato
4.46
1.47
2.76
<0.001
Chemical
Elicitor
Hormone
Other
Temperature
Genotype
created with GENEVESTIGATOR

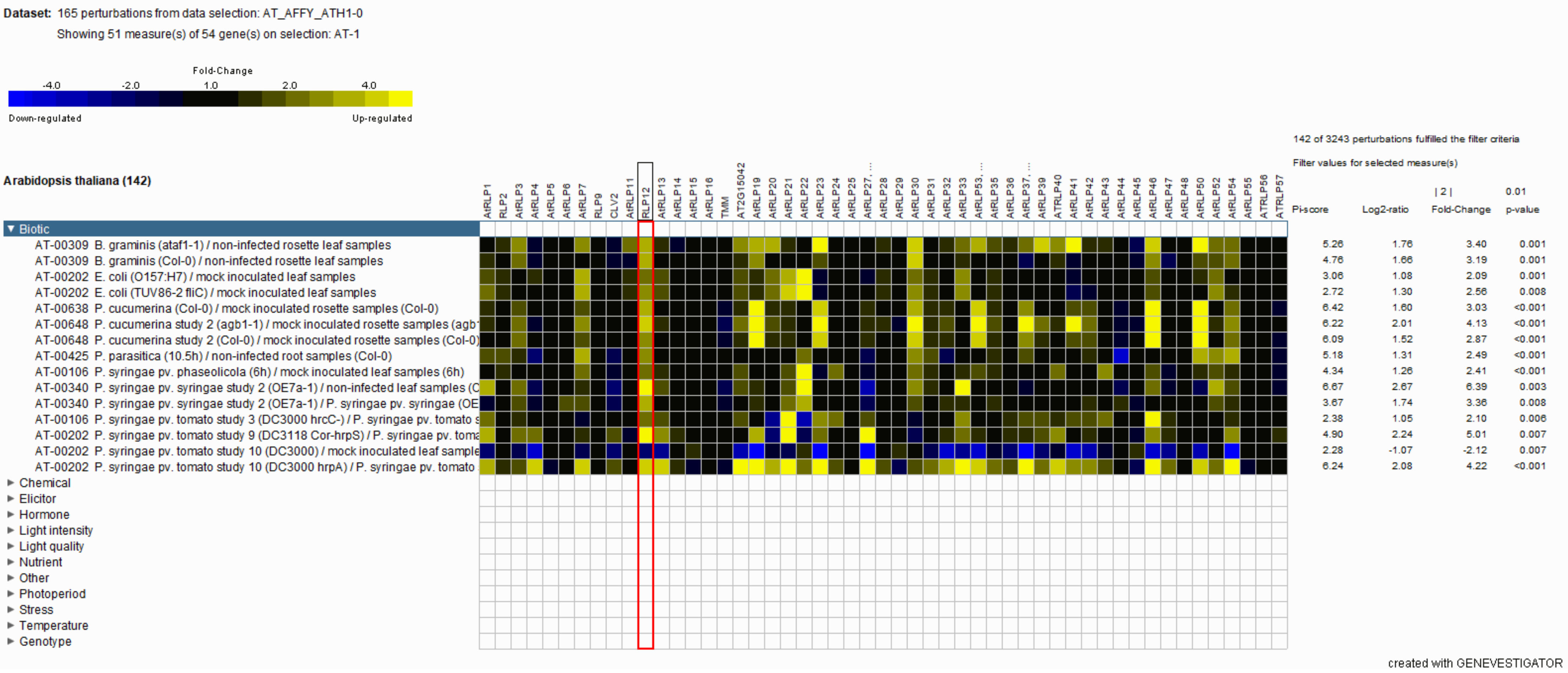

Dataset: 165 perturbations from data selection: AT_AFFY_ATH1-0
Showing 51 measure(s) of 54 gene(s) on selection: AT-1
Fold-Change
-4.0
-2.0
1.0
2.0
4.0
Down-regulated
Up-regulated
142 of 3243 perturbations fulfilled the filter criteria
Filter values for selected measure(s)
Arabidopsis thaliana (142)
AtRLP1 RLP2 AtRLP3 AtRLP4 AtRLP5 AtRLP6 AtRLP7 RLP9 CLV2 AtRLP11 RLP12 AtRLP13 AtRLP14 AtRLP15 AtRLP16 TMM AT2G15042 AtRLP19 AtRLP20 AtRLP21 AtRLP22 AtRLP23 AtRLP24 AtRLP25 AtRLP27, ... AtRLP28 AtRLP29 AtRLP30 AtRLP31 AtRLP32 AtRLP33 AtRLP53, ... AtRLP35 AtRLP36 AtRLP37, ... AtRLP39 ATRLP40 AtRLP41 AtRLP42 AtRLP43 AtRLP44 AtRLP45 AtRLP46 AtRLP47 AtRLP48 AtRLP50 AtRLP52 AtRLP54 AtRLP55 ATRLP56 ATRLP57
| 2 |
0.01
Pi-score
Log2-ratio
Fold-Change
p-value
Biotic
AT-00309 B. graminis (ataf1-1) / non-infected rosette leaf samples 5.26 1.76 3.40 0.001
AT-00309 B. graminis (Col-0) / non-infected rosette leaf samples 4.76 1.66 3.19 0.001
AT-00202 E. coli (O157:H7) / mock inoculated leaf samples 3.06 1.08 2.09 0.001
AT-00202 E. coli (TUV86-2 fliC) / mock inoculated leaf samples 2.72 1.30 2.56 0.008
AT-00638 P. cucumerina (Col-0) / mock inoculated rosette samples (Col-0) 6.42 1.60 3.03 <0.001
AT-00648 P. cucumerina study 2 (agb1-1) / mock inoculated rosette samples (agb1 6.22 2.01 4.13 <0.001
AT-00648 P. cucumerina study 2 (Col-0) / mock inoculated rosette samples (Col-0) 6.09 1.52 2.87 <0.001
AT-00425 P. parasitica (10.5h) / non-infected root samples (Col-0) 5.18 1.31 2.49 <0.001
AT-00106 P. syringae pv. phaseolicola (6h) / mock inoculated leaf samples (6h) 4.34 1.26 2.41 <0.001
AT-00340 P. syringae pv. syringae study 2 (OE7a-1) / non-infected leaf samples (C 6.67 2.67 6.39 0.003
AT-00340 P. syringae pv. syringae study 2 (OE7a-1) / P. syringae pv. syringae (OE 3.67 1.74 3.36 0.008
AT-00106 P. syringae pv. tomato study 3 (DC3000 hrcC-) / P. syringae pv. tomato s 2.38 1.05 2.10 0.006
AT-00202 P. syringae pv. tomato study 9 (DC3118 Cor-hrpS) / P. syringae pv. toma 4.90 2.24 5.01 0.007
AT-00202 P. syringae pv. tomato study 10 (DC3000) / mock inoculated leaf sample 2.28 -1.07 -2.12 0.007
AT-00202 P. syringae pv. tomato study 10 (DC3000 hrpA) / P. syringae pv. tomato 6.24 2.08 4.22 <0.001
Chemical
Elicitor
Hormone
Light intensity
Light quality
Nutrient
Other
Photoperiod
Stress
Temperature
Genotype
created with GENEVESTIGATOR

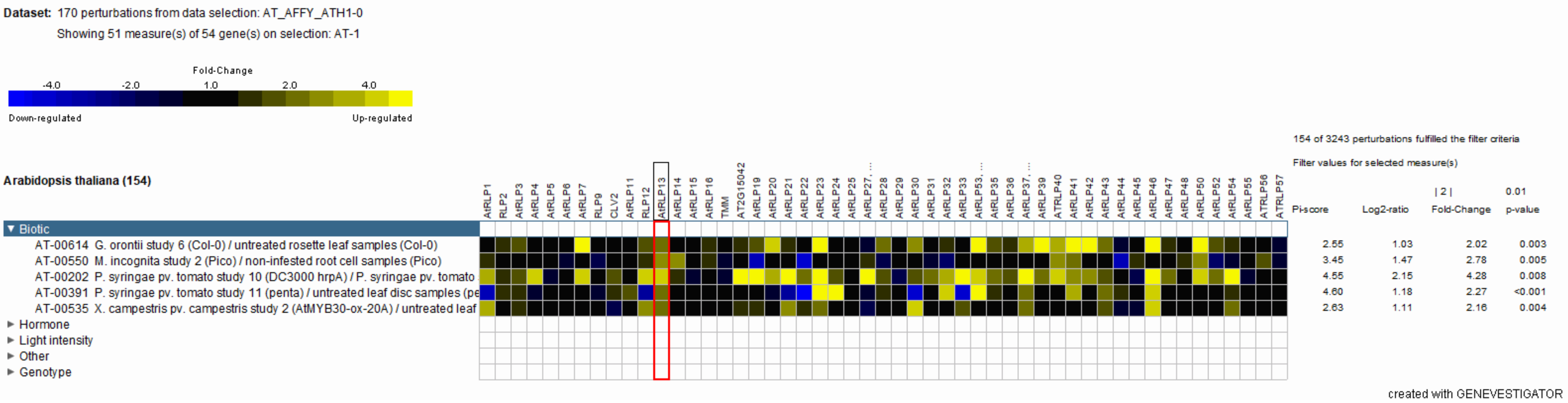

Dataset: 170 perturbations from data selection: AT_AFFY_ATH1-0
Showing 51 measure(s) of 54 gene(s) on selection: AT-1
Fold-Change
-4.0
-2.0
1.0
2.0
4.0
Down-regulated
Up-regulated
154 of 3243 perturbations fulfilled the filter criteria
Filter values for selected measure(s)
Arabidopsis thaliana (154)
AtRLP1
RLP2
AtRLP3
AtRLP4
AtRLP5
AtRLP6
AtRLP7
RLP9
CLV2
AtRLP11
RLP12
AtRLP13
AtRLP14
AtRLP15
AtRLP16
TMM
AT2G15042
AtRLP19
AtRLP20
AtRLP21
AtRLP22
AtRLP23
AtRLP24
AtRLP25
AtRLP27, ...
AtRLP28
AtRLP29
AtRLP30
AtRLP31
AtRLP32
AtRLP33
AtRLP53, ...
AtRLP35
AtRLP36
AtRLP37, ...
AtRLP39
ATRLP40
AtRLP41
AtRLP42
AtRLP43
AtRLP44
AtRLP45
AtRLP46
AtRLP47
AtRLP48
AtRLP50
AtRLP52
AtRLP54
AtRLP55
ATRLP56
ATRLP57
| 2 |
0.01
Pi-score
Log2-ratio
Fold-Change
p-value
Biotic
AT-00614 G. orontii study 6 (Col-0) / untreated rosette leaf samples (Col-0) 2.55 1.03 2.02 0.003
AT-00550 M. incognita study 2 (Pico) / non-infested root cell samples (Pico) 3.45 1.47 2.78 0.005
AT-00202 P. syringae pv. tomato study 10 (DC3000 hrpA) / P. syringae pv. tomato 4.55 2.15 4.28 0.008
AT-00391 P. syringae pv. tomato study 11 (penta) / untreated leaf disc samples (pe 4.60 1.18 2.27 <0.001
AT-00535 X. campestris pv. campestris study 2 (AtMYB30-ox-20A) / untreated leaf 2.63 1.11 2.16 0.004
Hormone
Light intensity
Other
Genotype
created with GENEVESTIGATOR

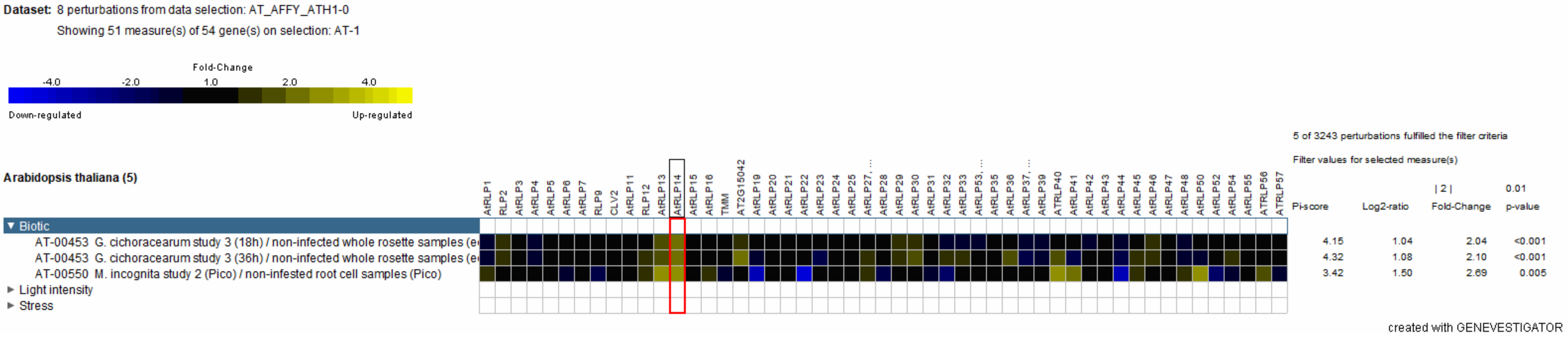
Dataset: 8 perturbations from data selection: AT_AFFY_ATH1-0
Showing 51 measure(s) of 54 gene(s) on selection: AT-1
Fold-Change
-4.0
-2.0
1.0
2.0
4.0
Down-regulated
Up-regulated
5 of 3243 perturbations fulfilled the filter criteria
Filter values for selected measure(s)
Arabidopsis thaliana (5)
AtRLP1
RLP2
AtRLP3
AtRLP4
AtRLP5
AtRLP6
AtRLP7
RLP9
CLV2
AtRLP11
RLP12
AtRLP13
AtRLP14
AtRLP15
AtRLP16
TMM
AT2G15042
AtRLP19
AtRLP20
AtRLP21
AtRLP22
AtRLP23
AtRLP24
AtRLP25
AtRLP27, ...
AtRLP28
AtRLP29
AtRLP30
AtRLP31
AtRLP32
AtRLP33
AtRLP53, ...
AtRLP35
AtRLP36
AtRLP37, ...
AtRLP39
ATRLP40
AtRLP41
AtRLP42
AtRLP43
AtRLP44
AtRLP45
AtRLP46
AtRLP47
AtRLP48
AtRLP50
AtRLP52
AtRLP54
AtRLP55
ATRLP56
ATRLP57
| 2 |
0.01
Pi-score
Log2-ratio
Fold-Change
p-value
Biotic
AT-00453 G. cichoracearum study 3 (18h) / non-infected whole rosette samples (e
4.15 1.04 2.04 <0.001
AT-00453 G. cichoracearum study 3 (36h) / non-infected whole rosette samples (e
4.32 1.08 2.10 <0.001
AT-00550 M. incognita study 2 (Pico) / non-infested root cell samples (Pico)
3.42 1.50 2.69 0.005
Light intensity
Stress
created with GENEVESTIGATOR

Dataset: 0 perturbations from data selection: AT_AFFY_ATH1-0

Showing 51 measure(s) of 54 gene(s) on selection: AT-1

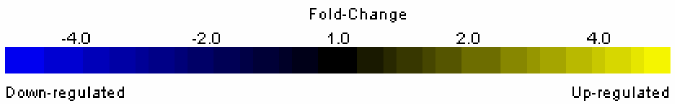

0 of 3243 perturbations fulfilled the filter criteria

Filter values for selected measure(s)

Arabidopsis thaliana (0)

AtRLP1, RLP2, AtRLP3, AtRLP4, AtRLP5, AtRLP6, AtRLP7, RLP9, CLV2, AtRLP11, RLP12, AtRLP13, AtRLP14, AtRLP15, AtRLP16, TMM, AT2G15042, AtRLP19, AtRLP20, AtRLP21, AtRLP22, AtRLP23, AtRLP24, AtRLP25, AtRLP27, ..., AtRLP28, AtRLP29, AtRLP30, AtRLP31, AtRLP32, AtRLP33, AtRLP53, ..., AtRLP35, AtRLP36, AtRLP37, ..., AtRLP39, ATRLP40, AtRLP41, AtRLP42, AtRLP43, AtRLP44, AtRLP45, AtRLP46, AtRLP47, AtRLP48, AtRLP50, AtRLP52, AtRLP54, AtRLP55, ATRLP56, ATRLP57

Pi-score	Log2-ratio	Fold-Change	p-value
		| 2 |	0.01

no results for current filter criteria

created with GENEVESTIGATOR

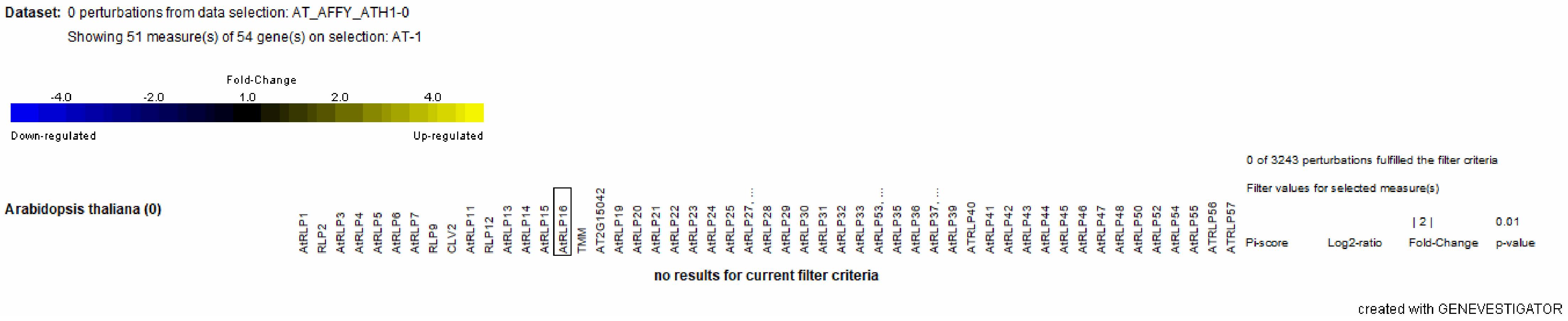

Dataset: 0 perturbations from data selection: AT_AFFY_ATH1-0
Showing 51 measure(s) of 54 gene(s) on selection: AT-1
Fold-Change
-4.0
-2.0
1.0
2.0
4.0
Down-regulated
Up-regulated
0 of 3243 perturbations fulfilled the filter criteria
Filter values for selected measure(s)
Arabidopsis thaliana (0)
AtRLP1
RLP2
AtRLP3
AtRLP4
AtRLP5
AtRLP6
AtRLP7
RLP9
CLV2
AtRLP11
RLP12
AtRLP13
AtRLP14
AtRLP15
AtRLP16
TMM
AT2G15042
AtRLP19
AtRLP20
AtRLP21
AtRLP22
AtRLP23
AtRLP24
AtRLP25
AtRLP27, ...
AtRLP28
AtRLP29
AtRLP30
AtRLP31
AtRLP32
AtRLP33
AtRLP53, ...
AtRLP35
AtRLP36
AtRLP37, ...
AtRLP39
ATRLP40
AtRLP41
AtRLP42
AtRLP43
AtRLP44
AtRLP45
AtRLP46
AtRLP47
AtRLP48
AtRLP50
AtRLP52
AtRLP54
AtRLP55
ATRLP56
ATRLP57
Pi-score
Log2-ratio
| 2 |
Fold-Change
0.01
p-value
no results for current filter criteria
created with GENEVESTIGATOR

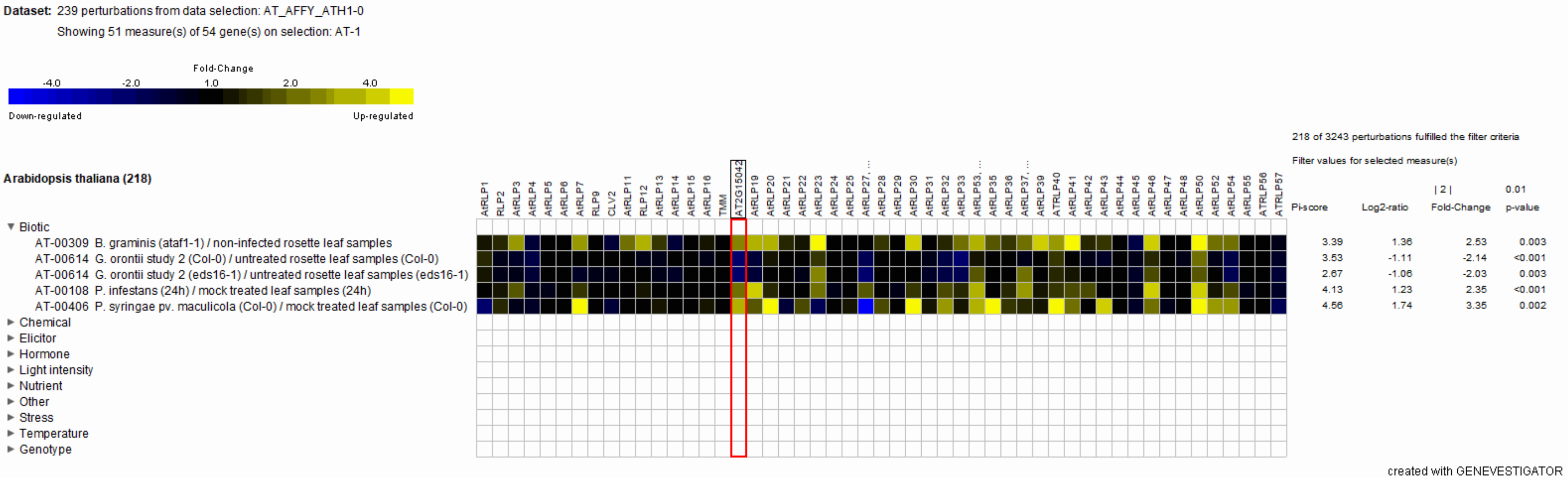
Dataset: 239 perturbations from data selection: AT_AFFY_ATH1-0
Showing 51 measure(s) of 54 gene(s) on selection: AT-1
Fold-Change
-4.0
-2.0
1.0
2.0
4.0
Down-regulated
Up-regulated
218 of 3243 perturbations fulfilled the filter criteria
Filter values for selected measure(s)
Arabidopsis thaliana (218)
AtRLP1
RLP2
AtRLP3
AtRLP4
AtRLP5
AtRLP6
AtRLP7
RLP9
CLV2
AtRLP11
RLP12
AtRLP13
AtRLP14
AtRLP15
AtRLP16
TMM
AT2G15042
AtRLP19
AtRLP20
AtRLP21
AtRLP22
AtRLP23
AtRLP24
AtRLP25
AtRLP27, ...
AtRLP28
AtRLP29
AtRLP30
AtRLP31
AtRLP32
AtRLP33
AtRLP53, ...
AtRLP35
AtRLP36
AtRLP37, ...
AtRLP39
ATRLP40
AtRLP41
AtRLP42
AtRLP43
AtRLP44
AtRLP45
AtRLP46
AtRLP47
AtRLP48
AtRLP50
AtRLP52
AtRLP54
AtRLP55
ATRLP56
ATRLP57
| 2 |
0.01
Pi-score
Log2-ratio
Fold-Change
p-value
Biotic
AT-00309 B. graminis (ataf1-1) / non-infected rosette leaf samples 3.39 1.36 2.53 0.003
AT-00614 G. orontii study 2 (Col-0) / untreated rosette leaf samples (Col-0) 3.53 -1.11 -2.14 <0.001
AT-00614 G. orontii study 2 (eds16-1) / untreated rosette leaf samples (eds16-1) 2.67 -1.06 -2.03 0.003
AT-00108 P. infestans (24h) / mock treated leaf samples (24h) 4.13 1.23 2.35 <0.001
AT-00406 P. syringae pv. maculicola (Col-0) / mock treated leaf samples (Col-0) 4.56 1.74 3.35 0.002
Chemical
Elicitor
Hormone
Light intensity
Nutrient
Other
Stress
Temperature
Genotype
created with GENEVESTIGATOR

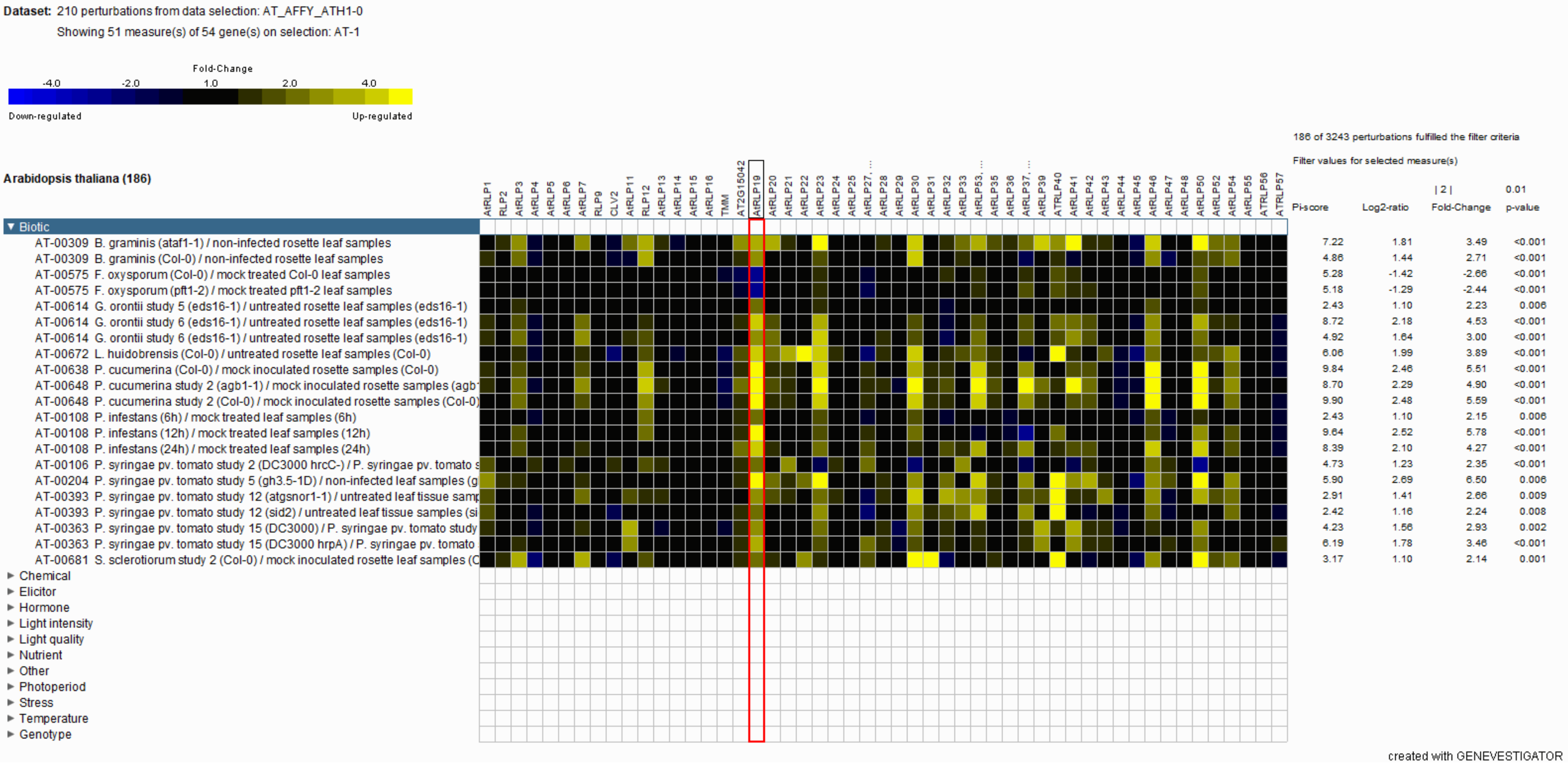
Dataset: 210 perturbations from data selection: AT_AFFY_ATH1-0
Showing 51 measure(s) of 54 gene(s) on selection: AT-1
Fold-Change
-4.0
-2.0
1.0
2.0
4.0
Down-regulated
Up-regulated
186 of 3243 perturbations fulfilled the filter criteria
Filter values for selected measure(s)
Arabidopsis thaliana (186)
AtRLP1
RLP2
AtRLP3
AtRLP4
AtRLP5
AtRLP6
AtRLP7
RLP9
CLV2
AtRLP11
RLP12
AtRLP13
AtRLP14
AtRLP15
AtRLP16
TMM
AT2G15042
AtRLP19
AtRLP20
AtRLP21
AtRLP22
AtRLP23
AtRLP24
AtRLP25
AtRLP27, ...
AtRLP28
AtRLP29
AtRLP30
AtRLP31
AtRLP32
AtRLP33
AtRLP53, ...
AtRLP35
AtRLP36
AtRLP37, ...
AtRLP39
ATRLP40
AtRLP41
AtRLP42
AtRLP43
AtRLP44
AtRLP45
AtRLP46
AtRLP47
AtRLP48
AtRLP50
AtRLP52
AtRLP54
AtRLP55
ATRLP56
ATRLP57
| 2 |
0.01
Pi-score
Log2-ratio
Fold-Change
p-value
Biotic
AT-00309 B. graminis (ataf1-1) / non-infected rosette leaf samples 7.22 1.81 3.49 <0.001
AT-00309 B. graminis (Col-0) / non-infected rosette leaf samples 4.86 1.44 2.71 <0.001
AT-00575 F. oxysporum (Col-0) / mock treated Col-0 leaf samples 5.28 -1.42 -2.66 <0.001
AT-00575 F. oxysporum (pft1-2) / mock treated pft1-2 leaf samples 5.18 -1.29 -2.44 <0.001
AT-00614 G. orontii study 5 (eds16-1) / untreated rosette leaf samples (eds16-1) 2.43 1.10 2.23 0.006
AT-00614 G. orontii study 6 (eds16-1) / untreated rosette leaf samples (eds16-1) 8.72 2.18 4.53 <0.001
AT-00614 G. orontii study 6 (eds16-1) / untreated rosette leaf samples (eds16-1) 4.92 1.64 3.00 <0.001
AT-00672 L. huidobrensis (Col-0) / untreated rosette leaf samples (Col-0) 6.06 1.99 3.89 <0.001
AT-00638 P. cucumerina (Col-0) / mock inoculated rosette samples (Col-0) 9.84 2.46 5.51 <0.001
AT-00648 P. cucumerina study 2 (agb1-1) / mock inoculated rosette samples (agb1 8.70 2.29 4.90 <0.001
AT-00648 P. cucumerina study 2 (Col-0) / mock inoculated rosette samples (Col-0) 9.90 2.48 5.59 <0.001
AT-00108 P. infestans (6h) / mock treated leaf samples (6h) 2.43 1.10 2.15 0.006
AT-00108 P. infestans (12h) / mock treated leaf samples (12h) 9.64 2.52 5.78 <0.001
AT-00108 P. infestans (24h) / mock treated leaf samples (24h) 8.39 2.10 4.27 <0.001
AT-00106 P. syringae pv. tomato study 2 (DC3000 hrcC-) / P. syringae pv. tomato s 4.73 1.23 2.35 <0.001
AT-00204 P. syringae pv. tomato study 5 (gh3.5-1D) / non-infected leaf samples (g 5.90 2.69 6.50 0.006
AT-00393 P. syringae pv. tomato study 12 (atgsnor1-1) / untreated leaf tissue samp 2.91 1.41 2.66 0.009
AT-00393 P. syringae pv. tomato study 12 (sid2) / untreated leaf tissue samples (si 2.42 1.16 2.24 0.008
AT-00363 P. syringae pv. tomato study 15 (DC3000) / P. syringae pv. tomato study 4.23 1.56 2.93 0.002
AT-00363 P. syringae pv. tomato study 15 (DC3000 hrpA) / P. syringae pv. tomato 6.19 1.78 3.46 <0.001
AT-00681 S. sclerotiorum study 2 (Col-0) / mock inoculated rosette leaf samples (C 3.17 1.10 2.14 0.001
Chemical
Elicitor
Hormone
Light intensity
Light quality
Nutrient
Other
Photoperiod
Stress
Temperature
Genotype
created with GENEVESTIGATOR

Dataset: 105 perturbations from data selection: AT_AFFY_ATH1-0

Showing 51 measure(s) of 54 gene(s) on selection: AT-1

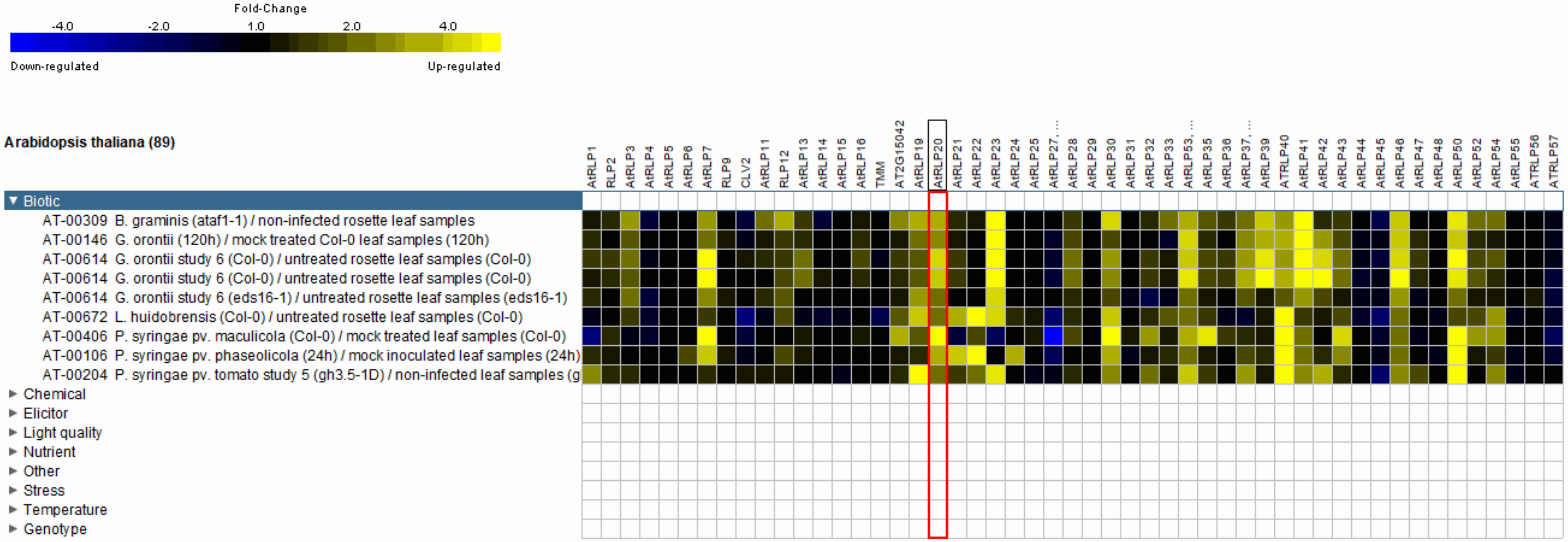

89 of 3243 perturbations fulfilled the filter criteria

Filter values for selected measure(s)

	Pi-score	Log2-ratio	Fold-Change	p-value
			| 2 |	0.01
AT-00309	4.99	1.80	3.54	0.002
AT-00146	2.79	1.29	2.52	0.007
AT-00614	8.25	2.06	4.24	<0.001
AT-00614	8.20	2.05	4.20	<0.001
AT-00614	3.48	1.01	2.03	<0.001
AT-00672	2.16	1.01	2.03	0.007
AT-00406	11.03	3.54	11.25	<0.001
AT-00106	6.15	1.64	3.11	<0.001
AT-00204	3.00	1.13	2.20	0.002

created with GENEVESTIGATOR

Dataset: 96 perturbations from data selection: AT_AFFY_ATH1-0

Showing 51 measure(s) of 54 gene(s) on selection: AT-1

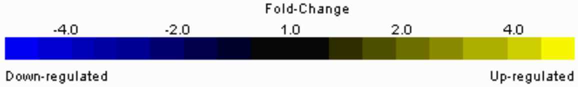

79 of 3243 perturbations fulfilled the filter criteria

Filter values for selected measure(s)

Arabidopsis thaliana (79)

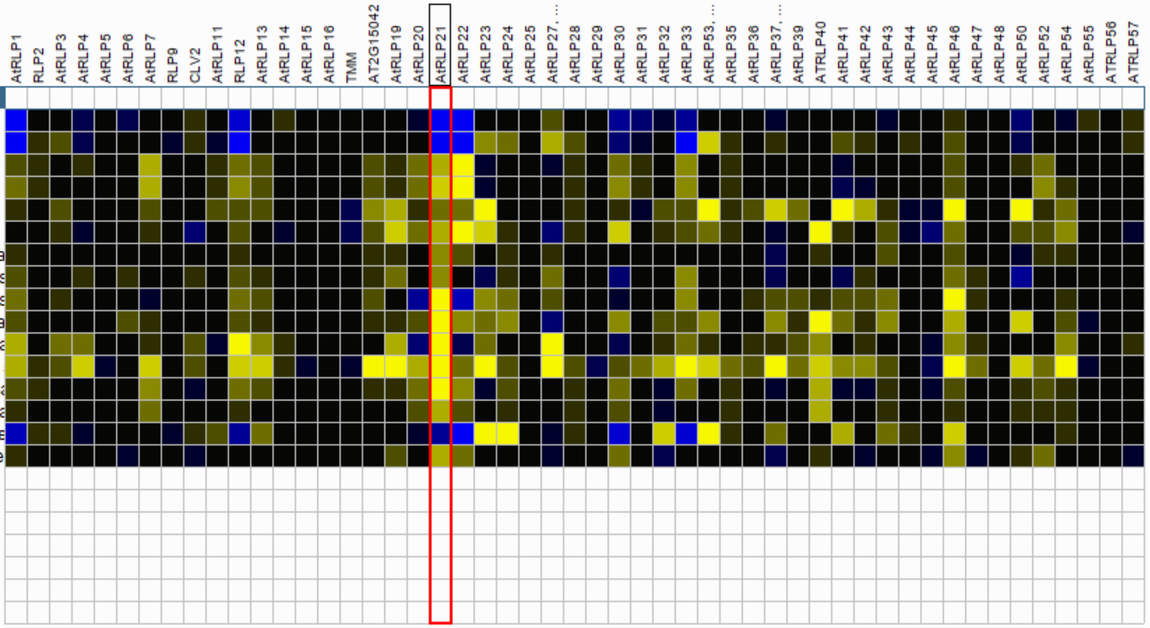

	Pi-score	Log2-ratio	Fold-Change	p-value
			| 2 |	0.01
▼ Biotic				
AT-00391 A. brassicicola (Ler) / untreated leaf disc samples (Ler)	13.37	-3.67	-13.15	<0.001
AT-00391 A. brassicicola (penta) / untreated leaf disc samples (penta)	12.86	-3.21	-9.30	<0.001
AT-00202 E. coli (O157:H7) / mock inoculated leaf samples	4.67	1.80	3.65	0.003
AT-00202 E. coli (TUV86-2 fliC) / mock inoculated leaf samples	5.29	1.88	3.82	0.002
AT-00553 H. arabidopsidis study 5 (rpp4) / untreated seedling samples (rpp4)	3.24	1.21	2.31	0.002
AT-00672 L. huidobrensis (Col-0) / untreated rosette leaf samples (Col-0)	5.25	1.68	3.23	<0.001
AT-00106 P. syringae pv. tomato study 2 (DC3000 hrcC-) / mock inoculated leaf sa	4.68	1.30	2.47	<0.001
AT-00106 P. syringae pv. tomato study 2 (DC3000 hrcC-) / P. syringae pv. tomato s	5.65	1.41	2.67	<0.001
AT-00106 P. syringae pv. tomato study 3 (DC3000 hrcC-) / P. syringae pv. tomato s	13.76	3.44	11.05	<0.001
AT-00106 P. syringae pv. tomato study 3 (DC3000 hrcC-) / mock inoculated leaf sa	10.44	2.90	7.57	<0.001
AT-00202 P. syringae pv. tomato study 9 (DC3118 Cor-hrpS) / P. syringae pv. toma	7.55	2.28	4.95	<0.001
AT-00202 P. syringae pv. tomato study 10 (DC3000 hrpA) / P. syringae pv. tomato	7.20	3.20	9.67	0.006
AT-00202 P. syringae pv. tomato study 10 (DC3000 hrpA) / mock inoculated leaf sa	8.16	2.49	5.85	<0.001
AT-00202 P. syringae pv. tomato study 10 (DC3000 hrpAfliC) / mock inoculated lea	5.84	1.88	3.77	<0.001
AT-00391 P. syringae pv. tomato study 11 (penta) / untreated leaf disc samples (pe	4.73	-1.61	-3.02	0.001
AT-00582 P. syringae pv. tomato study 18 (DC3000 ΔhrcC) / mock inoculated rose	3.98	1.85	3.70	0.007
▶ Chemical				
▶ Elicitor				
▶ Hormone				
▶ Light intensity				
▶ Photoperiod				
▶ Stress				
▶ Genotype				

created with GENEVESTIGATOR

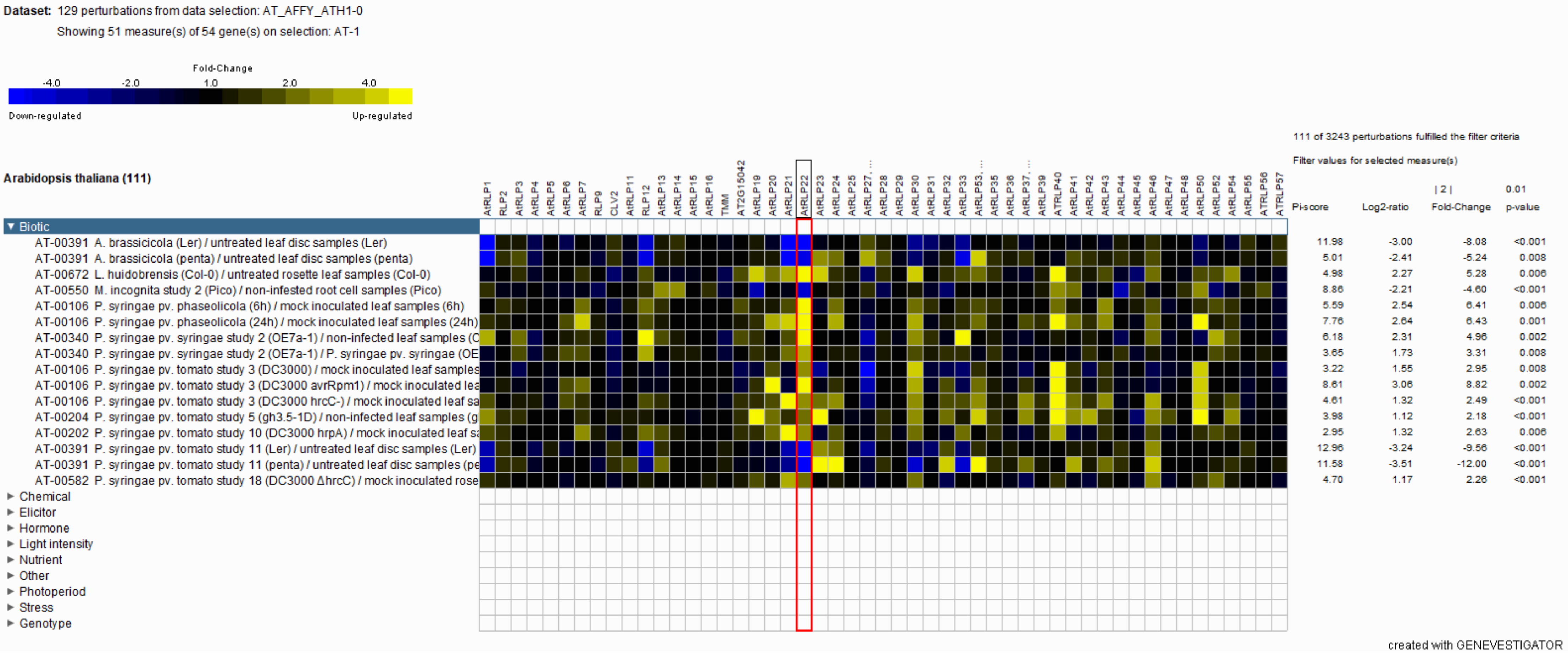

Dataset: 129 perturbations from data selection: AT_AFFY_ATH1-0
Showing 51 measure(s) of 54 gene(s) on selection: AT-1
Fold-Change
-4.0 -2.0 1.0 2.0 4.0
Down-regulated
Up-regulated
111 of 3243 perturbations fulfilled the filter criteria
Filter values for selected measure(s)
Arabidopsis thaliana (111)
AtRLP1 RLP2 AtRLP3 AtRLP4 AtRLP5 AtRLP6 AtRLP7 RLP9 CLV2 AtRLP11 RLP12 AtRLP13 AtRLP14 AtRLP15 AtRLP16 TMM AT2G15042 AtRLP19 AtRLP20 AtRLP21 AtRLP22 AtRLP23 AtRLP24 AtRLP25 AtRLP27, ... AtRLP28 AtRLP29 AtRLP30 AtRLP31 AtRLP32 AtRLP33 AtRLP53, ... AtRLP35 AtRLP36 AtRLP37, ... AtRLP39 ATRLP40 AtRLP41 AtRLP42 AtRLP43 AtRLP44 AtRLP45 AtRLP46 AtRLP47 AtRLP48 AtRLP50 AtRLP52 AtRLP54 AtRLP55 ATRLP56 ATRLP57
Pi-score Log2-ratio |2| Fold-Change 0.01 p-value
▼ Biotic
AT-00391 A. brassicicola (Ler) / untreated leaf disc samples (Ler) 11.98 -3.00 -8.08 <0.001
AT-00391 A. brassicicola (penta) / untreated leaf disc samples (penta) 5.01 -2.41 -5.24 0.008
AT-00672 L. huidobrensis (Col-0) / untreated rosette leaf samples (Col-0) 4.98 2.27 5.28 0.006
AT-00550 M. incognita study 2 (Pico) / non-infested root cell samples (Pico) 8.86 -2.21 -4.60 <0.001
AT-00106 P. syringae pv. phaseolicola (6h) / mock inoculated leaf samples (6h) 5.59 2.54 6.41 0.006
AT-00106 P. syringae pv. phaseolicola (24h) / mock inoculated leaf samples (24h) 7.76 2.64 6.43 0.001
AT-00340 P. syringae pv. syringae study 2 (OE7a-1) / non-infected leaf samples (C 6.18 2.31 4.96 0.002
AT-00340 P. syringae pv. syringae study 2 (OE7a-1) / P. syringae pv. syringae (OE 3.65 1.73 3.31 0.008
AT-00106 P. syringae pv. tomato study 3 (DC3000) / mock inoculated leaf samples 3.22 1.55 2.95 0.008
AT-00106 P. syringae pv. tomato study 3 (DC3000 avrRpm1) / mock inoculated lea 8.61 3.06 8.82 0.002
AT-00106 P. syringae pv. tomato study 3 (DC3000 hrcC-) / mock inoculated leaf sa 4.61 1.32 2.49 <0.001
AT-00204 P. syringae pv. tomato study 5 (gh3.5-1D) / non-infected leaf samples (g 3.98 1.12 2.18 <0.001
AT-00202 P. syringae pv. tomato study 10 (DC3000 hrpA) / mock inoculated leaf s 2.95 1.32 2.63 0.006
AT-00391 P. syringae pv. tomato study 11 (Ler) / untreated leaf disc samples (Ler) 12.96 -3.24 -9.56 <0.001
AT-00391 P. syringae pv. tomato study 11 (penta) / untreated leaf disc samples (pe 11.58 -3.51 -12.00 <0.001
AT-00582 P. syringae pv. tomato study 18 (DC3000 ΔhrcC) / mock inoculated rose 4.70 1.17 2.26 <0.001
▶ Chemical
▶ Elicitor
▶ Hormone
▶ Light intensity
▶ Nutrient
▶ Other
▶ Photoperiod
▶ Stress
▶ Genotype
created with GENEVESTIGATOR

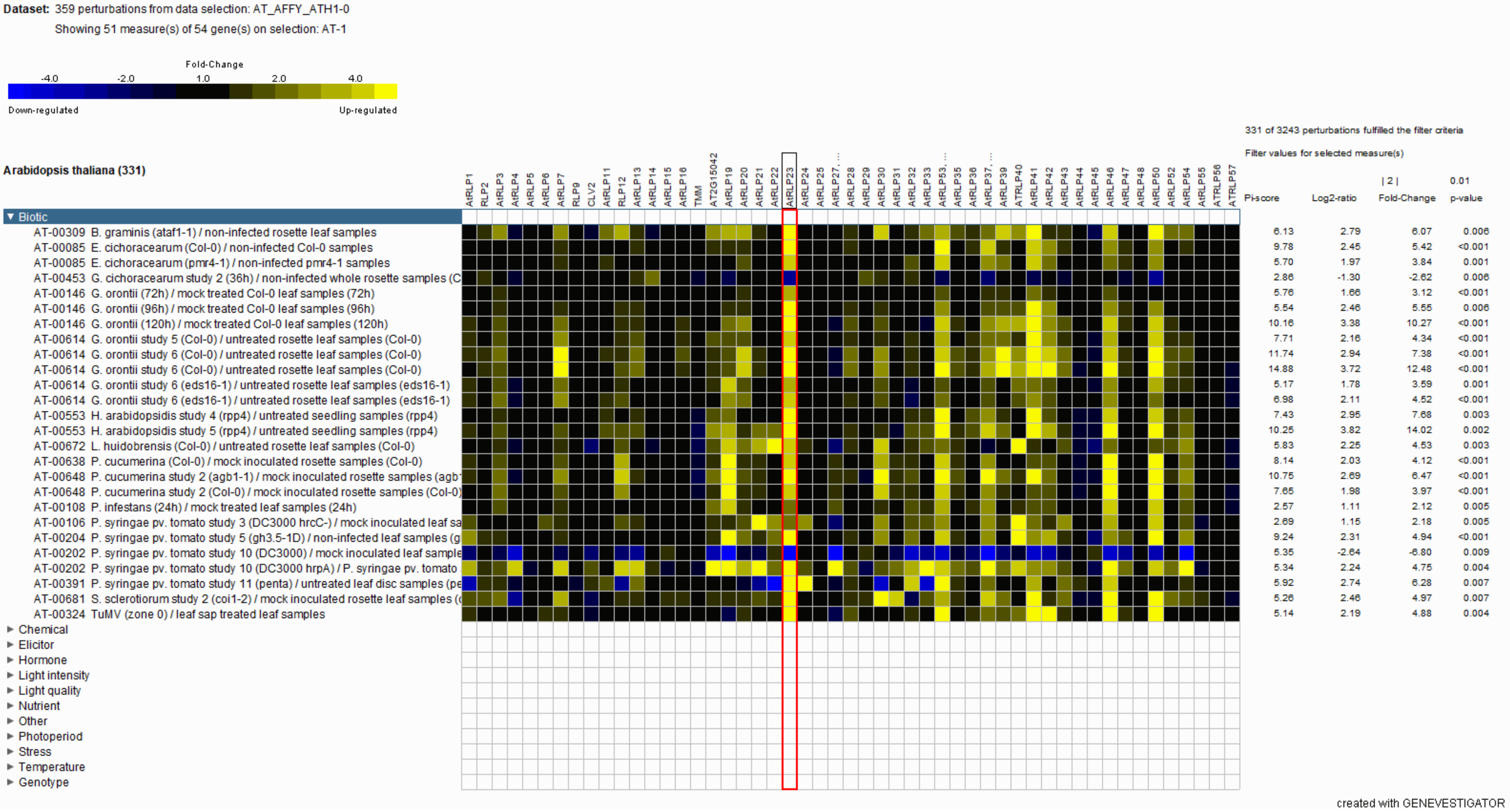

Dataset: 359 perturbations from data selection: AT_AFFY_ATH1-0
Showing 51 measure(s) of 54 gene(s) on selection: AT-1
Fold-Change
-4.0 -2.0 1.0 2.0 4.0
Down-regulated
Up-regulated
331 of 3243 perturbations fulfilled the filter criteria
Filter values for selected measure(s)
Arabidopsis thaliana (331)
AtRLP1 RLP2 AtRLP3 AtRLP4 AtRLP5 AtRLP6 AtRLP7 RLP9 CLV2 AtRLP11 RLP12 AtRLP13 AtRLP14 AtRLP15 AtRLP16 TMM AT2G15042 AtRLP19 AtRLP20 AtRLP21 AtRLP22 AtRLP23 AtRLP24 AtRLP25 AtRLP27, ... AtRLP28 AtRLP29 AtRLP30 AtRLP31 AtRLP32 AtRLP33 AtRLP53, ... AtRLP35 AtRLP36 AtRLP37, ... AtRLP39 ATRLP40 AtRLP41 AtRLP42 AtRLP43 AtRLP44 AtRLP45 AtRLP46 AtRLP47 AtRLP48 AtRLP50 AtRLP52 AtRLP54 AtRLP55 ATRLP56 ATRLP57
|2| 0.01
Pi-score Log2-ratio Fold-Change p-value
Biotic
AT-00309 B. graminis (ataf1-1) / non-infected rosette leaf samples 6.13 2.79 6.07 0.006
AT-00085 E. cichoracearum (Col-0) / non-infected Col-0 samples 9.78 2.45 5.42 <0.001
AT-00085 E. cichoracearum (pmr4-1) / non-infected pmr4-1 samples 5.70 1.97 3.84 0.001
AT-00453 G. cichoracearum study 2 (36h) / non-infected whole rosette samples (C 2.86 -1.30 -2.62 0.006
AT-00146 G. orontii (72h) / mock treated Col-0 leaf samples (72h) 5.76 1.66 3.12 <0.001
AT-00146 G. orontii (96h) / mock treated Col-0 leaf samples (96h) 5.54 2.46 5.55 0.006
AT-00146 G. orontii (120h) / mock treated Col-0 leaf samples (120h) 10.16 3.38 10.27 <0.001
AT-00614 G. orontii study 5 (Col-0) / untreated rosette leaf samples (Col-0) 7.71 2.16 4.34 <0.001
AT-00614 G. orontii study 6 (Col-0) / untreated rosette leaf samples (Col-0) 11.74 2.94 7.38 <0.001
AT-00614 G. orontii study 6 (Col-0) / untreated rosette leaf samples (Col-0) 14.88 3.72 12.48 <0.001
AT-00614 G. orontii study 6 (eds16-1) / untreated rosette leaf samples (eds16-1) 5.17 1.78 3.59 0.001
AT-00614 G. orontii study 6 (eds16-1) / untreated rosette leaf samples (eds16-1) 6.98 2.11 4.52 <0.001
AT-00553 H. arabidopsidis study 4 (rpp4) / untreated seedling samples (rpp4) 7.43 2.95 7.68 0.003
AT-00553 H. arabidopsidis study 5 (rpp4) / untreated seedling samples (rpp4) 10.25 3.82 14.02 0.002
AT-00672 L. huidobrensis (Col-0) / untreated rosette leaf samples (Col-0) 5.83 2.25 4.53 0.003
AT-00638 P. cucumerina (Col-0) / mock inoculated rosette samples (Col-0) 8.14 2.03 4.12 <0.001
AT-00648 P. cucumerina study 2 (agb1-1) / mock inoculated rosette samples (agb1 10.75 2.69 6.47 <0.001
AT-00648 P. cucumerina study 2 (Col-0) / mock inoculated rosette samples (Col-0) 7.65 1.98 3.97 <0.001
AT-00108 P. infestans (24h) / mock treated leaf samples (24h) 2.57 1.11 2.12 0.005
AT-00106 P. syringae pv. tomato study 3 (DC3000 hrcC-) / mock inoculated leaf sa 2.69 1.15 2.18 0.005
AT-00204 P. syringae pv. tomato study 5 (gh3.5-1D) / non-infected leaf samples (g 9.24 2.31 4.94 <0.001
AT-00202 P. syringae pv. tomato study 10 (DC3000) / mock inoculated leaf sample 5.35 -2.64 -6.80 0.009
AT-00202 P. syringae pv. tomato study 10 (DC3000 hrpA) / P. syringae pv. tomato 5.34 2.24 4.75 0.004
AT-00391 P. syringae pv. tomato study 11 (penta) / untreated leaf disc samples (pe 5.92 2.74 6.28 0.007
AT-00681 S. sclerotiorum study 2 (coi1-2) / mock inoculated rosette leaf samples (5.26 2.46 4.97 0.007
AT-00324 TuMV (zone 0) / leaf sap treated leaf samples 5.14 2.19 4.88 0.004
Chemical
Elicitor
Hormone
Light intensity
Light quality
Nutrient
Other
Photoperiod
Stress
Temperature
Genotype
created with GENEVESTIGATOR

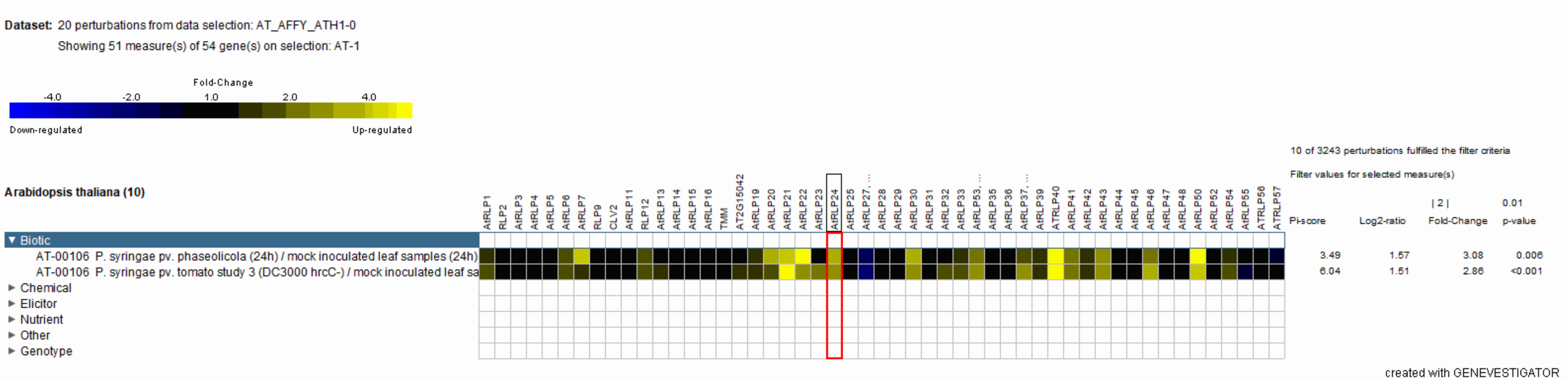

Dataset: 20 perturbations from data selection: AT_AFFY_ATH1-0
Showing 51 measure(s) of 54 gene(s) on selection: AT-1
Fold-Change
-4.0
-2.0
1.0
2.0
4.0
Down-regulated
Up-regulated
Arabidopsis thaliana (10)
AtRLP1
RLP2
AtRLP3
AtRLP4
AtRLP5
AtRLP6
AtRLP7
RLP9
CLV2
AtRLP11
RLP12
AtRLP13
AtRLP14
AtRLP15
AtRLP16
TMM
AT2G15042
AtRLP19
AtRLP20
AtRLP21
AtRLP22
AtRLP23
AtRLP24
AtRLP25
AtRLP27, ...
AtRLP28
AtRLP29
AtRLP30
AtRLP31
AtRLP32
AtRLP33
AtRLP53, ...
AtRLP35
AtRLP36
AtRLP37, ...
AtRLP39
ATRLP40
AtRLP41
AtRLP42
AtRLP43
AtRLP44
AtRLP45
AtRLP46
AtRLP47
AtRLP48
AtRLP50
AtRLP52
AtRLP54
AtRLP55
ATRLP56
ATRLP57
Biotic
AT-00106 P. syringae pv. phaseolicola (24h) / mock inoculated leaf samples (24h)
AT-00106 P. syringae pv. tomato study 3 (DC3000 hrcC-) / mock inoculated leaf sa
Chemical
Elicitor
Nutrient
Other
Genotype
10 of 3243 perturbations fulfilled the filter criteria
Filter values for selected measure(s)
| 2 |
0.01
Pi-score
Log2-ratio
Fold-Change
p-value
3.49
1.57
3.08
0.006
6.04
1.51
2.86
<0.001
created with GENEVESTIGATOR

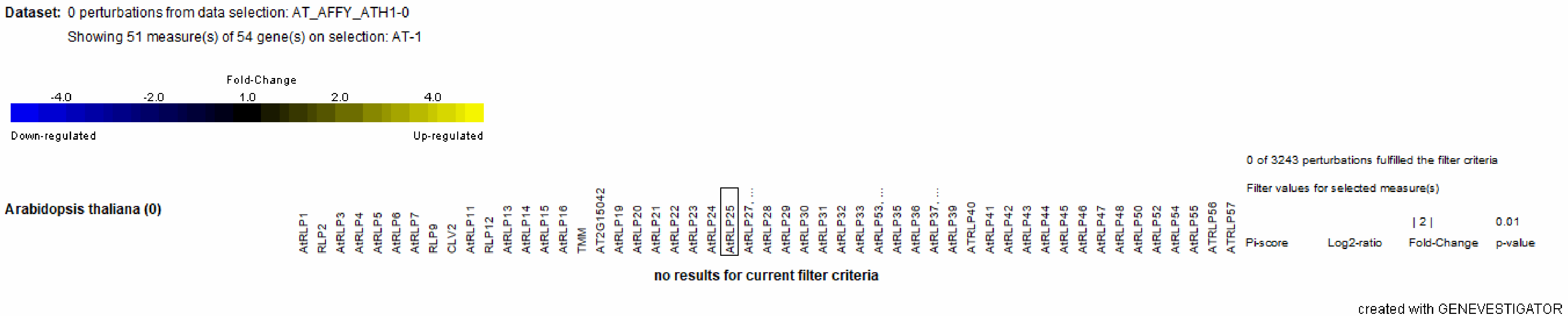

Dataset: 0 perturbations from data selection: AT_AFFY_ATH1-0
Showing 51 measure(s) of 54 gene(s) on selection: AT-1
Fold-Change
-4.0
-2.0
1.0
2.0
4.0
Down-regulated
Up-regulated
0 of 3243 perturbations fulfilled the filter criteria
Filter values for selected measure(s)
Arabidopsis thaliana (0)
AtRLP1
RLP2
AtRLP3
AtRLP4
AtRLP5
AtRLP6
AtRLP7
RLP9
CLV2
AtRLP11
RLP12
AtRLP13
AtRLP14
AtRLP15
AtRLP16
TMM
AT2G15042
AtRLP19
AtRLP20
AtRLP21
AtRLP22
AtRLP23
AtRLP24
AtRLP25
AtRLP27, ...
AtRLP28
AtRLP29
AtRLP30
AtRLP31
AtRLP32
AtRLP33
AtRLP53, ...
AtRLP35
AtRLP36
AtRLP37, ...
AtRLP39
ATRLP40
AtRLP41
AtRLP42
AtRLP43
AtRLP44
AtRLP45
AtRLP46
AtRLP47
AtRLP48
AtRLP50
AtRLP52
AtRLP54
AtRLP55
ATRLP56
ATRLP57
| 2 |
0.01
Pi-score
Log2-ratio
Fold-Change
p-value
no results for current filter criteria
created with GENEVESTIGATOR

Dataset: 188 perturbations from data selection: AT_AFFY_ATH1-0

Showing 51 measure(s) of 54 gene(s) on selection: AT-1

Fold-Change

-4.0 -2.0 1.0 2.0 4.0

Down-regulated Up-regulated

165 of 3243 perturbations fulfilled the filter criteria

Filter values for selected measure(s)

Arabidopsis thaliana (165)

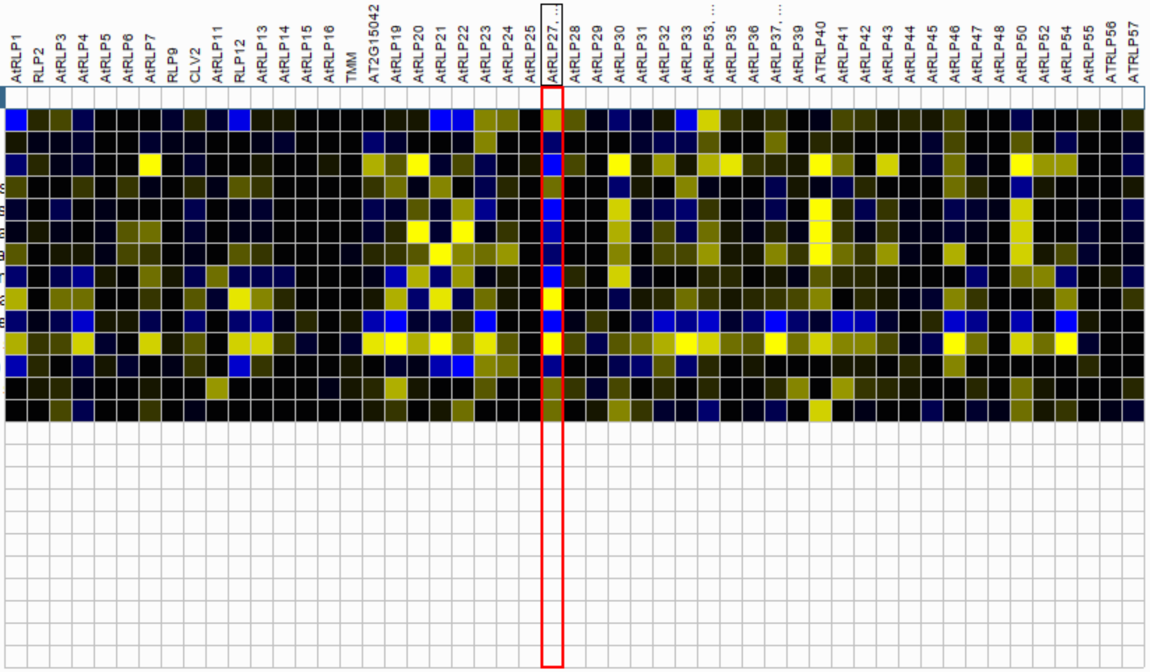

▼ Biotic	Pi-score	Log2-ratio	Fold-Change (| 2 |)	p-value (0.01)
AT-00391 A. brassicicola (penta) / untreated leaf disc samples (penta)	3.44	1.67	3.13	0.009
AT-00614 G. orontii study 2 (eds16-1) / untreated rosette leaf samples (eds16-1)	3.65	-1.18	-2.22	<0.001
AT-00406 P. syringae pv. maculicola (Col-0) / mock treated leaf samples (Col-0)	6.95	-2.57	-6.28	0.002
AT-00106 P. syringae pv. tomato study 2 (DC3000 hrcC-) / P. syringae pv. tomato s	4.19	1.19	2.27	<0.001
AT-00106 P. syringae pv. tomato study 3 (DC3000) / mock inoculated leaf samples	9.75	-2.44	-5.42	<0.001
AT-00106 P. syringae pv. tomato study 3 (DC3000 avrRpm1) / mock inoculated lea	4.59	-1.99	-3.74	0.005
AT-00106 P. syringae pv. tomato study 3 (DC3000 hrcC-) / mock inoculated leaf sa	4.29	-1.23	-2.33	<0.001
AT-00202 P. syringae pv. tomato study 9 (DC3118 Cor-) / mock inoculated leaf sar	6.40	-3.04	-8.34	0.008
AT-00202 P. syringae pv. tomato study 9 (DC3118 Cor-hrpS) / P. syringae pv. toma	6.68	2.53	5.73	0.002
AT-00202 P. syringae pv. tomato study 10 (DC3000) / mock inoculated leaf sample	13.83	-3.46	-11.12	<0.001
AT-00202 P. syringae pv. tomato study 10 (DC3000 hrpA) / P. syringae pv. tomato	7.81	3.16	9.29	0.003
AT-00391 P. syringae pv. tomato study 11 (Ler) / untreated leaf disc samples (Ler)	3.07	-1.44	-2.81	0.007
AT-00363 P. syringae pv. tomato study 15 (DC3000 hrpA) / P. syringae pv. tomato	2.67	1.14	2.19	0.005
AT-00681 S. sclerotiorum (Col-0) / mock inoculated rosette leaf samples (Col-0)	4.56	1.14	2.20	<0.001

- ▶ Chemical
- ▶ Elicitor
- ▶ Hormone
- ▶ Light intensity
- ▶ Light quality
- ▶ Nutrient
- ▶ Other
- ▶ Photoperiod
- ▶ Stress
- ▶ Temperature
- ▶ Genotype

created with GENEVESTIGATOR

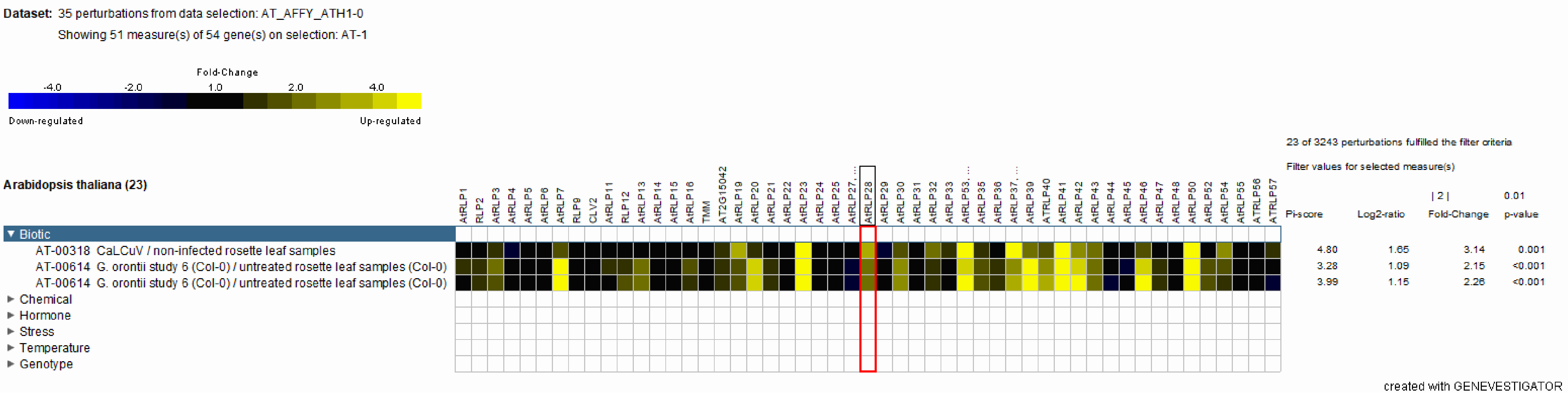
Dataset: 35 perturbations from data selection: AT_AFFY_ATH1-0
Showing 51 measure(s) of 54 gene(s) on selection: AT-1
Fold-Change
-4.0
-2.0
1.0
2.0
4.0
Down-regulated
Up-regulated
23 of 3243 perturbations fulfilled the filter criteria
Filter values for selected measure(s)
Arabidopsis thaliana (23)
AtRLP1 RLP2 AtRLP3 AtRLP4 AtRLP5 AtRLP6 AtRLP7 RLP9 CLV2 AtRLP11 RLP12 AtRLP13 AtRLP14 AtRLP15 AtRLP16 TMM AT2G15042 AtRLP19 AtRLP20 AtRLP21 AtRLP22 AtRLP23 AtRLP24 AtRLP25 AtRLP27, ... AtRLP28 AtRLP29 AtRLP30 AtRLP31 AtRLP32 AtRLP33 AtRLP53, ... AtRLP35 AtRLP36 AtRLP37, ... AtRLP39 ATRLP40 AtRLP41 AtRLP42 AtRLP43 AtRLP44 AtRLP45 AtRLP46 AtRLP47 AtRLP48 AtRLP50 AtRLP52 AtRLP54 AtRLP55 ATRLP56 ATRLP57
| 2 |
0.01
Pi-score
Log2-ratio
Fold-Change
p-value
Biotic
AT-00318 CaLCuV / non-infected rosette leaf samples
4.80
1.65
3.14
0.001
AT-00614 G. orontii study 6 (Col-0) / untreated rosette leaf samples (Col-0)
3.28
1.09
2.15
<0.001
AT-00614 G. orontii study 6 (Col-0) / untreated rosette leaf samples (Col-0)
3.99
1.15
2.26
<0.001
Chemical
Hormone
Stress
Temperature
Genotype
created with GENEVESTIGATOR

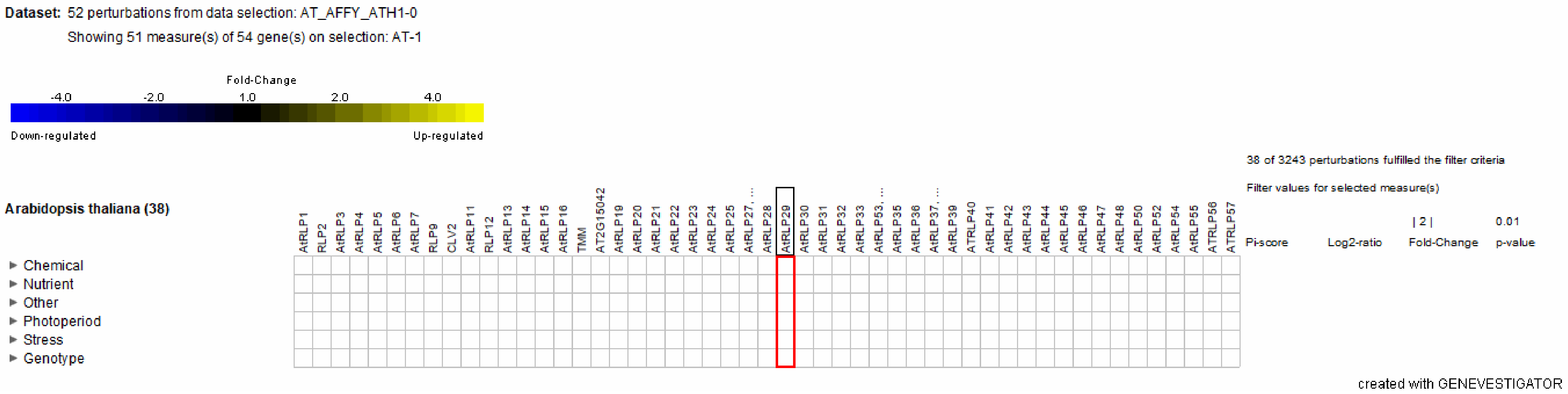

Dataset: 52 perturbations from data selection: AT_AFFY_ATH1-0
Showing 51 measure(s) of 54 gene(s) on selection: AT-1
Fold-Change
-4.0
-2.0
1.0
2.0
4.0
Down-regulated
Up-regulated
38 of 3243 perturbations fulfilled the filter criteria
Filter values for selected measure(s)
Arabidopsis thaliana (38)
AtRLP1
RLP2
AtRLP3
AtRLP4
AtRLP5
AtRLP6
AtRLP7
RLP9
CLV2
AtRLP11
RLP12
AtRLP13
AtRLP14
AtRLP15
AtRLP16
TMM
AT2G15042
AtRLP19
AtRLP20
AtRLP21
AtRLP22
AtRLP23
AtRLP24
AtRLP25
AtRLP27, ...
AtRLP28
AtRLP29
AtRLP30
AtRLP31
AtRLP32
AtRLP33
AtRLP53, ...
AtRLP35
AtRLP36
AtRLP37, ...
AtRLP39
ATRLP40
AtRLP41
AtRLP42
AtRLP43
AtRLP44
AtRLP45
AtRLP46
AtRLP47
AtRLP48
AtRLP50
AtRLP52
AtRLP54
AtRLP55
ATRLP56
ATRLP57
| 2 |
0.01
Pi-score
Log2-ratio
Fold-Change
p-value
Chemical
Nutrient
Other
Photoperiod
Stress
Genotype
created with GENEVESTIGATOR

Dataset: 251 perturbations from data selection: AT_AFFY_ATH1-0

Showing 51 measure(s) of 54 gene(s) on selection: AT-1

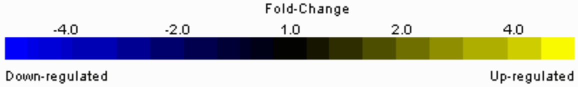

225 of 3243 perturbations fulfilled the filter criteria

Filter values for selected measure(s)

Arabidopsis thaliana (225)

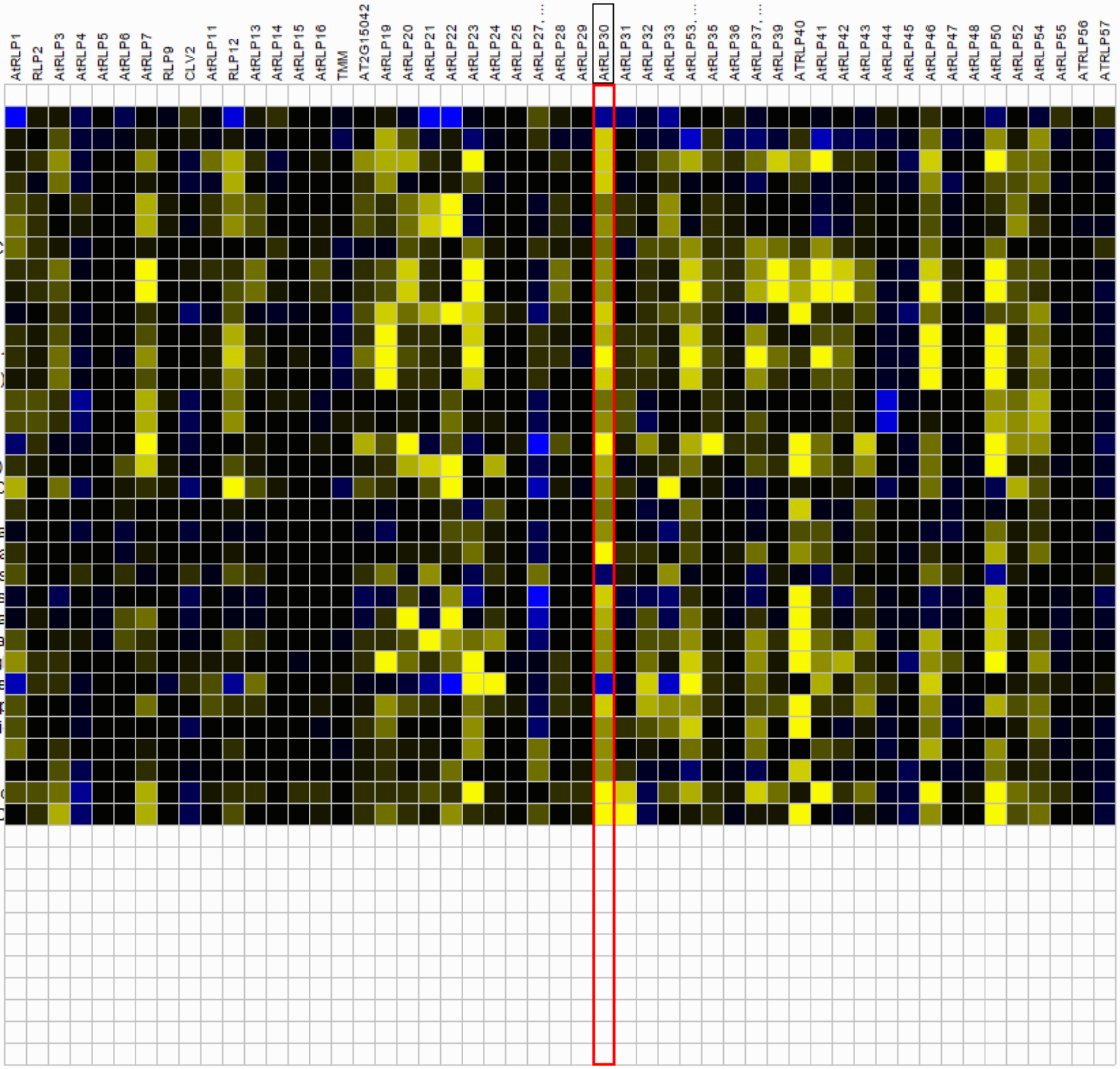

	Pi-score	Log2-ratio	| 2 | Fold-Change	0.01 p-value
▼ Biotic				
AT-00391 A. brassicicola (Ler) / untreated leaf disc samples (Ler)	3.19	-1.37	-2.57	0.005
AT-00661 A. brassicicola study 3 (Col-0) / mock treated leaf samples (Col-0)	8.06	2.02	4.04	<0.001
AT-00309 B. graminis (ataf1-1) / non-infected rosette leaf samples	7.48	2.21	4.72	<0.001
AT-00309 B. graminis (Col-0) / non-infected rosette leaf samples	8.44	2.11	4.35	<0.001
AT-00202 E. coli (O157:H7) / mock inoculated leaf samples	2.94	1.16	2.21	0.003
AT-00202 E. coli (TUV86-2 fliC) / mock inoculated leaf samples	3.55	1.34	2.51	0.002
AT-00453 G. cichoracearum study 2 (96h) / non-infected whole rosette samples (C	3.24	1.13	2.17	0.001
AT-00614 G. orontii study 6 (Col-0) / untreated rosette leaf samples (Col-0)	5.73	1.43	2.72	<0.001
AT-00614 G. orontii study 6 (Col-0) / untreated rosette leaf samples (Col-0)	4.50	1.30	2.45	<0.001
AT-00672 L. huidobrensis (Col-0) / untreated rosette leaf samples (Col-0)	5.90	2.00	4.12	0.001
AT-00638 P. cucumerina (Col-0) / mock inoculated rosette samples (Col-0)	4.51	1.82	3.64	0.003
AT-00648 P. cucumerina study 2 (agb1-1) / mock inoculated rosette samples (agb	11.17	2.83	7.20	<0.001
AT-00648 P. cucumerina study 2 (Col-0) / mock inoculated rosette samples (Col-0)	4.67	1.92	3.98	0.004
AT-00425 P. parasitica (6h) / non-infected root samples (Col-0)	3.17	1.07	2.09	0.001
AT-00425 P. parasitica (10.5h) / non-infected root samples (Col-0)	3.01	1.37	2.59	0.006
AT-00406 P. syringae pv. maculicola (Col-0) / mock treated leaf samples (Col-0)	12.88	3.68	12.37	<0.001
AT-00106 P. syringae pv. phaseolicola (24h) / mock inoculated leaf samples (24h)	7.07	1.77	3.43	<0.001
AT-00340 P. syringae pv. syringae study 2 (OE7a-1) / non-infected leaf samples (C	3.10	1.39	2.62	0.006
AT-00106 P. syringae pv. tomato (DC3000) / mock inoculated leaf samples (2h)	3.57	1.26	2.42	0.001
AT-00106 P. syringae pv. tomato study 2 (DC3000 avrRpm1) / mock inoculated lea	3.69	1.39	2.60	0.002
AT-00106 P. syringae pv. tomato study 2 (DC3000 avrRpm1) / P. syringae pv. toma	8.70	2.47	5.50	<0.001
AT-00106 P. syringae pv. tomato study 2 (DC3000 hrcC-) / P. syringae pv. tomato s	3.20	-1.19	-2.27	0.002
AT-00106 P. syringae pv. tomato study 3 (DC3000) / mock inoculated leaf samples	7.19	2.21	4.66	<0.001
AT-00106 P. syringae pv. tomato study 3 (DC3000 avrRpm1) / mock inoculated lea	4.74	1.78	3.51	0.002
AT-00106 P. syringae pv. tomato study 3 (DC3000 hrcC-) / mock inoculated leaf sa	4.93	1.42	2.69	<0.001
AT-00204 P. syringae pv. tomato study 5 (gh3.5-1D) / non-infected leaf samples (g	3.89	1.32	2.49	0.001
AT-00391 P. syringae pv. tomato study 11 (penta) / untreated leaf disc samples (pe	4.16	-1.92	-3.88	0.007
AT-00393 P. syringae pv. tomato study 12 (atgsnor1-1) / untreated leaf tissue samp	6.64	2.03	4.09	<0.001
AT-00393 P. syringae pv. tomato study 12 (sid2) / untreated leaf tissue samples (si	4.22	1.36	2.56	<0.001
AT-00585 R. solani (AG8) / mock inoculated whole plant samples	3.90	1.52	2.90	0.003
AT-00681 S. sclerotiorum (Col-0) / mock inoculated rosette leaf samples (Col-0)	2.63	1.25	2.45	0.008
AT-00681 S. sclerotiorum study 2 (coi1-2) / mock inoculated rosette leaf samples (	12.69	3.17	8.94	<0.001
AT-00681 S. sclerotiorum study 2 (Col-0) / mock inoculated rosette leaf samples (C	9.70	2.87	7.48	<0.001
▶ Chemical				
▶ Elicitor				
▶ Hormone				
▶ Light intensity				
▶ Light quality				
▶ Nutrient				
▶ Other				
▶ Photoperiod				
▶ Stress				
▶ Temperature				
▶ Genotype				

created with GENEVESTIGATOR

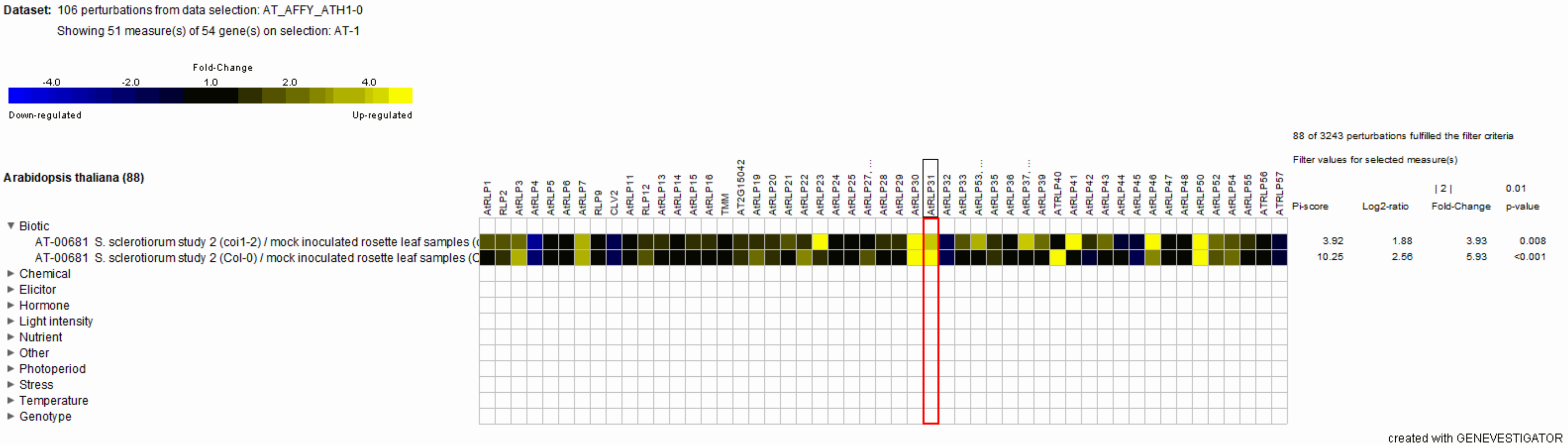

Dataset: 106 perturbations from data selection: AT_AFFY_ATH1-0
Showing 51 measure(s) of 54 gene(s) on selection: AT-1
Fold-Change
-4.0
-2.0
1.0
2.0
4.0
Down-regulated
Up-regulated
88 of 3243 perturbations fulfilled the filter criteria
Filter values for selected measure(s)
Arabidopsis thaliana (88)
AtRLP1
RLP2
AtRLP3
AtRLP4
AtRLP5
AtRLP6
AtRLP7
RLP9
CLV2
AtRLP11
RLP12
AtRLP13
AtRLP14
AtRLP15
AtRLP16
TMM
AT2G15042
AtRLP19
AtRLP20
AtRLP21
AtRLP22
AtRLP23
AtRLP24
AtRLP25
AtRLP27, ...
AtRLP28
AtRLP29
AtRLP30
AtRLP31
AtRLP32
AtRLP33
AtRLP53, ...
AtRLP35
AtRLP36
AtRLP37, ...
AtRLP39
ATRLP40
AtRLP41
AtRLP42
AtRLP43
AtRLP44
AtRLP45
AtRLP46
AtRLP47
AtRLP48
AtRLP50
AtRLP52
AtRLP54
AtRLP55
ATRLP56
ATRLP57
| 2 |
0.01
Pi-score
Log2-ratio
Fold-Change
p-value
Biotic
AT-00681 S. sclerotiorum study 2 (coi1-2) / mock inoculated rosette leaf samples (c
3.92
1.88
3.93
0.008
AT-00681 S. sclerotiorum study 2 (Col-0) / mock inoculated rosette leaf samples (C
10.25
2.56
5.93
<0.001
Chemical
Elicitor
Hormone
Light intensity
Nutrient
Other
Photoperiod
Stress
Temperature
Genotype
created with GENEVESTIGATOR

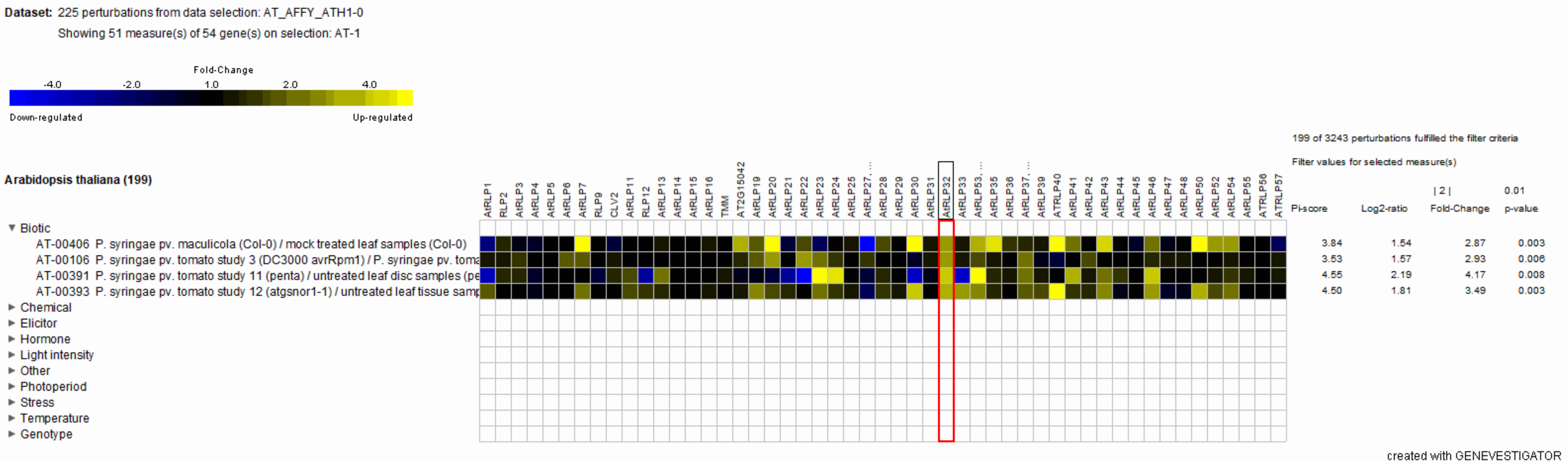

Dataset: 225 perturbations from data selection: AT_AFFY_ATH1-0
Showing 51 measure(s) of 54 gene(s) on selection: AT-1
Fold-Change
-4.0
-2.0
1.0
2.0
4.0
Down-regulated
Up-regulated
199 of 3243 perturbations fulfilled the filter criteria
Filter values for selected measure(s)
Arabidopsis thaliana (199)
AtRLP1
RLP2
AtRLP3
AtRLP4
AtRLP5
AtRLP6
AtRLP7
RLP9
CLV2
AtRLP11
RLP12
AtRLP13
AtRLP14
AtRLP15
AtRLP16
TMM
AT2G15042
AtRLP19
AtRLP20
AtRLP21
AtRLP22
AtRLP23
AtRLP24
AtRLP25
AtRLP27, ...
AtRLP28
AtRLP29
AtRLP30
AtRLP31
AtRLP32
AtRLP33
AtRLP53, ...
AtRLP35
AtRLP36
AtRLP37, ...
AtRLP39
ATRLP40
AtRLP41
AtRLP42
AtRLP43
AtRLP44
AtRLP45
AtRLP46
AtRLP47
AtRLP48
AtRLP50
AtRLP52
AtRLP54
AtRLP55
ATRLP56
ATRLP57
Pi-score
Log2-ratio
| 2 |
Fold-Change
0.01
p-value
Biotic
AT-00406 P. syringae pv. maculicola (Col-0) / mock treated leaf samples (Col-0)
AT-00106 P. syringae pv. tomato study 3 (DC3000 avrRpm1) / P. syringae pv. toma
AT-00391 P. syringae pv. tomato study 11 (penta) / untreated leaf disc samples (pe
AT-00393 P. syringae pv. tomato study 12 (atgsnor1-1) / untreated leaf tissue samp
3.84
1.54
2.87
0.003
3.53
1.57
2.93
0.006
4.55
2.19
4.17
0.008
4.50
1.81
3.49
0.003
Chemical
Elicitor
Hormone
Light intensity
Other
Photoperiod
Stress
Temperature
Genotype
created with GENEVESTIGATOR

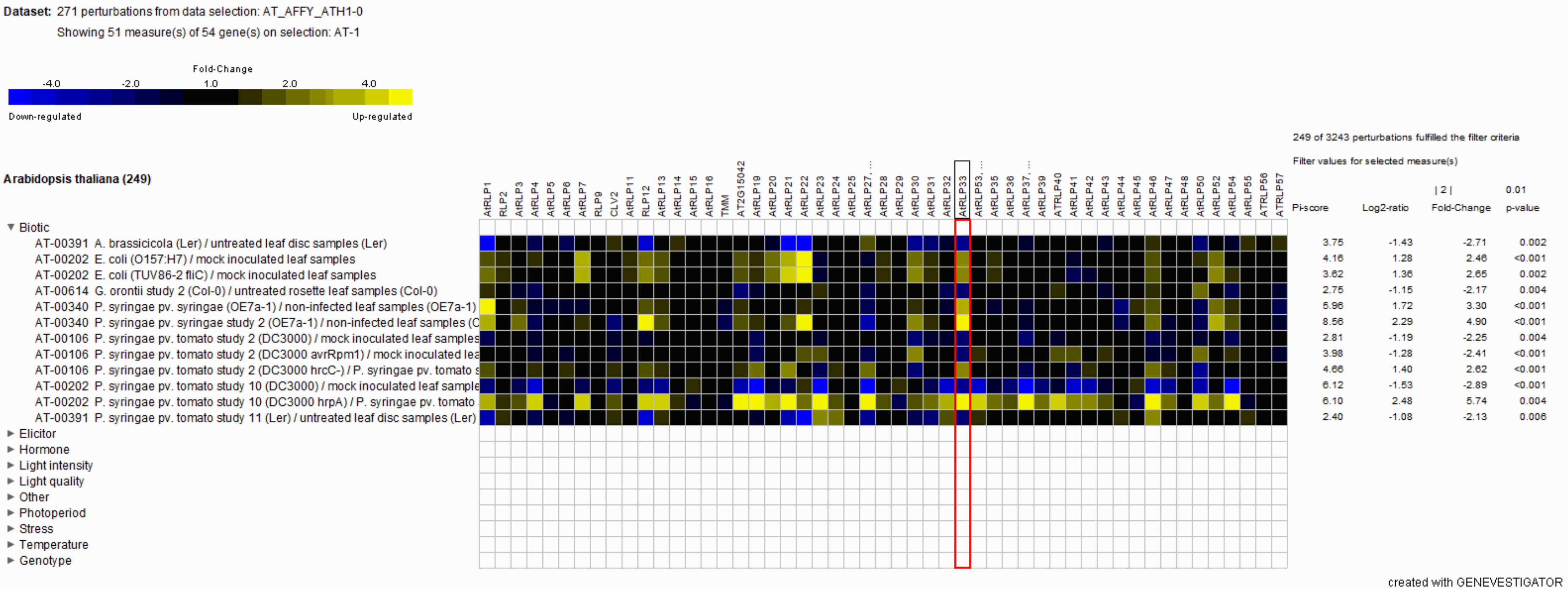
Dataset: 271 perturbations from data selection: AT_AFFY_ATH1-0
Showing 51 measure(s) of 54 gene(s) on selection: AT-1
Fold-Change
-4.0
-2.0
1.0
2.0
4.0
Down-regulated
Up-regulated
249 of 3243 perturbations fulfilled the filter criteria
Filter values for selected measure(s)
Arabidopsis thaliana (249)
AtRLP1
RLP2
AtRLP3
AtRLP4
AtRLP5
AtRLP6
AtRLP7
RLP9
CLV2
AtRLP11
RLP12
AtRLP13
AtRLP14
AtRLP15
AtRLP16
TMM
AT2G15042
AtRLP19
AtRLP20
AtRLP21
AtRLP22
AtRLP23
AtRLP24
AtRLP25
AtRLP27, ...
AtRLP28
AtRLP29
AtRLP30
AtRLP31
AtRLP32
AtRLP33
AtRLP53, ...
AtRLP35
AtRLP36
AtRLP37, ...
AtRLP39
ATRLP40
AtRLP41
AtRLP42
AtRLP43
AtRLP44
AtRLP45
AtRLP46
AtRLP47
AtRLP48
AtRLP50
AtRLP52
AtRLP54
AtRLP55
ATRLP56
ATRLP57
| 2 |
0.01
Pi-score
Log2-ratio
Fold-Change
p-value
Biotic
AT-00391 A. brassicicola (Ler) / untreated leaf disc samples (Ler) 3.75 -1.43 -2.71 0.002
AT-00202 E. coli (O157:H7) / mock inoculated leaf samples 4.16 1.28 2.46 <0.001
AT-00202 E. coli (TUV86-2 fliC) / mock inoculated leaf samples 3.62 1.36 2.65 0.002
AT-00614 G. orontii study 2 (Col-0) / untreated rosette leaf samples (Col-0) 2.75 -1.15 -2.17 0.004
AT-00340 P. syringae pv. syringae (OE7a-1) / non-infected leaf samples (OE7a-1) 5.96 1.72 3.30 <0.001
AT-00340 P. syringae pv. syringae study 2 (OE7a-1) / non-infected leaf samples (C 8.56 2.29 4.90 <0.001
AT-00106 P. syringae pv. tomato study 2 (DC3000) / mock inoculated leaf samples 2.81 -1.19 -2.25 0.004
AT-00106 P. syringae pv. tomato study 2 (DC3000 avrRpm1) / mock inoculated lea 3.98 -1.28 -2.41 <0.001
AT-00106 P. syringae pv. tomato study 2 (DC3000 hrcC-) / P. syringae pv. tomato s 4.66 1.40 2.62 <0.001
AT-00202 P. syringae pv. tomato study 10 (DC3000) / mock inoculated leaf sample 6.12 -1.53 -2.89 <0.001
AT-00202 P. syringae pv. tomato study 10 (DC3000 hrpA) / P. syringae pv. tomato 6.10 2.48 5.74 0.004
AT-00391 P. syringae pv. tomato study 11 (Ler) / untreated leaf disc samples (Ler) 2.40 -1.08 -2.13 0.006
Elicitor
Hormone
Light intensity
Light quality
Other
Photoperiod
Stress
Temperature
Genotype
created with GENEVESTIGATOR

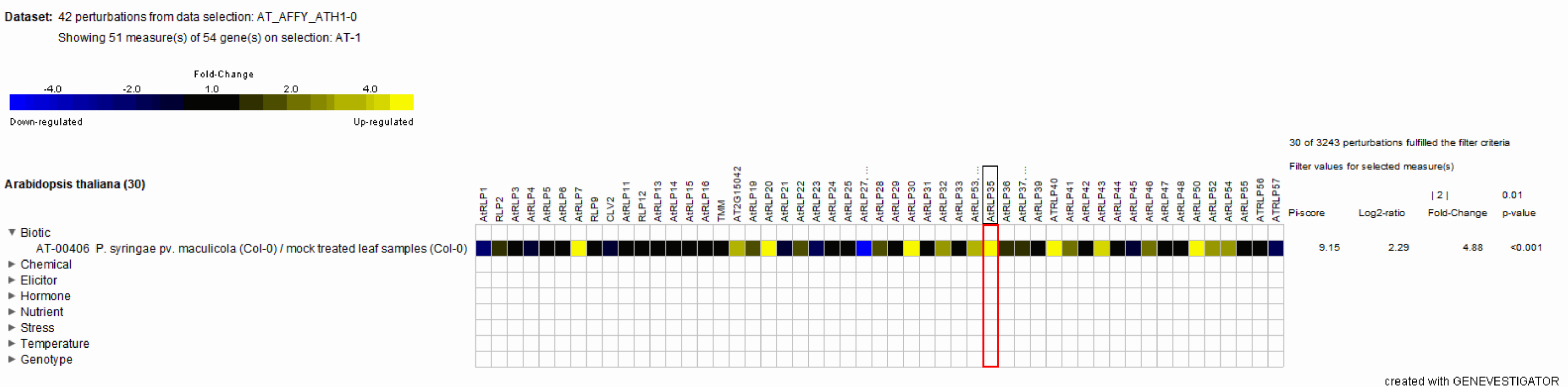
Dataset: 42 perturbations from data selection: AT_AFFY_ATH1-0
Showing 51 measure(s) of 54 gene(s) on selection: AT-1
Fold-Change
-4.0
-2.0
1.0
2.0
4.0
Down-regulated
Up-regulated
30 of 3243 perturbations fulfilled the filter criteria
Filter values for selected measure(s)
Arabidopsis thaliana (30)
AtRLP1
RLP2
AtRLP3
AtRLP4
AtRLP5
AtRLP6
AtRLP7
RLP9
CLV2
AtRLP11
RLP12
AtRLP13
AtRLP14
AtRLP15
AtRLP16
TMM
AT2G15042
AtRLP19
AtRLP20
AtRLP21
AtRLP22
AtRLP23
AtRLP24
AtRLP25
AtRLP27, ...
AtRLP28
AtRLP29
AtRLP30
AtRLP31
AtRLP32
AtRLP33
AtRLP53, ...
AtRLP35
AtRLP36
AtRLP37, ...
AtRLP39
ATRLP40
AtRLP41
AtRLP42
AtRLP43
AtRLP44
AtRLP45
AtRLP46
AtRLP47
AtRLP48
AtRLP50
AtRLP52
AtRLP54
AtRLP55
ATRLP56
ATRLP57
Pi-score
Log2-ratio
| 2 |
Fold-Change
0.01
p-value
Biotic
AT-00406 P. syringae pv. maculicola (Col-0) / mock treated leaf samples (Col-0)
9.15
2.29
4.88
<0.001
Chemical
Elicitor
Hormone
Nutrient
Stress
Temperature
Genotype
created with GENEVESTIGATOR

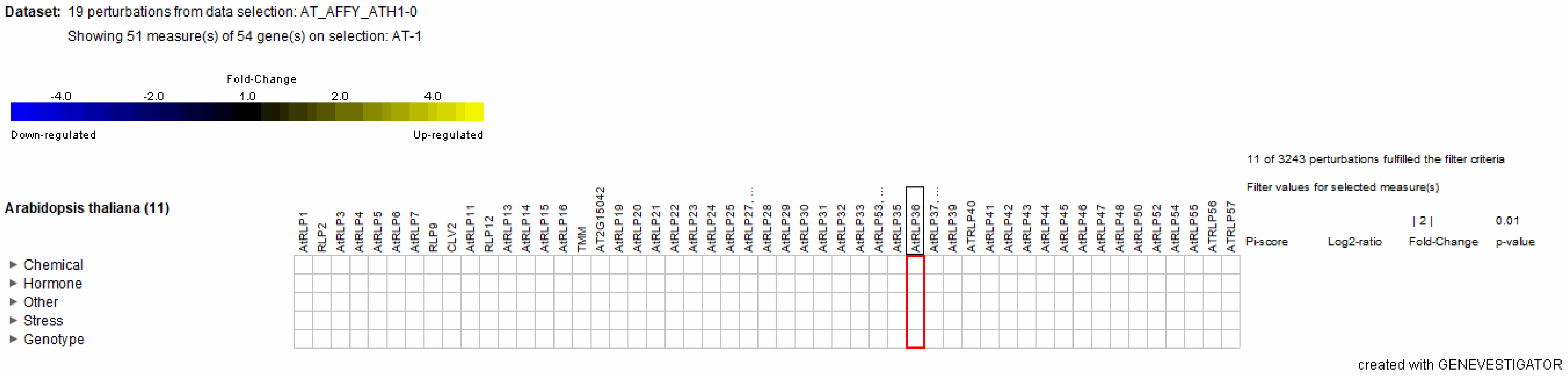

Dataset: 19 perturbations from data selection: AT_AFFY_ATH1-0
Showing 51 measure(s) of 54 gene(s) on selection: AT-1
Fold-Change
-4.0 -2.0 1.0 2.0 4.0
Down-regulated
Up-regulated
11 of 3243 perturbations fulfilled the filter criteria
Filter values for selected measure(s)
Arabidopsis thaliana (11)
AtRLP1 RLP2 AtRLP3 AtRLP4 AtRLP5 AtRLP6 AtRLP7 RLP9 CLV2 AtRLP11 RLP12 AtRLP13 AtRLP14 AtRLP15 AtRLP16 TMM AT2G15042 AtRLP19 AtRLP20 AtRLP21 AtRLP22 AtRLP23 AtRLP24 AtRLP25 AtRLP27, ... AtRLP28 AtRLP29 AtRLP30 AtRLP31 AtRLP32 AtRLP33 AtRLP53, ... AtRLP35 AtRLP36 AtRLP37, ... AtRLP39 ATRLP40 AtRLP41 AtRLP42 AtRLP43 AtRLP44 AtRLP45 AtRLP46 AtRLP47 AtRLP48 AtRLP50 AtRLP52 AtRLP54 AtRLP55 ATRLP56 ATRLP57
| 2 | 0.01
Pi-score Log2-ratio Fold-Change p-value
Chemical
Hormone
Other
Stress
Genotype
created with GENEVESTIGATOR

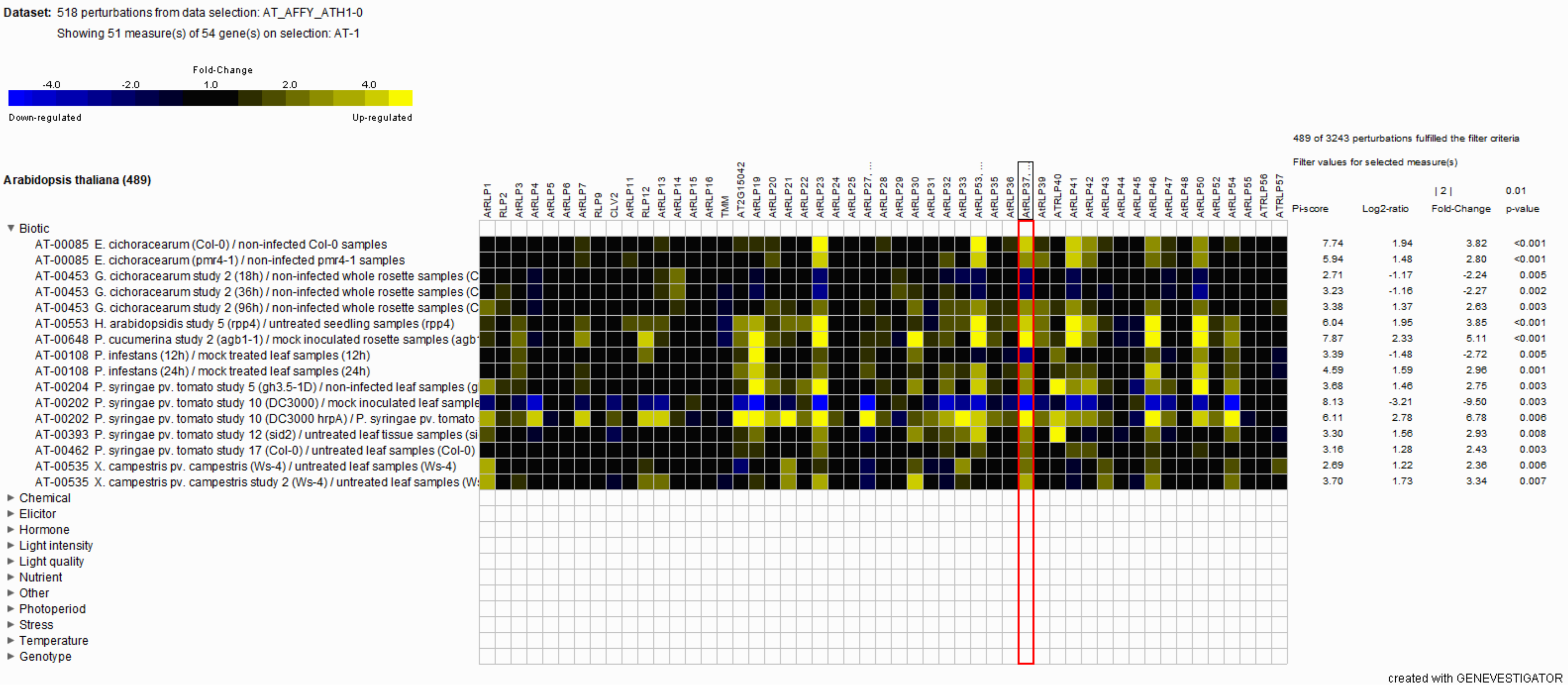
Dataset: 518 perturbations from data selection: AT_AFFY_ATH1-0
Showing 51 measure(s) of 54 gene(s) on selection: AT-1
Fold-Change
-4.0 -2.0 1.0 2.0 4.0
Down-regulated
Up-regulated
489 of 3243 perturbations fulfilled the filter criteria
Filter values for selected measure(s)
Arabidopsis thaliana (489)
AtRLP1 RLP2 AtRLP3 AtRLP4 AtRLP5 AtRLP6 AtRLP7 RLP9 CLV2 AtRLP11 RLP12 AtRLP13 AtRLP14 AtRLP15 AtRLP16 TMM AT2G15042 AtRLP19 AtRLP20 AtRLP21 AtRLP22 AtRLP23 AtRLP24 AtRLP25 AtRLP27, ... AtRLP28 AtRLP29 AtRLP30 AtRLP31 AtRLP32 AtRLP33 AtRLP53, ... AtRLP35 AtRLP36 AtRLP37, ... AtRLP39 ATRLP40 AtRLP41 AtRLP42 AtRLP43 AtRLP44 AtRLP45 AtRLP46 AtRLP47 AtRLP48 AtRLP50 AtRLP52 AtRLP54 AtRLP55 ATRLP56 ATRLP57
| 2 | 0.01
Pi-score Log2-ratio Fold-Change p-value
Biotic
AT-00085 E. cichoracearum (Col-0) / non-infected Col-0 samples 7.74 1.94 3.82 <0.001
AT-00085 E. cichoracearum (pmr4-1) / non-infected pmr4-1 samples 5.94 1.48 2.80 <0.001
AT-00453 G. cichoracearum study 2 (18h) / non-infected whole rosette samples (C 2.71 -1.17 -2.24 0.005
AT-00453 G. cichoracearum study 2 (36h) / non-infected whole rosette samples (C 3.23 -1.16 -2.27 0.002
AT-00453 G. cichoracearum study 2 (96h) / non-infected whole rosette samples (C 3.38 1.37 2.63 0.003
AT-00553 H. arabidopsidis study 5 (rpp4) / untreated seedling samples (rpp4) 6.04 1.95 3.85 <0.001
AT-00648 P. cucumerina study 2 (agb1-1) / mock inoculated rosette samples (agb1 7.87 2.33 5.11 <0.001
AT-00108 P. infestans (12h) / mock treated leaf samples (12h) 3.39 -1.48 -2.72 0.005
AT-00108 P. infestans (24h) / mock treated leaf samples (24h) 4.59 1.59 2.96 0.001
AT-00204 P. syringae pv. tomato study 5 (gh3.5-1D) / non-infected leaf samples (g 3.68 1.46 2.75 0.003
AT-00202 P. syringae pv. tomato study 10 (DC3000) / mock inoculated leaf sample 8.13 -3.21 -9.50 0.003
AT-00202 P. syringae pv. tomato study 10 (DC3000 hrpA) / P. syringae pv. tomato 6.11 2.78 6.78 0.006
AT-00393 P. syringae pv. tomato study 12 (sid2) / untreated leaf tissue samples (si 3.30 1.56 2.93 0.008
AT-00462 P. syringae pv. tomato study 17 (Col-0) / untreated leaf samples (Col-0) 3.16 1.28 2.43 0.003
AT-00535 X. campestris pv. campestris (Ws-4) / untreated leaf samples (Ws-4) 2.69 1.22 2.36 0.006
AT-00535 X. campestris pv. campestris study 2 (Ws-4) / untreated leaf samples (W 3.70 1.73 3.34 0.007
Chemical
Elicitor
Hormone
Light intensity
Light quality
Nutrient
Other
Photoperiod
Stress
Temperature
Genotype
created with GENEVESTIGATOR

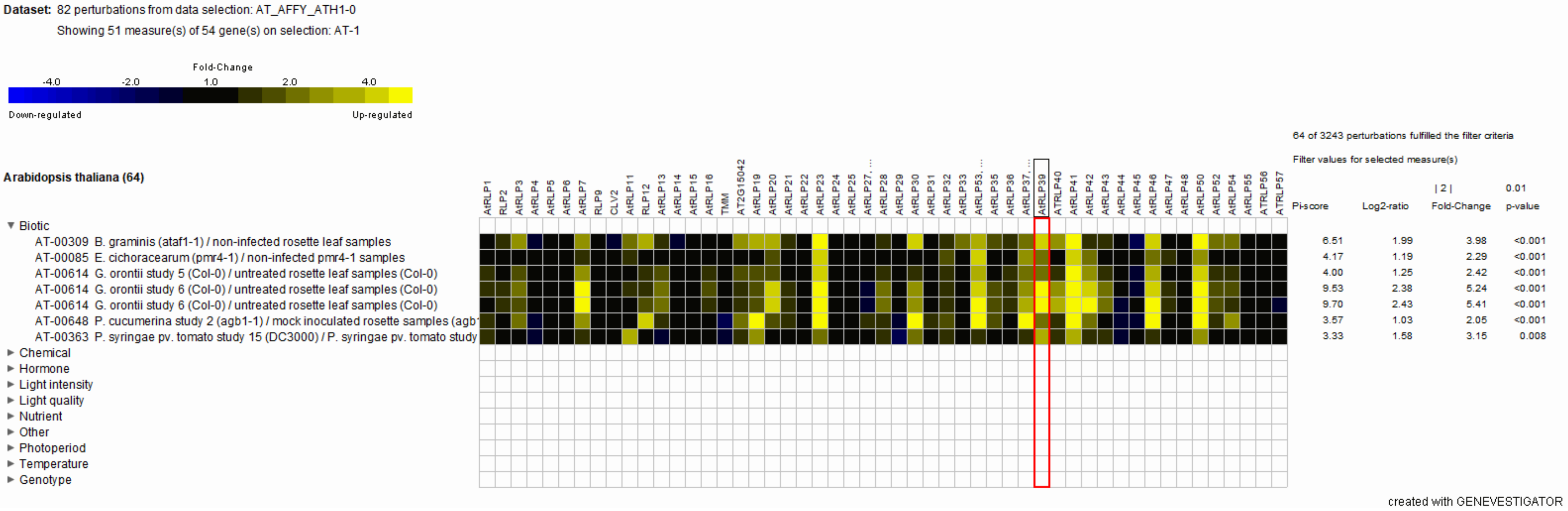

Dataset: 82 perturbations from data selection: AT_AFFY_ATH1-0
Showing 51 measure(s) of 54 gene(s) on selection: AT-1
Fold-Change
-4.0
-2.0
1.0
2.0
4.0
Down-regulated
Up-regulated
64 of 3243 perturbations fulfilled the filter criteria
Filter values for selected measure(s)
Arabidopsis thaliana (64)
AtRLP1
RLP2
AtRLP3
AtRLP4
AtRLP5
AtRLP6
AtRLP7
RLP9
CLV2
AtRLP11
RLP12
AtRLP13
AtRLP14
AtRLP15
AtRLP16
TMM
AT2G15042
AtRLP19
AtRLP20
AtRLP21
AtRLP22
AtRLP23
AtRLP24
AtRLP25
AtRLP27, ...
AtRLP28
AtRLP29
AtRLP30
AtRLP31
AtRLP32
AtRLP33
AtRLP53, ...
AtRLP35
AtRLP36
AtRLP37, ...
AtRLP39
ATRLP40
AtRLP41
AtRLP42
AtRLP43
AtRLP44
AtRLP45
AtRLP46
AtRLP47
AtRLP48
AtRLP50
AtRLP52
AtRLP54
AtRLP55
ATRLP56
ATRLP57
|2|
0.01
Pi-score
Log2-ratio
Fold-Change
p-value
Biotic
AT-00309 B. graminis (ataf1-1) / non-infected rosette leaf samples
6.51 1.99 3.98 <0.001
AT-00085 E. cichoracearum (pmr4-1) / non-infected pmr4-1 samples
4.17 1.19 2.29 <0.001
AT-00614 G. orontii study 5 (Col-0) / untreated rosette leaf samples (Col-0)
4.00 1.25 2.42 <0.001
AT-00614 G. orontii study 6 (Col-0) / untreated rosette leaf samples (Col-0)
9.53 2.38 5.24 <0.001
AT-00614 G. orontii study 6 (Col-0) / untreated rosette leaf samples (Col-0)
9.70 2.43 5.41 <0.001
AT-00648 P. cucumerina study 2 (agb1-1) / mock inoculated rosette samples (agb1
3.57 1.03 2.05 <0.001
AT-00363 P. syringae pv. tomato study 15 (DC3000) / P. syringae pv. tomato study
3.33 1.58 3.15 0.008
Chemical
Hormone
Light intensity
Light quality
Nutrient
Other
Photoperiod
Temperature
Genotype
created with GENEVESTIGATOR

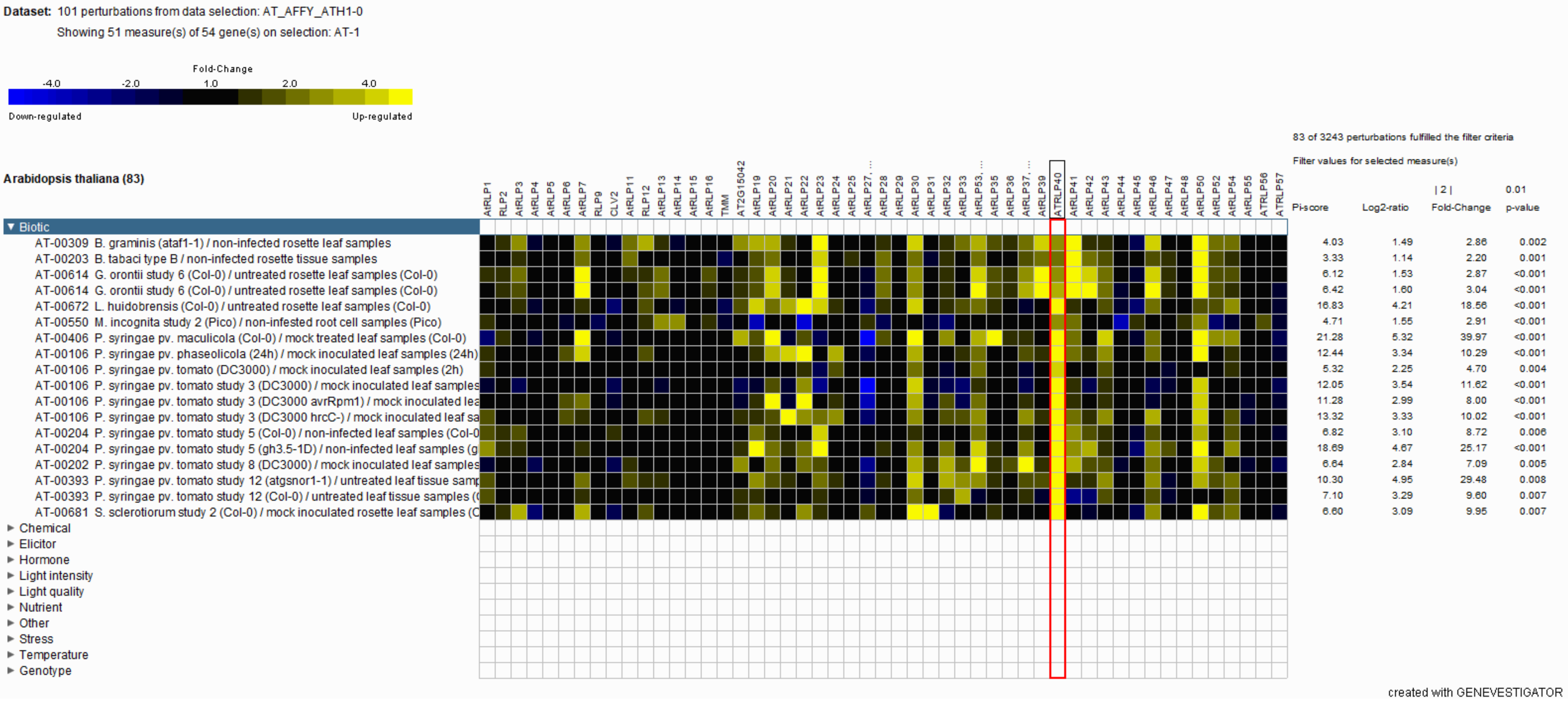

Dataset: 101 perturbations from data selection: AT_AFFY_ATH1-0
Showing 51 measure(s) of 54 gene(s) on selection: AT-1
Fold-Change
-4.0
-2.0
1.0
2.0
4.0
Down-regulated
Up-regulated
83 of 3243 perturbations fulfilled the filter criteria
Filter values for selected measure(s)
Arabidopsis thaliana (83)
AtRLP1
RLP2
AtRLP3
AtRLP4
AtRLP5
AtRLP6
AtRLP7
RLP9
CLV2
AtRLP11
RLP12
AtRLP13
AtRLP14
AtRLP15
AtRLP16
TMM
AT2G15042
AtRLP19
AtRLP20
AtRLP21
AtRLP22
AtRLP23
AtRLP24
AtRLP25
AtRLP27, ...
AtRLP28
AtRLP29
AtRLP30
AtRLP31
AtRLP32
AtRLP33
AtRLP53, ...
AtRLP35
AtRLP36
AtRLP37, ...
AtRLP39
ATRLP40
AtRLP41
AtRLP42
AtRLP43
AtRLP44
AtRLP45
AtRLP46
AtRLP47
AtRLP48
AtRLP50
AtRLP52
AtRLP54
AtRLP55
ATRLP56
ATRLP57
|2|
0.01
Pi-score
Log2-ratio
Fold-Change
p-value
Biotic
AT-00309 B. graminis (ataf1-1) / non-infected rosette leaf samples 4.03 1.49 2.86 0.002
AT-00203 B. tabaci type B / non-infected rosette tissue samples 3.33 1.14 2.20 0.001
AT-00614 G. orontii study 6 (Col-0) / untreated rosette leaf samples (Col-0) 6.12 1.53 2.87 <0.001
AT-00614 G. orontii study 6 (Col-0) / untreated rosette leaf samples (Col-0) 6.42 1.60 3.04 <0.001
AT-00672 L. huidobrensis (Col-0) / untreated rosette leaf samples (Col-0) 16.83 4.21 18.56 <0.001
AT-00550 M. incognita study 2 (Pico) / non-infested root cell samples (Pico) 4.71 1.55 2.91 <0.001
AT-00406 P. syringae pv. maculicola (Col-0) / mock treated leaf samples (Col-0) 21.28 5.32 39.97 <0.001
AT-00106 P. syringae pv. phaseolicola (24h) / mock inoculated leaf samples (24h) 12.44 3.34 10.29 <0.001
AT-00106 P. syringae pv. tomato (DC3000) / mock inoculated leaf samples (2h) 5.32 2.25 4.70 0.004
AT-00106 P. syringae pv. tomato study 3 (DC3000) / mock inoculated leaf samples 12.05 3.54 11.62 <0.001
AT-00106 P. syringae pv. tomato study 3 (DC3000 avrRpm1) / mock inoculated lea 11.28 2.99 8.00 <0.001
AT-00106 P. syringae pv. tomato study 3 (DC3000 hrcC-) / mock inoculated leaf sa 13.32 3.33 10.02 <0.001
AT-00204 P. syringae pv. tomato study 5 (Col-0) / non-infected leaf samples (Col-0 6.82 3.10 8.72 0.006
AT-00204 P. syringae pv. tomato study 5 (gh3.5-1D) / non-infected leaf samples (g 18.69 4.67 25.17 <0.001
AT-00202 P. syringae pv. tomato study 8 (DC3000) / mock inoculated leaf samples 6.64 2.84 7.09 0.005
AT-00393 P. syringae pv. tomato study 12 (atgsnor1-1) / untreated leaf tissue samp 10.30 4.95 29.48 0.008
AT-00393 P. syringae pv. tomato study 12 (Col-0) / untreated leaf tissue samples ((7.10 3.29 9.60 0.007
AT-00681 S. sclerotiorum study 2 (Col-0) / mock inoculated rosette leaf samples (C 6.60 3.09 9.95 0.007
Chemical
Elicitor
Hormone
Light intensity
Light quality
Nutrient
Other
Stress
Temperature
Genotype
created with GENEVESTIGATOR

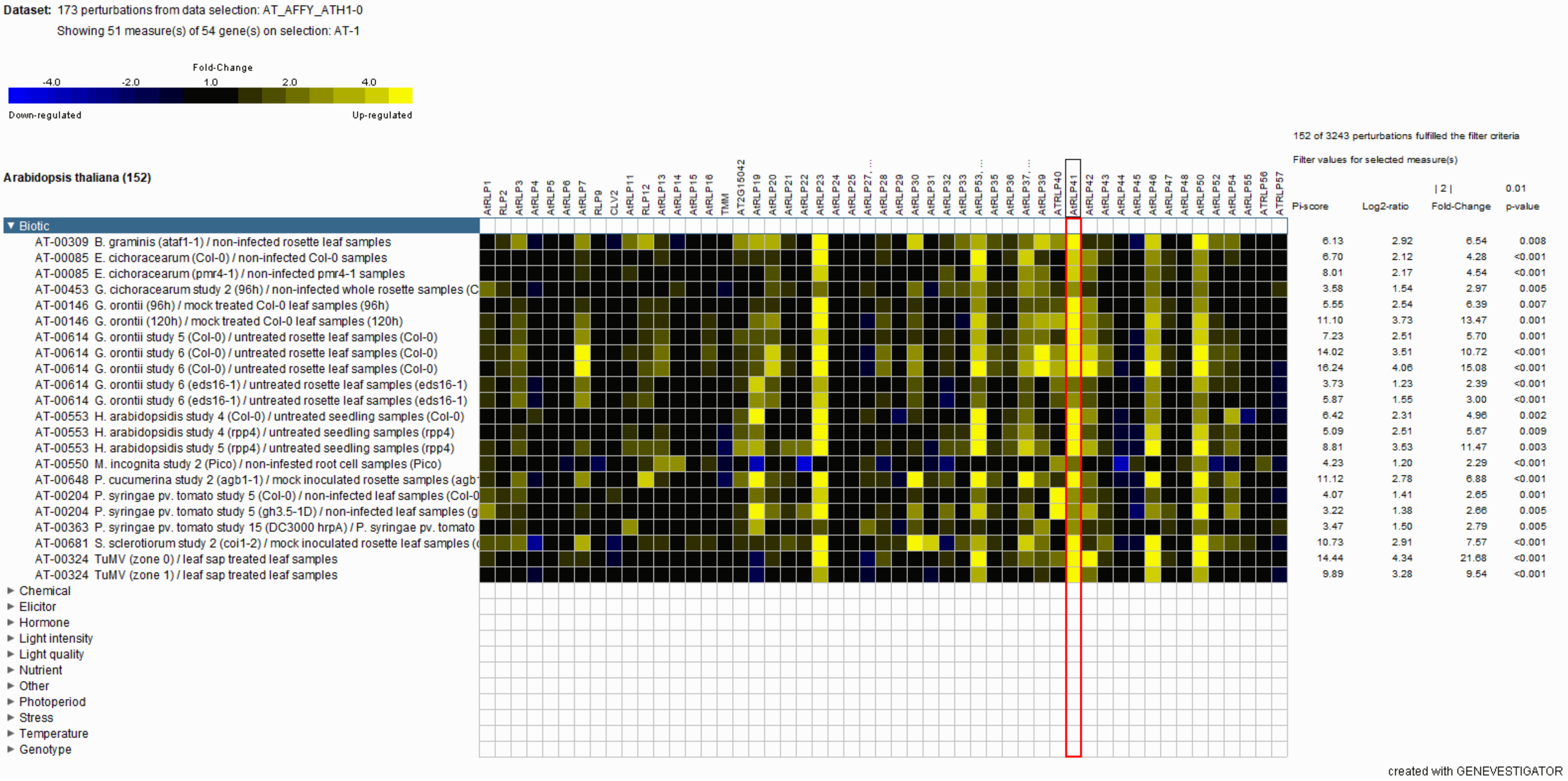
Dataset: 173 perturbations from data selection: AT_AFFY_ATH1-0
Showing 51 measure(s) of 54 gene(s) on selection: AT-1
Fold-Change
-4.0
-2.0
1.0
2.0
4.0
Down-regulated
Up-regulated
152 of 3243 perturbations fulfilled the filter criteria
Filter values for selected measure(s)
Arabidopsis thaliana (152)
AtRLP1
RLP2
AtRLP3
AtRLP4
AtRLP5
AtRLP6
AtRLP7
RLP9
CLV2
AtRLP11
RLP12
AtRLP13
AtRLP14
AtRLP15
AtRLP16
TMM
AT2G15042
AtRLP19
AtRLP20
AtRLP21
AtRLP22
AtRLP23
AtRLP24
AtRLP25
AtRLP27, ...
AtRLP28
AtRLP29
AtRLP30
AtRLP31
AtRLP32
AtRLP33
AtRLP53, ...
AtRLP35
AtRLP36
AtRLP37, ...
AtRLP39
ATRLP40
AtRLP41
AtRLP42
AtRLP43
AtRLP44
AtRLP45
AtRLP46
AtRLP47
AtRLP48
AtRLP50
AtRLP52
AtRLP54
AtRLP55
ATRLP56
ATRLP57
|2|
0.01
Pi-score
Log2-ratio
Fold-Change
p-value
Biotic
AT-00309 B. graminis (ataf1-1) / non-infected rosette leaf samples 6.13 2.92 6.54 0.008
AT-00085 E. cichoracearum (Col-0) / non-infected Col-0 samples 6.70 2.12 4.28 <0.001
AT-00085 E. cichoracearum (pmr4-1) / non-infected pmr4-1 samples 8.01 2.17 4.54 <0.001
AT-00453 G. cichoracearum study 2 (96h) / non-infected whole rosette samples (C 3.58 1.54 2.97 0.005
AT-00146 G. orontii (96h) / mock treated Col-0 leaf samples (96h) 5.55 2.54 6.39 0.007
AT-00146 G. orontii (120h) / mock treated Col-0 leaf samples (120h) 11.10 3.73 13.47 0.001
AT-00614 G. orontii study 5 (Col-0) / untreated rosette leaf samples (Col-0) 7.23 2.51 5.70 0.001
AT-00614 G. orontii study 6 (Col-0) / untreated rosette leaf samples (Col-0) 14.02 3.51 10.72 <0.001
AT-00614 G. orontii study 6 (Col-0) / untreated rosette leaf samples (Col-0) 16.24 4.06 15.08 <0.001
AT-00614 G. orontii study 6 (eds16-1) / untreated rosette leaf samples (eds16-1) 3.73 1.23 2.39 <0.001
AT-00614 G. orontii study 6 (eds16-1) / untreated rosette leaf samples (eds16-1) 5.87 1.55 3.00 <0.001
AT-00553 H. arabidopsidis study 4 (Col-0) / untreated seedling samples (Col-0) 6.42 2.31 4.96 0.002
AT-00553 H. arabidopsidis study 4 (rpp4) / untreated seedling samples (rpp4) 5.09 2.51 5.67 0.009
AT-00553 H. arabidopsidis study 5 (rpp4) / untreated seedling samples (rpp4) 8.81 3.53 11.47 0.003
AT-00550 M. incognita study 2 (Pico) / non-infested root cell samples (Pico) 4.23 1.20 2.29 <0.001
AT-00648 P. cucumerina study 2 (agb1-1) / mock inoculated rosette samples (agb1 11.12 2.78 6.88 <0.001
AT-00204 P. syringae pv. tomato study 5 (Col-0) / non-infected leaf samples (Col-0 4.07 1.41 2.65 0.001
AT-00204 P. syringae pv. tomato study 5 (gh3.5-1D) / non-infected leaf samples (g 3.22 1.38 2.66 0.005
AT-00363 P. syringae pv. tomato study 15 (DC3000 hrpA) / P. syringae pv. tomato 3.47 1.50 2.79 0.005
AT-00681 S. sclerotiorum study 2 (coi1-2) / mock inoculated rosette leaf samples (c 10.73 2.91 7.57 <0.001
AT-00324 TuMV (zone 0) / leaf sap treated leaf samples 14.44 4.34 21.68 <0.001
AT-00324 TuMV (zone 1) / leaf sap treated leaf samples 9.89 3.28 9.54 <0.001
Chemical
Elicitor
Hormone
Light intensity
Light quality
Nutrient
Other
Photoperiod
Stress
Temperature
Genotype
created with GENEVESTIGATOR

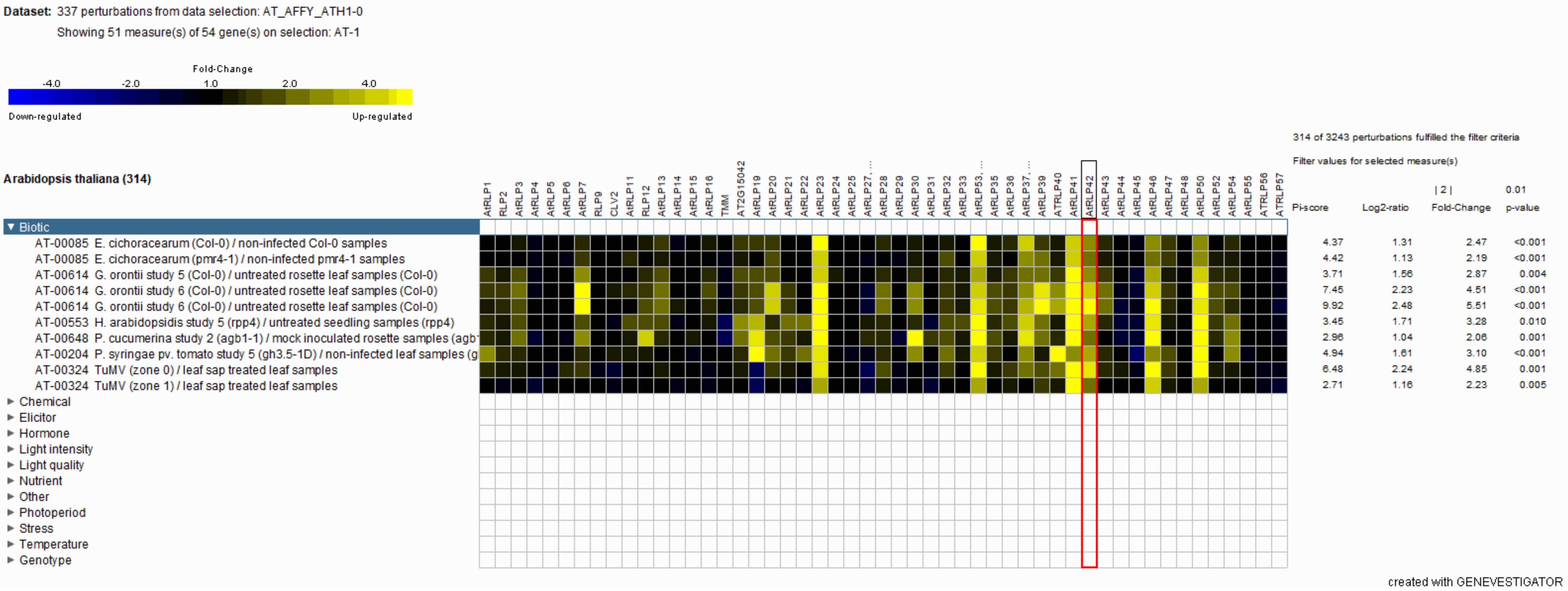

Dataset: 337 perturbations from data selection: AT_AFFY_ATH1-0
Showing 51 measure(s) of 54 gene(s) on selection: AT-1
Fold-Change
-4.0
-2.0
1.0
2.0
4.0
Down-regulated
Up-regulated
314 of 3243 perturbations fulfilled the filter criteria
Filter values for selected measure(s)
Arabidopsis thaliana (314)
AtRLP1 RLP2 AtRLP3 AtRLP4 AtRLP5 AtRLP6 AtRLP7 RLP9 CLV2 AtRLP11 RLP12 AtRLP13 AtRLP14 AtRLP15 AtRLP16 TMM AT2G15042 AtRLP19 AtRLP20 AtRLP21 AtRLP22 AtRLP23 AtRLP24 AtRLP25 AtRLP27, ... AtRLP28 AtRLP29 AtRLP30 AtRLP31 AtRLP32 AtRLP33 AtRLP53, ... AtRLP35 AtRLP36 AtRLP37, ... AtRLP39 ATRLP40 AtRLP41 AtRLP42 AtRLP43 AtRLP44 AtRLP45 AtRLP46 AtRLP47 AtRLP48 AtRLP50 AtRLP52 AtRLP54 AtRLP55 ATRLP56 ATRLP57
| 2 |
0.01
Pi-score
Log2-ratio
Fold-Change
p-value
Biotic
AT-00085 E. cichoracearum (Col-0) / non-infected Col-0 samples 4.37 1.31 2.47 <0.001
AT-00085 E. cichoracearum (pmr4-1) / non-infected pmr4-1 samples 4.42 1.13 2.19 <0.001
AT-00614 G. orontii study 5 (Col-0) / untreated rosette leaf samples (Col-0) 3.71 1.56 2.87 0.004
AT-00614 G. orontii study 6 (Col-0) / untreated rosette leaf samples (Col-0) 7.45 2.23 4.51 <0.001
AT-00614 G. orontii study 6 (Col-0) / untreated rosette leaf samples (Col-0) 9.92 2.48 5.51 <0.001
AT-00553 H. arabidopsidis study 5 (rpp4) / untreated seedling samples (rpp4) 3.45 1.71 3.28 0.010
AT-00648 P. cucumerina study 2 (agb1-1) / mock inoculated rosette samples (agb1 2.96 1.04 2.06 0.001
AT-00204 P. syringae pv. tomato study 5 (gh3.5-1D) / non-infected leaf samples (g 4.94 1.61 3.10 <0.001
AT-00324 TuMV (zone 0) / leaf sap treated leaf samples 6.48 2.24 4.85 0.001
AT-00324 TuMV (zone 1) / leaf sap treated leaf samples 2.71 1.16 2.23 0.005
Chemical
Elicitor
Hormone
Light intensity
Light quality
Nutrient
Other
Photoperiod
Stress
Temperature
Genotype
created with GENEVESTIGATOR

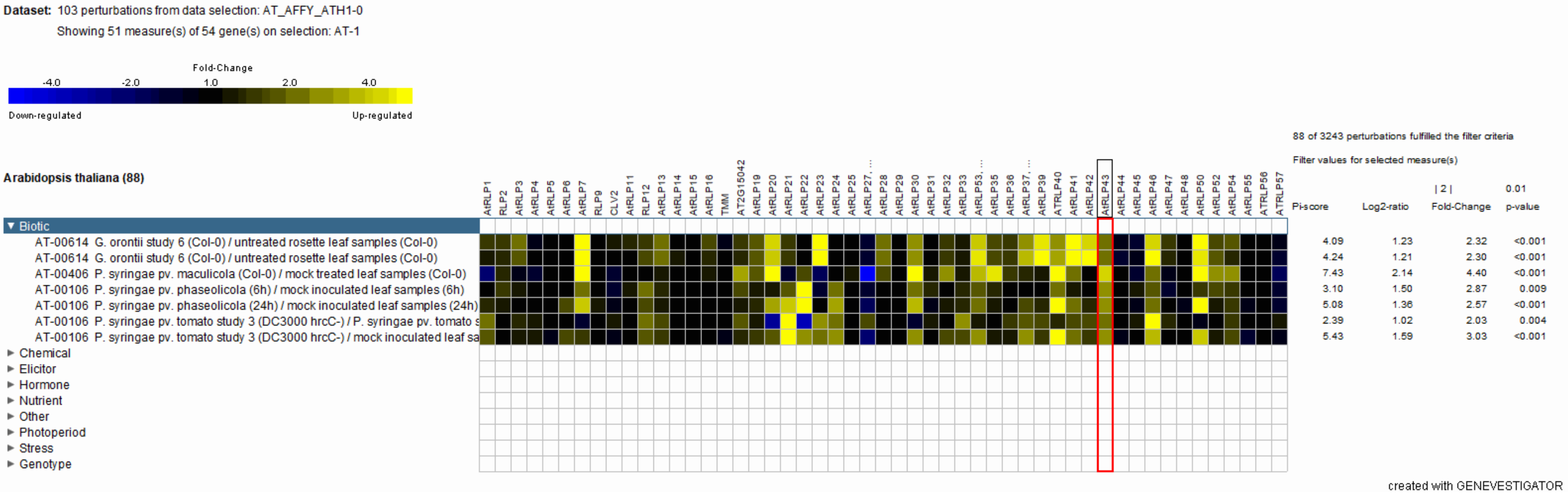

Dataset: 103 perturbations from data selection: AT_AFFY_ATH1-0
Showing 51 measure(s) of 54 gene(s) on selection: AT-1
Fold-Change
-4.0
-2.0
1.0
2.0
4.0
Down-regulated
Up-regulated
88 of 3243 perturbations fulfilled the filter criteria
Filter values for selected measure(s)
Arabidopsis thaliana (88)
AtRLP1
RLP2
AtRLP3
AtRLP4
AtRLP5
AtRLP6
AtRLP7
RLP9
CLV2
AtRLP11
RLP12
AtRLP13
AtRLP14
AtRLP15
AtRLP16
TMM
AT2G15042
AtRLP19
AtRLP20
AtRLP21
AtRLP22
AtRLP23
AtRLP24
AtRLP25
AtRLP27, ...
AtRLP28
AtRLP29
AtRLP30
AtRLP31
AtRLP32
AtRLP33
AtRLP53, ...
AtRLP35
AtRLP36
AtRLP37, ...
AtRLP39
ATRLP40
AtRLP41
AtRLP42
AtRLP43
AtRLP44
AtRLP45
AtRLP46
AtRLP47
AtRLP48
AtRLP50
AtRLP52
AtRLP54
AtRLP55
ATRLP56
ATRLP57
| 2 |
0.01
Pi-score
Log2-ratio
Fold-Change
p-value
Biotic
AT-00614 G. orontii study 6 (Col-0) / untreated rosette leaf samples (Col-0) 4.09 1.23 2.32 <0.001
AT-00614 G. orontii study 6 (Col-0) / untreated rosette leaf samples (Col-0) 4.24 1.21 2.30 <0.001
AT-00406 P. syringae pv. maculicola (Col-0) / mock treated leaf samples (Col-0) 7.43 2.14 4.40 <0.001
AT-00106 P. syringae pv. phaseolicola (6h) / mock inoculated leaf samples (6h) 3.10 1.50 2.87 0.009
AT-00106 P. syringae pv. phaseolicola (24h) / mock inoculated leaf samples (24h) 5.08 1.36 2.57 <0.001
AT-00106 P. syringae pv. tomato study 3 (DC3000 hrcC-) / P. syringae pv. tomato s 2.39 1.02 2.03 0.004
AT-00106 P. syringae pv. tomato study 3 (DC3000 hrcC-) / mock inoculated leaf sa 5.43 1.59 3.03 <0.001
Chemical
Elicitor
Hormone
Nutrient
Other
Photoperiod
Stress
Genotype
created with GENEVESTIGATOR

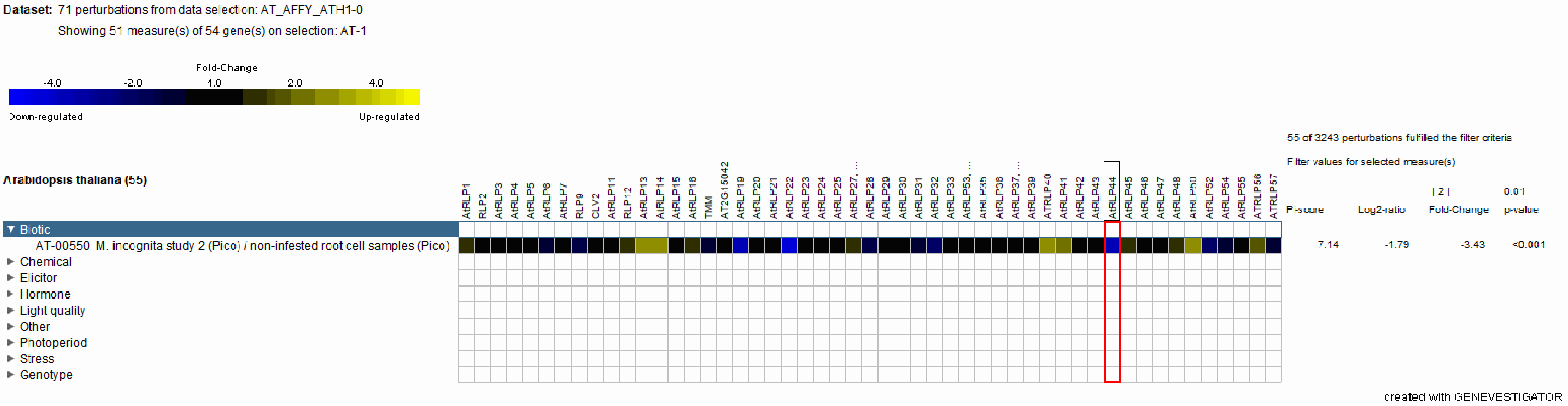
Dataset: 71 perturbations from data selection: AT_AFFY_ATH1-0
Showing 51 measure(s) of 54 gene(s) on selection: AT-1
Fold-Change
-4.0
-2.0
1.0
2.0
4.0
Down-regulated
Up-regulated
55 of 3243 perturbations fulfilled the filter criteria
Filter values for selected measure(s)
Arabidopsis thaliana (55)
AtRLP1 RLP2 AtRLP3 AtRLP4 AtRLP5 AtRLP6 AtRLP7 RLP9 CLV2 AtRLP11 RLP12 AtRLP13 AtRLP14 AtRLP15 AtRLP16 TMM AT2G15042 AtRLP19 AtRLP20 AtRLP21 AtRLP22 AtRLP23 AtRLP24 AtRLP25 AtRLP27, ... AtRLP28 AtRLP29 AtRLP30 AtRLP31 AtRLP32 AtRLP33 AtRLP53, ... AtRLP35 AtRLP36 AtRLP37, ... AtRLP39 ATRLP40 AtRLP41 AtRLP42 AtRLP43 AtRLP44 AtRLP45 AtRLP46 AtRLP47 AtRLP48 AtRLP50 AtRLP52 AtRLP54 AtRLP55 ATRLP56 ATRLP57
| 2 |
0.01
Pi-score
Log2-ratio
Fold-Change
p-value
Biotic
AT-00550 M. incognita study 2 (Pico) / non-infested root cell samples (Pico)
7.14
-1.79
-3.43
<0.001
Chemical
Elicitor
Hormone
Light quality
Other
Photoperiod
Stress
Genotype
created with GENEVESTIGATOR

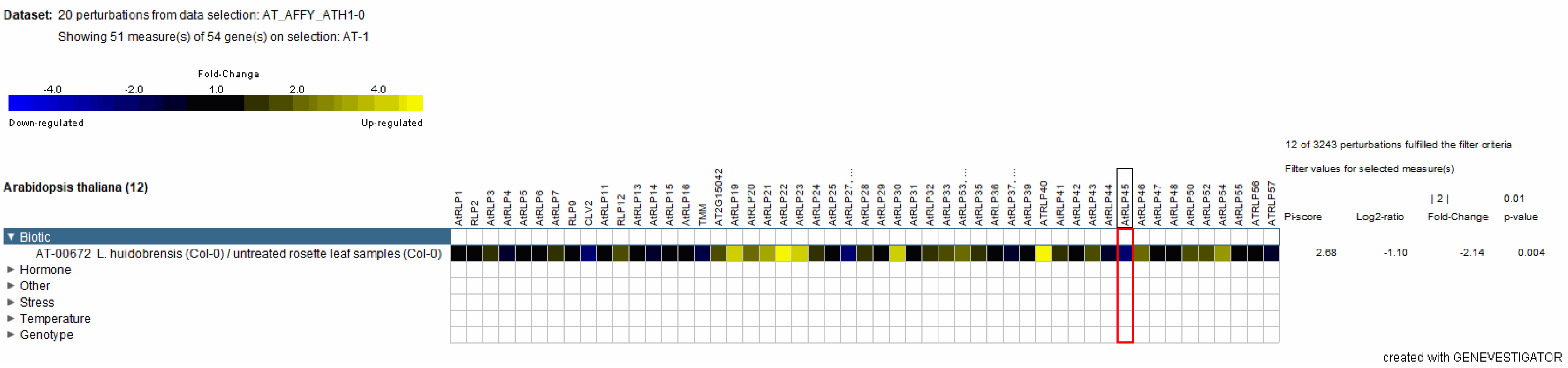

Dataset: 20 perturbations from data selection: AT_AFFY_ATH1-0
Showing 51 measure(s) of 54 gene(s) on selection: AT-1
Fold-Change
-4.0
-2.0
1.0
2.0
4.0
Down-regulated
Up-regulated
12 of 3243 perturbations fulfilled the filter criteria
Filter values for selected measure(s)
Arabidopsis thaliana (12)
AtRLP1
RLP2
AtRLP3
AtRLP4
AtRLP5
AtRLP6
AtRLP7
RLP9
CLV2
AtRLP11
RLP12
AtRLP13
AtRLP14
AtRLP15
AtRLP16
TMM
AT2G15042
AtRLP19
AtRLP20
AtRLP21
AtRLP22
AtRLP23
AtRLP24
AtRLP25
AtRLP27, ...
AtRLP28
AtRLP29
AtRLP30
AtRLP31
AtRLP32
AtRLP33
AtRLP53, ...
AtRLP35
AtRLP36
AtRLP37, ...
AtRLP39
ATRLP40
AtRLP41
AtRLP42
AtRLP43
AtRLP44
AtRLP45
AtRLP46
AtRLP47
AtRLP48
AtRLP50
AtRLP52
AtRLP54
AtRLP55
ATRLP56
ATRLP57
| 2 |
0.01
Pi-score
Log2-ratio
Fold-Change
p-value
Biotic
AT-00672 L. huidobrensis (Col-0) / untreated rosette leaf samples (Col-0)
2.68
-1.10
-2.14
0.004
Hormone
Other
Stress
Temperature
Genotype
created with GENEVESTIGATOR

Dataset: 229 perturbations from data selection: AT_AFFY_ATH1-0

Showing 51 measure(s) of 54 gene(s) on selection: AT-1

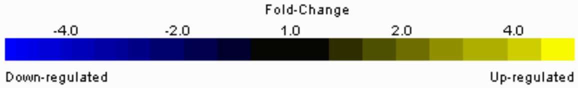

201 of 3243 perturbations fulfilled the filter criteria

Filter values for selected measure(s)

Arabidopsis thaliana (201)

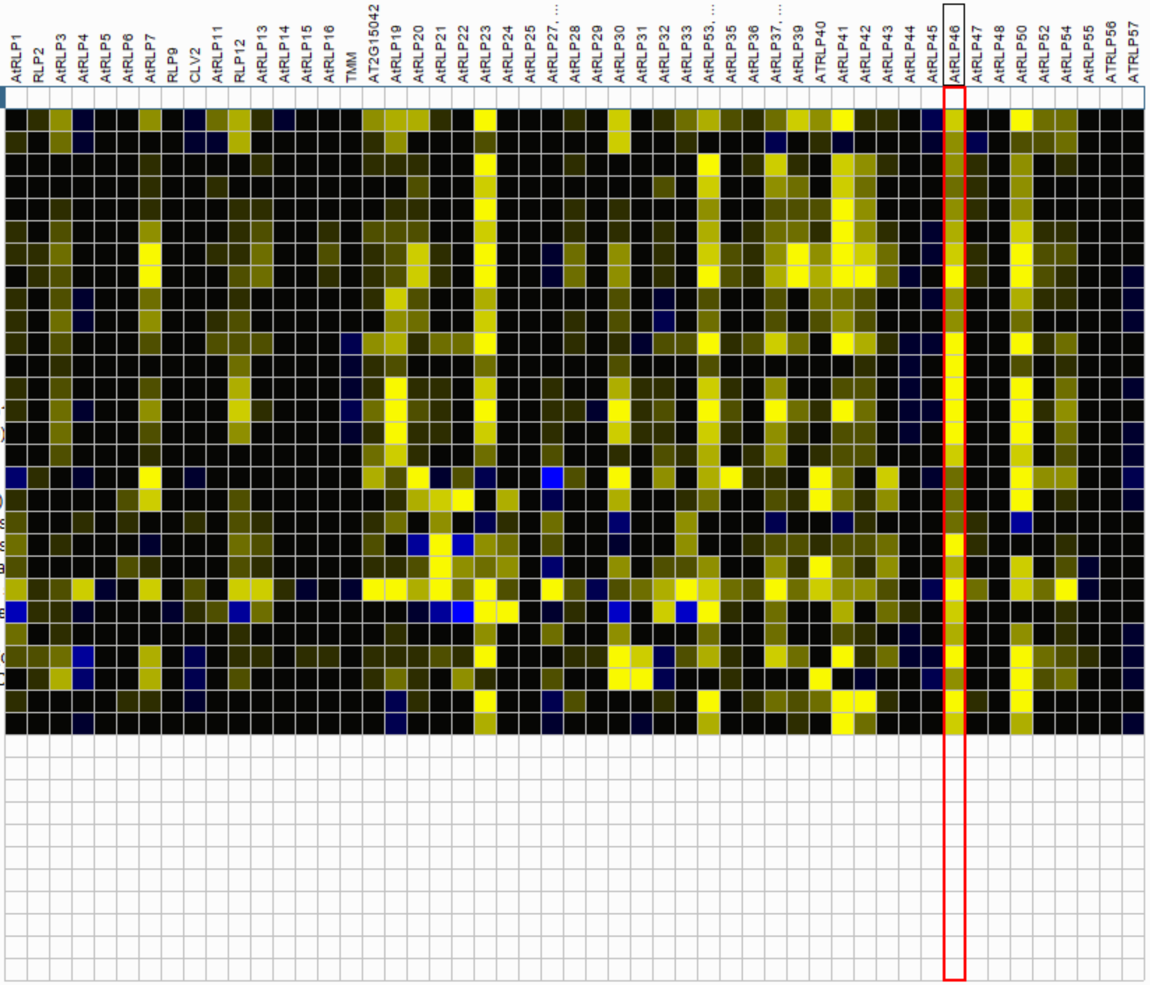

	Pi-score	Log2-ratio	| 2 | Fold-Change	0.01 p-value
▼ Biotic				
AT-00309 B. graminis (ataf1-1) / non-infected rosette leaf samples	4.74	2.13	4.11	0.006
AT-00309 B. graminis (Col-0) / non-infected rosette leaf samples	5.12	1.33	2.52	<0.001
AT-00085 E. cichoracearum (Col-0) / non-infected Col-0 samples	4.83	1.40	2.63	<0.001
AT-00085 E. cichoracearum (pmr4-1) / non-infected pmr4-1 samples	4.44	1.25	2.38	<0.001
AT-00146 G. orontii (96h) / mock treated Col-0 leaf samples (96h)	2.85	1.38	2.60	0.009
AT-00614 G. orontii study 5 (Col-0) / untreated rosette leaf samples (Col-0)	4.20	1.86	3.35	0.006
AT-00614 G. orontii study 6 (Col-0) / untreated rosette leaf samples (Col-0)	6.47	2.29	4.47	0.002
AT-00614 G. orontii study 6 (Col-0) / untreated rosette leaf samples (Col-0)	9.70	2.75	6.27	<0.001
AT-00614 G. orontii study 6 (eds16-1) / untreated rosette leaf samples (eds16-1)	4.54	1.33	2.53	<0.001
AT-00614 G. orontii study 6 (eds16-1) / untreated rosette leaf samples (eds16-1)	5.84	1.57	2.97	<0.001
AT-00553 H. arabidopsidis study 5 (rpp4) / untreated seedling samples (rpp4)	5.06	2.37	5.15	0.007
AT-00638 P. cucumerina (aba1-6) / mock inoculated rosette samples (aba1-6)	5.60	2.31	5.23	0.004
AT-00638 P. cucumerina (Col-0) / mock inoculated rosette samples (Col-0)	12.90	3.23	9.35	<0.001
AT-00648 P. cucumerina study 2 (agb1-1) / mock inoculated rosette samples (agb1	14.38	3.59	12.22	<0.001
AT-00648 P. cucumerina study 2 (Col-0) / mock inoculated rosette samples (Col-0)	13.41	3.35	10.20	<0.001
AT-00108 P. infestans (24h) / mock treated leaf samples (24h)	7.75	1.94	3.84	<0.001
AT-00406 P. syringae pv. maculicola (Col-0) / mock treated leaf samples (Col-0)	4.90	1.23	2.33	<0.001
AT-00106 P. syringae pv. phaseolicola (24h) / mock inoculated leaf samples (24h)	3.03	1.17	2.23	0.003
AT-00106 P. syringae pv. tomato study 2 (DC3000 hrcC-) / P. syringae pv. tomato s	3.42	1.05	2.07	<0.001
AT-00106 P. syringae pv. tomato study 3 (DC3000 hrcC-) / P. syringae pv. tomato s	7.87	2.42	5.41	<0.001
AT-00106 P. syringae pv. tomato study 3 (DC3000 hrcC-) / mock inoculated leaf sa	5.12	1.91	3.78	0.002
AT-00202 P. syringae pv. tomato study 10 (DC3000 hrpA) / P. syringae pv. tomato	7.33	2.99	7.53	0.003
AT-00391 P. syringae pv. tomato study 11 (penta) / untreated leaf disc samples (pe	6.36	1.94	3.92	<0.001
AT-00585 R. solani (AG8) / mock inoculated whole plant samples	3.51	1.68	3.32	0.008
AT-00681 S. sclerotiorum study 2 (coi1-2) / mock inoculated rosette leaf samples (c	8.08	2.98	7.39	0.002
AT-00681 S. sclerotiorum study 2 (Col-0) / mock inoculated rosette leaf samples (C	2.90	1.28	2.49	0.006
AT-00324 TuMV (zone 0) / leaf sap treated leaf samples	8.26	2.59	6.03	<0.001
AT-00324 TuMV (zone 1) / leaf sap treated leaf samples	5.82	2.15	4.49	0.002
► Chemical				
► Elicitor				
► Hormone				
► Light intensity				
► Light quality				
► Nutrient				
► Other				
► Photoperiod				
► Stress				
► Temperature				
► Genotype				

created with GENEVESTIGATOR

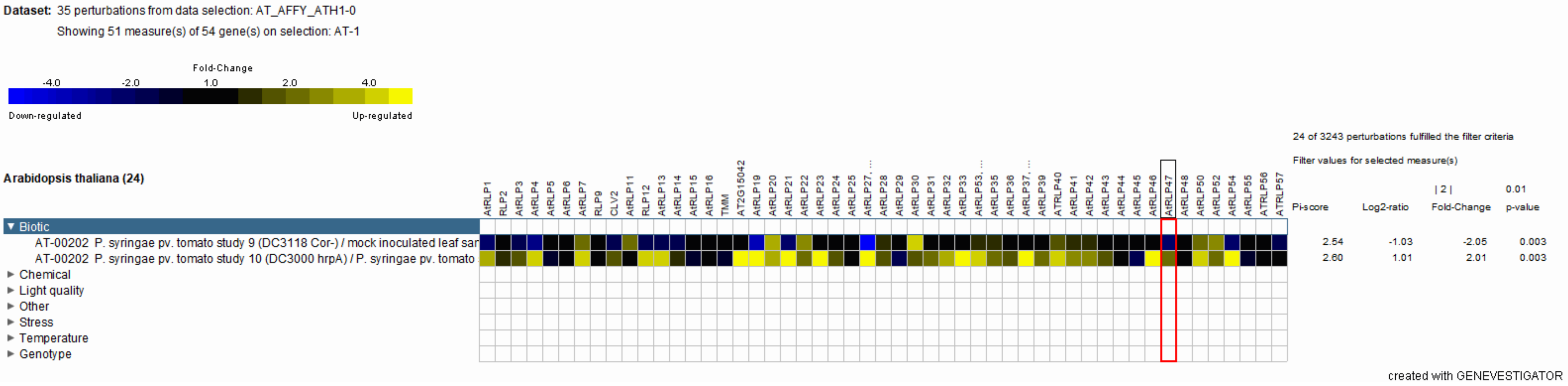
Dataset: 35 perturbations from data selection: AT_AFFY_ATH1-0
Showing 51 measure(s) of 54 gene(s) on selection: AT-1
Fold-Change
-4.0
-2.0
1.0
2.0
4.0
Down-regulated
Up-regulated
24 of 3243 perturbations fulfilled the filter criteria
Filter values for selected measure(s)
Arabidopsis thaliana (24)
AtRLP1 RLP2 AtRLP3 AtRLP4 AtRLP5 AtRLP6 AtRLP7 RLP9 CLV2 AtRLP11 RLP12 AtRLP13 AtRLP14 AtRLP15 AtRLP16 TMM AT2G15042 AtRLP19 AtRLP20 AtRLP21 AtRLP22 AtRLP23 AtRLP24 AtRLP25 AtRLP27, ... AtRLP28 AtRLP29 AtRLP30 AtRLP31 AtRLP32 AtRLP33 AtRLP53, ... AtRLP35 AtRLP36 AtRLP37, ... AtRLP39 ATRLP40 AtRLP41 AtRLP42 AtRLP43 AtRLP44 AtRLP45 AtRLP46 AtRLP47 AtRLP48 AtRLP50 AtRLP52 AtRLP54 AtRLP55 ATRLP56 ATRLP57
| 2 |
0.01
Pi-score
Log2-ratio
Fold-Change
p-value
Biotic
AT-00202 P. syringae pv. tomato study 9 (DC3118 Cor-) / mock inoculated leaf sar
2.54 -1.03 -2.05 0.003
AT-00202 P. syringae pv. tomato study 10 (DC3000 hrpA) / P. syringae pv. tomato
2.60 1.01 2.01 0.003
Chemical
Light quality
Other
Stress
Temperature
Genotype
created with GENEVESTIGATOR

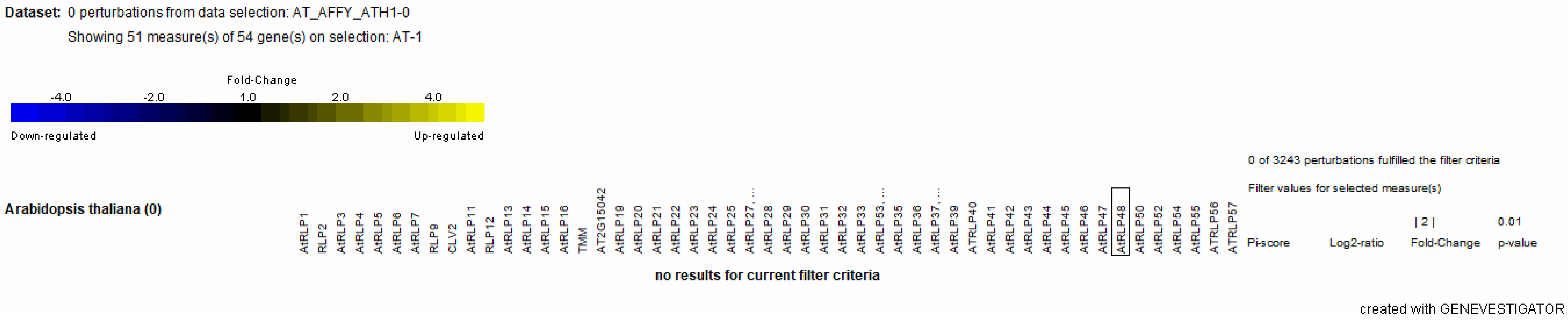

Dataset: 0 perturbations from data selection: AT_AFFY_ATH1-0
Showing 51 measure(s) of 54 gene(s) on selection: AT-1
Fold-Change
-4.0
-2.0
1.0
2.0
4.0
Down-regulated
Up-regulated
0 of 3243 perturbations fulfilled the filter criteria
Filter values for selected measure(s)
Arabidopsis thaliana (0)
AtRLP1
RLP2
AtRLP3
AtRLP4
AtRLP5
AtRLP6
AtRLP7
RLP9
CLV2
AtRLP11
RLP12
AtRLP13
AtRLP14
AtRLP15
AtRLP16
TMM
AT2G15042
AtRLP19
AtRLP20
AtRLP21
AtRLP22
AtRLP23
AtRLP24
AtRLP25
AtRLP27, ...
AtRLP28
AtRLP29
AtRLP30
AtRLP31
AtRLP32
AtRLP33
AtRLP53, ...
AtRLP35
AtRLP36
AtRLP37, ...
AtRLP39
ATRLP40
AtRLP41
AtRLP42
AtRLP43
AtRLP44
AtRLP45
AtRLP46
AtRLP47
AtRLP48
AtRLP50
AtRLP52
AtRLP54
AtRLP55
ATRLP56
ATRLP57
| 2 |
0.01
Pi-score
Log2-ratio
Fold-Change
p-value
no results for current filter criteria
created with GENEVESTIGATOR

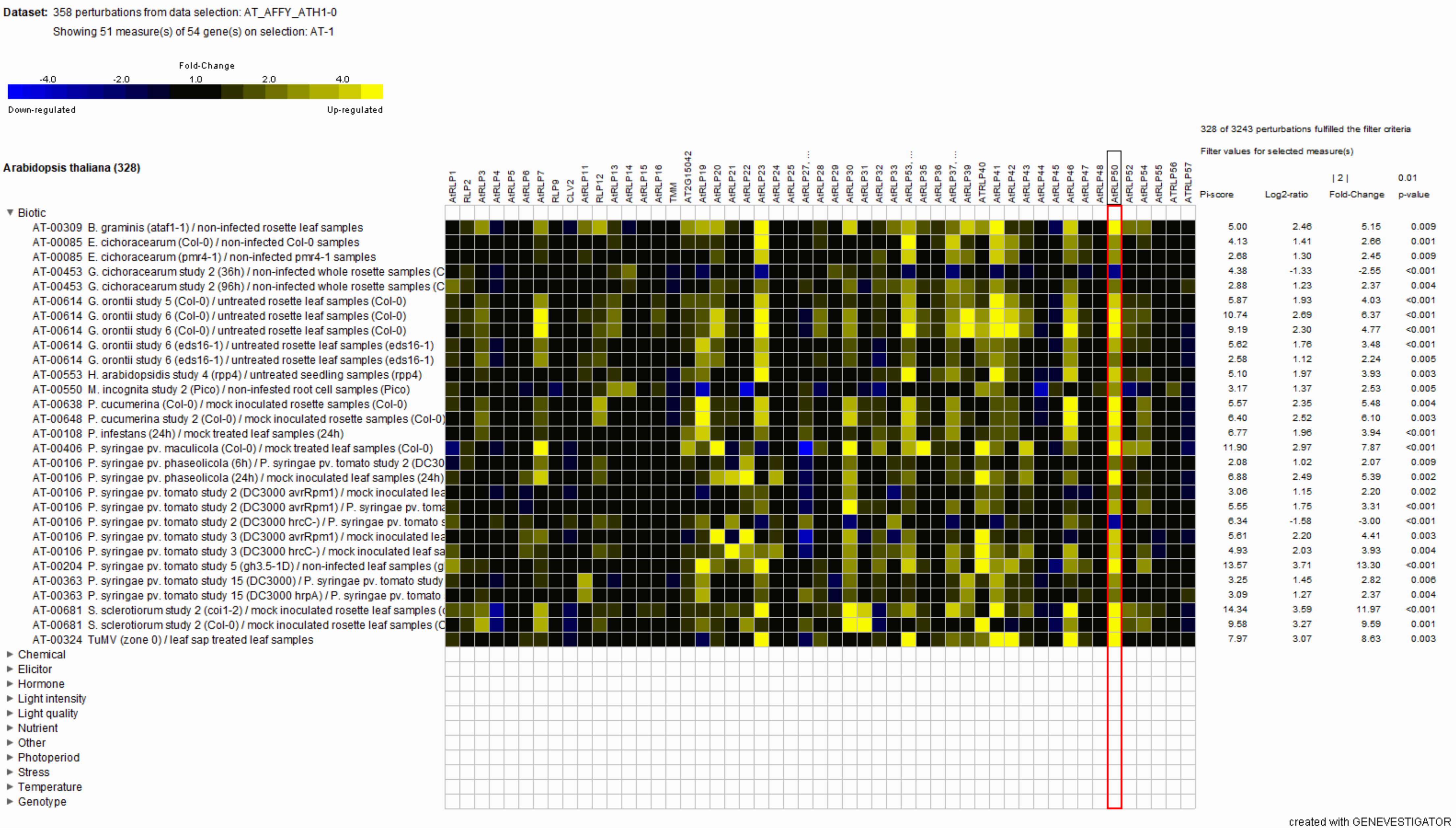

Dataset: 358 perturbations from data selection: AT_AFFY_ATH1-0
Showing 51 measure(s) of 54 gene(s) on selection: AT-1
Fold-Change
-4.0
-2.0
1.0
2.0
4.0
Down-regulated
Up-regulated
328 of 3243 perturbations fulfilled the filter criteria
Filter values for selected measure(s)
Arabidopsis thaliana (328)
AtRLP1
RLP2
AtRLP3
AtRLP4
AtRLP5
AtRLP6
AtRLP7
RLP9
CLV2
AtRLP11
RLP12
AtRLP13
AtRLP14
AtRLP15
AtRLP16
TMM
AT2G15042
AtRLP19
AtRLP20
AtRLP21
AtRLP22
AtRLP23
AtRLP24
AtRLP25
AtRLP27, ...
AtRLP28
AtRLP29
AtRLP30
AtRLP31
AtRLP32
AtRLP33
AtRLP53, ...
AtRLP35
AtRLP36
AtRLP37, ...
AtRLP39
ATRLP40
AtRLP41
AtRLP42
AtRLP43
AtRLP44
AtRLP45
AtRLP46
AtRLP47
AtRLP48
AtRLP50
AtRLP52
AtRLP54
AtRLP55
ATRLP56
ATRLP57
| 2 |
0.01
Pi-score
Log2-ratio
Fold-Change
p-value
Biotic
AT-00309 B. graminis (ataf1-1) / non-infected rosette leaf samples 5.00 2.46 5.15 0.009
AT-00085 E. cichoracearum (Col-0) / non-infected Col-0 samples 4.13 1.41 2.66 0.001
AT-00085 E. cichoracearum (pmr4-1) / non-infected pmr4-1 samples 2.68 1.30 2.45 0.009
AT-00453 G. cichoracearum study 2 (36h) / non-infected whole rosette samples (C 4.38 -1.33 -2.55 <0.001
AT-00453 G. cichoracearum study 2 (96h) / non-infected whole rosette samples (C 2.88 1.23 2.37 0.004
AT-00614 G. orontii study 5 (Col-0) / untreated rosette leaf samples (Col-0) 5.87 1.93 4.03 <0.001
AT-00614 G. orontii study 6 (Col-0) / untreated rosette leaf samples (Col-0) 10.74 2.69 6.37 <0.001
AT-00614 G. orontii study 6 (Col-0) / untreated rosette leaf samples (Col-0) 9.19 2.30 4.77 <0.001
AT-00614 G. orontii study 6 (eds16-1) / untreated rosette leaf samples (eds16-1) 5.62 1.76 3.48 <0.001
AT-00614 G. orontii study 6 (eds16-1) / untreated rosette leaf samples (eds16-1) 2.58 1.12 2.24 0.005
AT-00553 H. arabidopsidis study 4 (rpp4) / untreated seedling samples (rpp4) 5.10 1.97 3.93 0.003
AT-00550 M. incognita study 2 (Pico) / non-infested root cell samples (Pico) 3.17 1.37 2.53 0.005
AT-00638 P. cucumerina (Col-0) / mock inoculated rosette samples (Col-0) 5.57 2.35 5.48 0.004
AT-00648 P. cucumerina study 2 (Col-0) / mock inoculated rosette samples (Col-0) 6.40 2.52 6.10 0.003
AT-00108 P. infestans (24h) / mock treated leaf samples (24h) 6.77 1.96 3.94 <0.001
AT-00406 P. syringae pv. maculicola (Col-0) / mock treated leaf samples (Col-0) 11.90 2.97 7.87 <0.001
AT-00106 P. syringae pv. phaseolicola (6h) / P. syringae pv. tomato study 2 (DC30 2.08 1.02 2.07 0.009
AT-00106 P. syringae pv. phaseolicola (24h) / mock inoculated leaf samples (24h) 6.88 2.49 5.39 0.002
AT-00106 P. syringae pv. tomato study 2 (DC3000 avrRpm1) / mock inoculated lea 3.06 1.15 2.20 0.002
AT-00106 P. syringae pv. tomato study 2 (DC3000 avrRpm1) / P. syringae pv. toma 5.55 1.75 3.31 <0.001
AT-00106 P. syringae pv. tomato study 2 (DC3000 hrcC-) / P. syringae pv. tomato s 6.34 -1.58 -3.00 <0.001
AT-00106 P. syringae pv. tomato study 3 (DC3000 avrRpm1) / mock inoculated lea 5.61 2.20 4.41 0.003
AT-00106 P. syringae pv. tomato study 3 (DC3000 hrcC-) / mock inoculated leaf sa 4.93 2.03 3.93 0.004
AT-00204 P. syringae pv. tomato study 5 (gh3.5-1D) / non-infected leaf samples (g 13.57 3.71 13.30 <0.001
AT-00363 P. syringae pv. tomato study 15 (DC3000) / P. syringae pv. tomato study 3.25 1.45 2.82 0.006
AT-00363 P. syringae pv. tomato study 15 (DC3000 hrpA) / P. syringae pv. tomato 3.09 1.27 2.37 0.004
AT-00681 S. sclerotiorum study 2 (coi1-2) / mock inoculated rosette leaf samples (c 14.34 3.59 11.97 <0.001
AT-00681 S. sclerotiorum study 2 (Col-0) / mock inoculated rosette leaf samples (C 9.58 3.27 9.59 0.001
AT-00324 TuMV (zone 0) / leaf sap treated leaf samples 7.97 3.07 8.63 0.003
Chemical
Elicitor
Hormone
Light intensity
Light quality
Nutrient
Other
Photoperiod
Stress
Temperature
Genotype
created with GENEVESTIGATOR

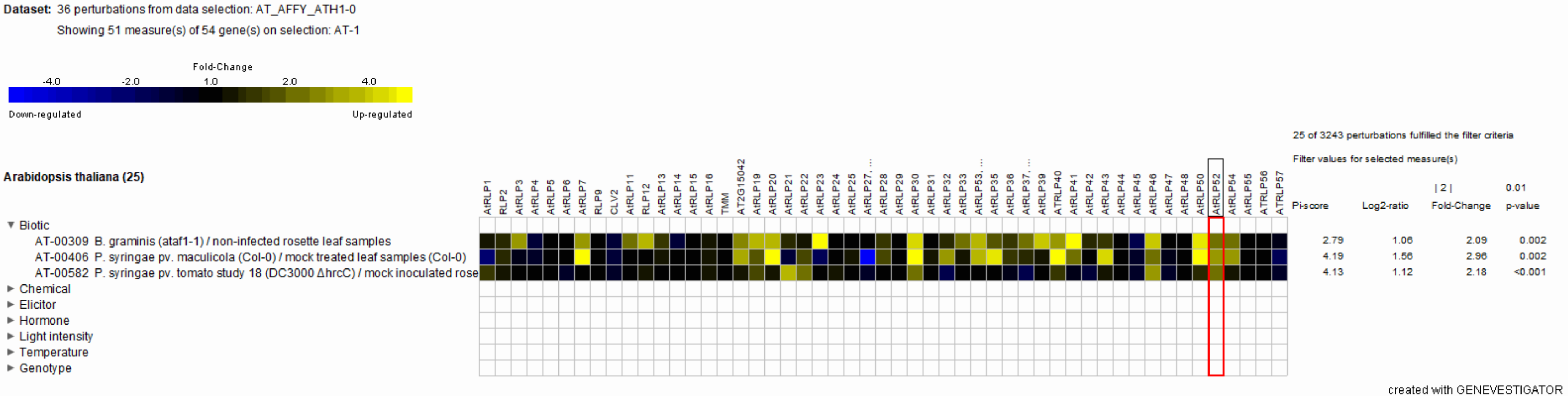
Dataset: 36 perturbations from data selection: AT_AFFY_ATH1-0
Showing 51 measure(s) of 54 gene(s) on selection: AT-1
Fold-Change
-4.0
-2.0
1.0
2.0
4.0
Down-regulated
Up-regulated
25 of 3243 perturbations fulfilled the filter criteria
Filter values for selected measure(s)
Arabidopsis thaliana (25)
AtRLP1
RLP2
AtRLP3
AtRLP4
AtRLP5
AtRLP6
AtRLP7
RLP9
CLV2
AtRLP11
RLP12
AtRLP13
AtRLP14
AtRLP15
AtRLP16
TMM
AT2G15042
AtRLP19
AtRLP20
AtRLP21
AtRLP22
AtRLP23
AtRLP24
AtRLP25
AtRLP27, ...
AtRLP28
AtRLP29
AtRLP30
AtRLP31
AtRLP32
AtRLP33
AtRLP53, ...
AtRLP35
AtRLP36
AtRLP37, ...
AtRLP39
ATRLP40
AtRLP41
AtRLP42
AtRLP43
AtRLP44
AtRLP45
AtRLP46
AtRLP47
AtRLP48
AtRLP50
AtRLP52
AtRLP54
AtRLP55
ATRLP56
ATRLP57
| 2 |
0.01
Pi-score
Log2-ratio
Fold-Change
p-value
▼ Biotic
AT-00309 B. graminis (ataf1-1) / non-infected rosette leaf samples
AT-00406 P. syringae pv. maculicola (Col-0) / mock treated leaf samples (Col-0)
AT-00582 P. syringae pv. tomato study 18 (DC3000 ΔhrcC) / mock inoculated rose
2.79 1.06 2.09 0.002
4.19 1.56 2.96 0.002
4.13 1.12 2.18 <0.001
► Chemical
► Elicitor
► Hormone
► Light intensity
► Temperature
► Genotype
created with GENEVESTIGATOR

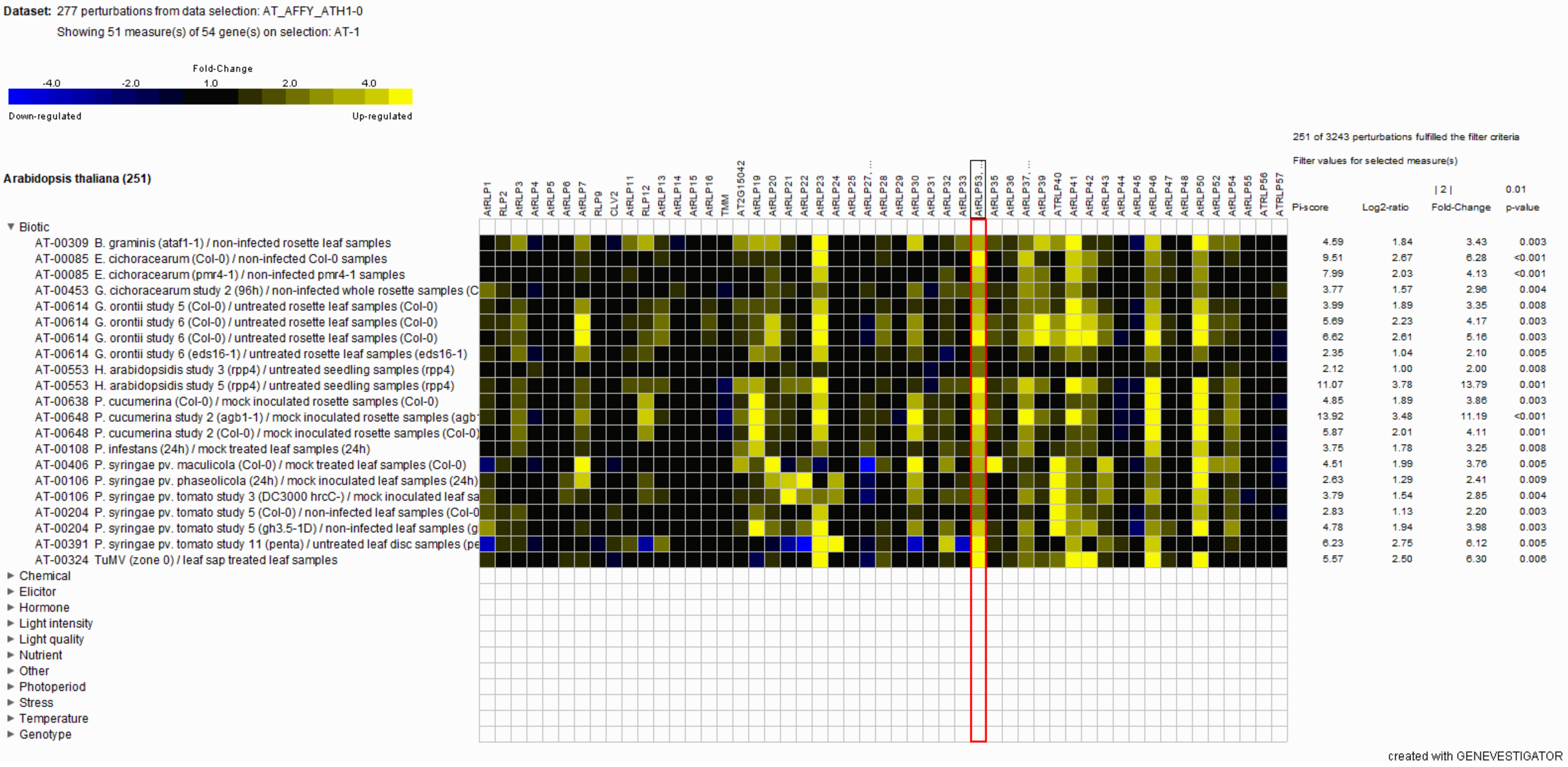

Dataset: 277 perturbations from data selection: AT_AFFY_ATH1-0
Showing 51 measure(s) of 54 gene(s) on selection: AT-1
Fold-Change
-4.0
-2.0
1.0
2.0
4.0
Down-regulated
Up-regulated
251 of 3243 perturbations fulfilled the filter criteria
Filter values for selected measure(s)
Arabidopsis thaliana (251)
AtRLP1
RLP2
AtRLP3
AtRLP4
AtRLP5
AtRLP6
AtRLP7
RLP9
CLV2
AtRLP11
RLP12
AtRLP13
AtRLP14
AtRLP15
AtRLP16
TMM
AT2G15042
AtRLP19
AtRLP20
AtRLP21
AtRLP22
AtRLP23
AtRLP24
AtRLP25
AtRLP27, ...
AtRLP28
AtRLP29
AtRLP30
AtRLP31
AtRLP32
AtRLP33
AtRLP53, ...
AtRLP35
AtRLP36
AtRLP37, ...
AtRLP39
ATRLP40
AtRLP41
AtRLP42
AtRLP43
AtRLP44
AtRLP45
AtRLP46
AtRLP47
AtRLP48
AtRLP50
AtRLP52
AtRLP54
AtRLP55
ATRLP56
ATRLP57
| 2 |
0.01
Pi-score
Log2-ratio
Fold-Change
p-value
Biotic
AT-00309 B. graminis (ataf1-1) / non-infected rosette leaf samples 4.59 1.84 3.43 0.003
AT-00085 E. cichoracearum (Col-0) / non-infected Col-0 samples 9.51 2.67 6.28 <0.001
AT-00085 E. cichoracearum (pmr4-1) / non-infected pmr4-1 samples 7.99 2.03 4.13 <0.001
AT-00453 G. cichoracearum study 2 (96h) / non-infected whole rosette samples (C 3.77 1.57 2.96 0.004
AT-00614 G. orontii study 5 (Col-0) / untreated rosette leaf samples (Col-0) 3.99 1.89 3.35 0.008
AT-00614 G. orontii study 6 (Col-0) / untreated rosette leaf samples (Col-0) 5.69 2.23 4.17 0.003
AT-00614 G. orontii study 6 (Col-0) / untreated rosette leaf samples (Col-0) 6.62 2.61 5.16 0.003
AT-00614 G. orontii study 6 (eds16-1) / untreated rosette leaf samples (eds16-1) 2.35 1.04 2.10 0.005
AT-00553 H. arabidopsidis study 3 (rpp4) / untreated seedling samples (rpp4) 2.12 1.00 2.00 0.008
AT-00553 H. arabidopsidis study 5 (rpp4) / untreated seedling samples (rpp4) 11.07 3.78 13.79 0.001
AT-00638 P. cucumerina (Col-0) / mock inoculated rosette samples (Col-0) 4.85 1.89 3.86 0.003
AT-00648 P. cucumerina study 2 (agb1-1) / mock inoculated rosette samples (agb1 13.92 3.48 11.19 <0.001
AT-00648 P. cucumerina study 2 (Col-0) / mock inoculated rosette samples (Col-0) 5.87 2.01 4.11 0.001
AT-00108 P. infestans (24h) / mock treated leaf samples (24h) 3.75 1.78 3.25 0.008
AT-00406 P. syringae pv. maculicola (Col-0) / mock treated leaf samples (Col-0) 4.51 1.99 3.76 0.005
AT-00106 P. syringae pv. phaseolicola (24h) / mock inoculated leaf samples (24h) 2.63 1.29 2.41 0.009
AT-00106 P. syringae pv. tomato study 3 (DC3000 hrcC-) / mock inoculated leaf sa 3.79 1.54 2.85 0.004
AT-00204 P. syringae pv. tomato study 5 (Col-0) / non-infected leaf samples (Col-0 2.83 1.13 2.20 0.003
AT-00204 P. syringae pv. tomato study 5 (gh3.5-1D) / non-infected leaf samples (g 4.78 1.94 3.98 0.003
AT-00391 P. syringae pv. tomato study 11 (penta) / untreated leaf disc samples (pe 6.23 2.75 6.12 0.005
AT-00324 TuMV (zone 0) / leaf sap treated leaf samples 5.57 2.50 6.30 0.006
Chemical
Elicitor
Hormone
Light intensity
Light quality
Nutrient
Other
Photoperiod
Stress
Temperature
Genotype
created with GENEVESTIGATOR

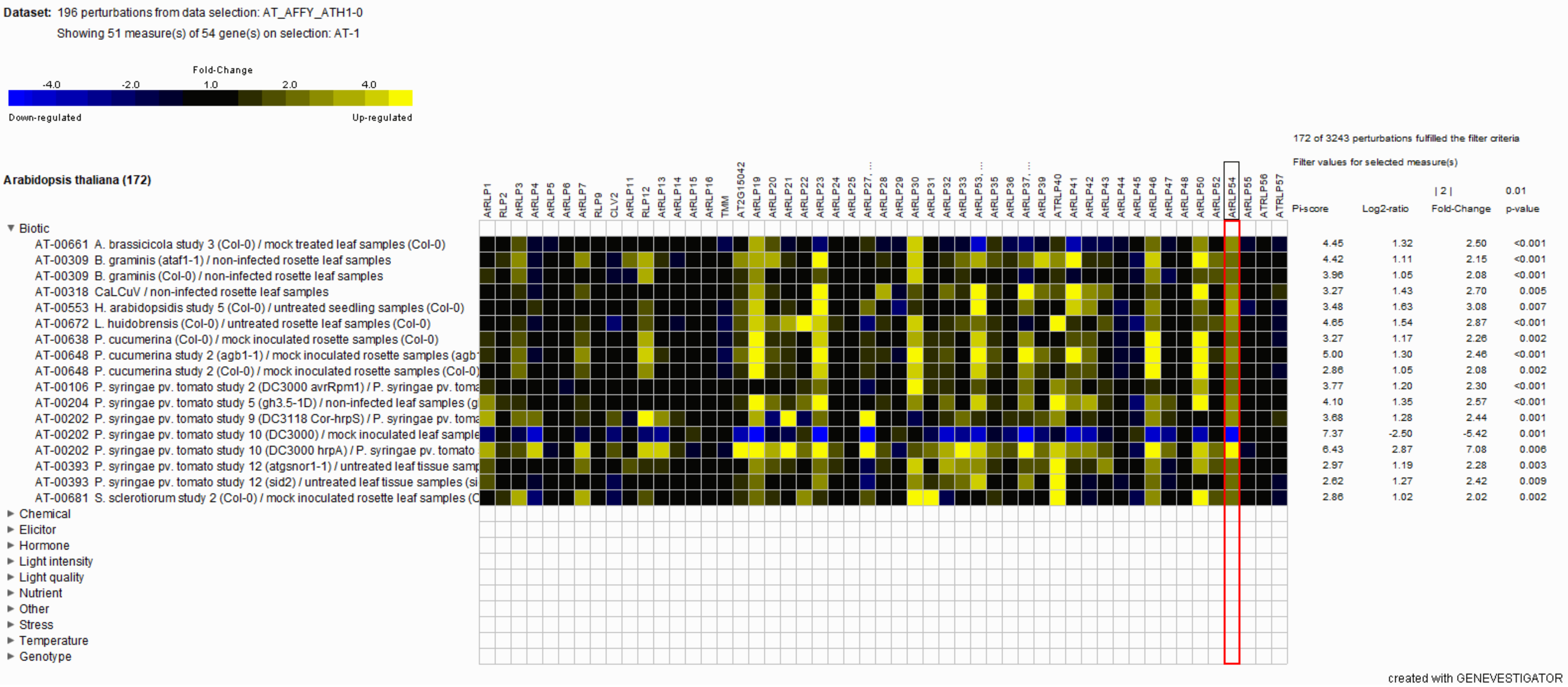

Dataset: 196 perturbations from data selection: AT_AFFY_ATH1-0
Showing 51 measure(s) of 54 gene(s) on selection: AT-1
Fold-Change
-4.0 -2.0 1.0 2.0 4.0
Down-regulated
Up-regulated
172 of 3243 perturbations fulfilled the filter criteria
Filter values for selected measure(s)
Arabidopsis thaliana (172)
AtRLP1 RLP2 AtRLP3 AtRLP4 AtRLP5 AtRLP6 AtRLP7 RLP9 CLV2 AtRLP11 RLP12 AtRLP13 AtRLP14 AtRLP15 AtRLP16 TMM AT2G15042 AtRLP19 AtRLP20 AtRLP21 AtRLP22 AtRLP23 AtRLP24 AtRLP25 AtRLP27, ... AtRLP28 AtRLP29 AtRLP30 AtRLP31 AtRLP32 AtRLP33 AtRLP53, ... AtRLP35 AtRLP36 AtRLP37, ... AtRLP39 ATRLP40 AtRLP41 AtRLP42 AtRLP43 AtRLP44 AtRLP45 AtRLP46 AtRLP47 AtRLP48 AtRLP50 AtRLP52 AtRLP54 AtRLP55 ATRLP56 ATRLP57
|2| 0.01
Pi-score Log2-ratio Fold-Change p-value
Biotic
AT-00661 A. brassicicola study 3 (Col-0) / mock treated leaf samples (Col-0) 4.45 1.32 2.50 <0.001
AT-00309 B. graminis (ataf1-1) / non-infected rosette leaf samples 4.42 1.11 2.15 <0.001
AT-00309 B. graminis (Col-0) / non-infected rosette leaf samples 3.96 1.05 2.08 <0.001
AT-00318 CaLCuV / non-infected rosette leaf samples 3.27 1.43 2.70 0.005
AT-00553 H. arabidopsidis study 5 (Col-0) / untreated seedling samples (Col-0) 3.48 1.63 3.08 0.007
AT-00672 L. huidobrensis (Col-0) / untreated rosette leaf samples (Col-0) 4.65 1.54 2.87 <0.001
AT-00638 P. cucumerina (Col-0) / mock inoculated rosette samples (Col-0) 3.27 1.17 2.26 0.002
AT-00648 P. cucumerina study 2 (agb1-1) / mock inoculated rosette samples (agb1 5.00 1.30 2.46 <0.001
AT-00648 P. cucumerina study 2 (Col-0) / mock inoculated rosette samples (Col-0) 2.86 1.05 2.08 0.002
AT-00106 P. syringae pv. tomato study 2 (DC3000 avrRpm1) / P. syringae pv. toma 3.77 1.20 2.30 <0.001
AT-00204 P. syringae pv. tomato study 5 (gh3.5-1D) / non-infected leaf samples (g 4.10 1.35 2.57 <0.001
AT-00202 P. syringae pv. tomato study 9 (DC3118 Cor-hrpS) / P. syringae pv. toma 3.68 1.28 2.44 0.001
AT-00202 P. syringae pv. tomato study 10 (DC3000) / mock inoculated leaf sample 7.37 -2.50 -5.42 0.001
AT-00202 P. syringae pv. tomato study 10 (DC3000 hrpA) / P. syringae pv. tomato 6.43 2.87 7.08 0.006
AT-00393 P. syringae pv. tomato study 12 (atgsnor1-1) / untreated leaf tissue samp 2.97 1.19 2.28 0.003
AT-00393 P. syringae pv. tomato study 12 (sid2) / untreated leaf tissue samples (si 2.62 1.27 2.42 0.009
AT-00681 S. sclerotiorum study 2 (Col-0) / mock inoculated rosette leaf samples (C 2.86 1.02 2.02 0.002
Chemical
Elicitor
Hormone
Light intensity
Light quality
Nutrient
Other
Stress
Temperature
Genotype
created with GENEVESTIGATOR

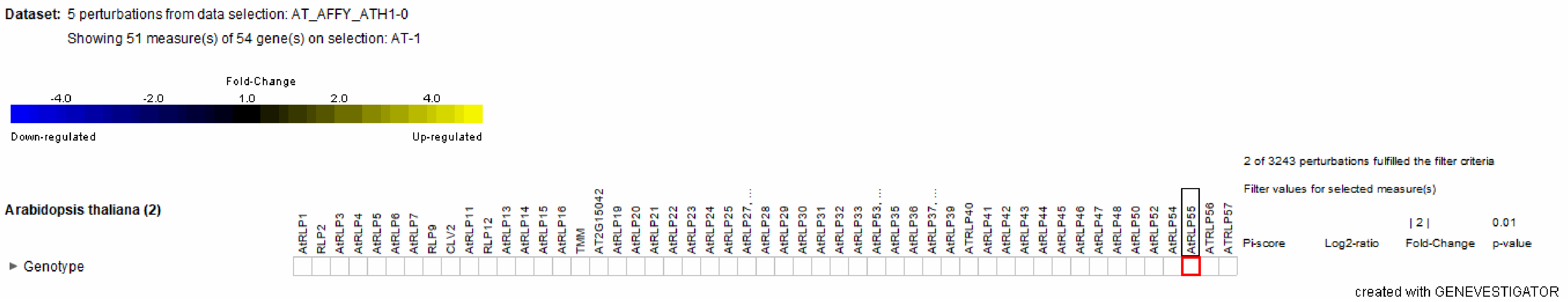
Dataset: 5 perturbations from data selection: AT_AFFY_ATH1-0
Showing 51 measure(s) of 54 gene(s) on selection: AT-1
Fold-Change
-4.0
-2.0
1.0
2.0
4.0
Down-regulated
Up-regulated
2 of 3243 perturbations fulfilled the filter criteria
Filter values for selected measure(s)
Arabidopsis thaliana (2)
AtRLP1
RLP2
AtRLP3
AtRLP4
AtRLP5
AtRLP6
AtRLP7
RLP9
CLV2
AtRLP11
RLP12
AtRLP13
AtRLP14
AtRLP15
AtRLP16
TMM
AT2G15042
AtRLP19
AtRLP20
AtRLP21
AtRLP22
AtRLP23
AtRLP24
AtRLP25
AtRLP27, ...
AtRLP28
AtRLP29
AtRLP30
AtRLP31
AtRLP32
AtRLP33
AtRLP53, ...
AtRLP35
AtRLP36
AtRLP37, ...
AtRLP39
ATRLP40
AtRLP41
AtRLP42
AtRLP43
AtRLP44
AtRLP45
AtRLP46
AtRLP47
AtRLP48
AtRLP50
AtRLP52
AtRLP54
AtRLP55
ATRLP56
ATRLP57
| 2 |
0.01
Pi-score
Log2-ratio
Fold-Change
p-value
Genotype
created with GENEVESTIGATOR

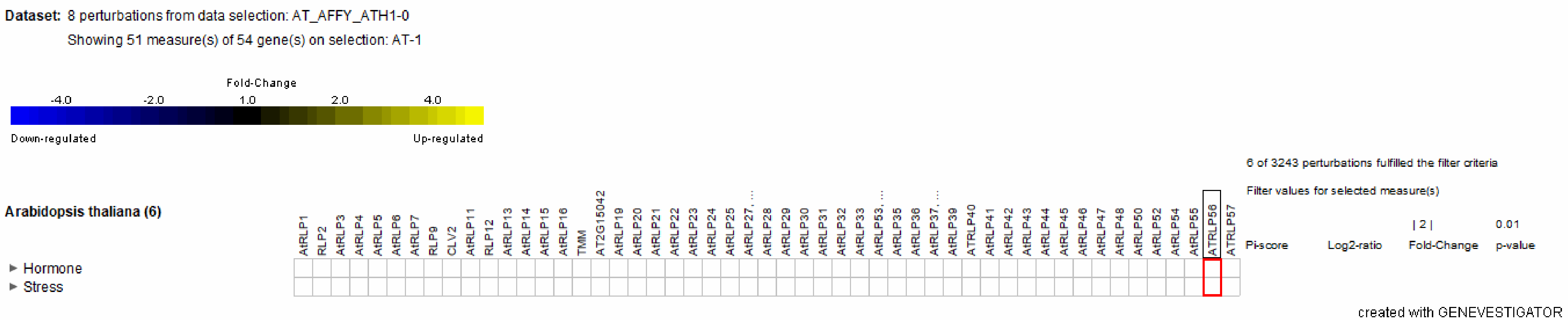

Dataset: 8 perturbations from data selection: AT_AFFY_ATH1-0
Showing 51 measure(s) of 54 gene(s) on selection: AT-1
Fold-Change
-4.0 -2.0 1.0 2.0 4.0
Down-regulated
Up-regulated
6 of 3243 perturbations fulfilled the filter criteria
Filter values for selected measure(s)
Arabidopsis thaliana (6)
AtRLP1 RLP2 AtRLP3 AtRLP4 AtRLP5 AtRLP6 AtRLP7 RLP9 CLV2 AtRLP11 RLP12 AtRLP13 AtRLP14 AtRLP15 AtRLP16 TMM AT2G15042 AtRLP19 AtRLP20 AtRLP21 AtRLP22 AtRLP23 AtRLP24 AtRLP25 AtRLP27, ... AtRLP28 AtRLP29 AtRLP30 AtRLP31 AtRLP32 AtRLP33 AtRLP53, ... AtRLP35 AtRLP36 AtRLP37, ... AtRLP39 ATRLP40 AtRLP41 AtRLP42 AtRLP43 AtRLP44 AtRLP45 AtRLP46 AtRLP47 AtRLP48 AtRLP50 AtRLP52 AtRLP54 AtRLP55 ATRLP56 ATRLP57
| 2 | 0.01
Pi-score Log2-ratio Fold-Change p-value
Hormone
Stress
created with GENEVESTIGATOR

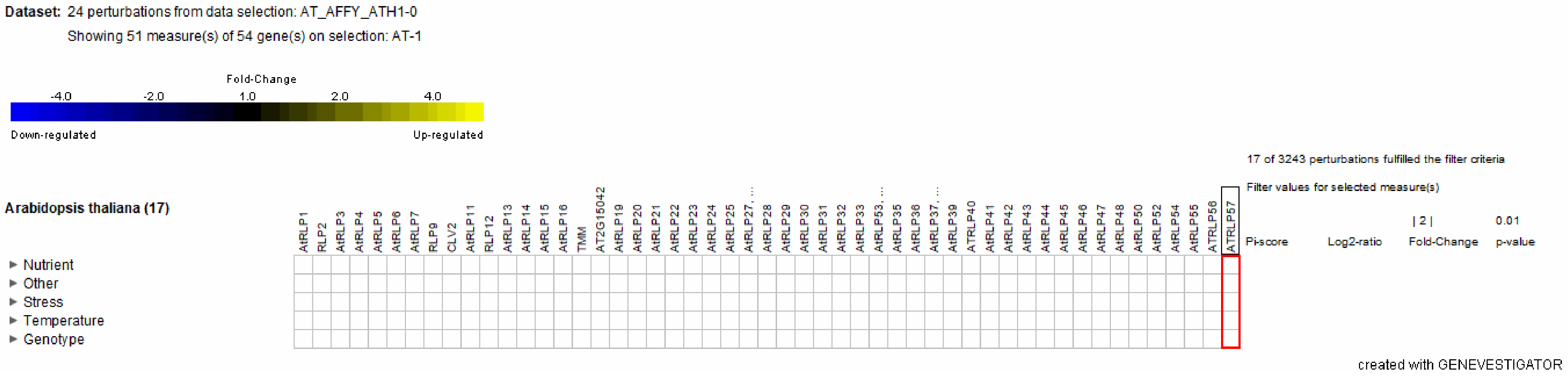
Dataset: 24 perturbations from data selection: AT_AFFY_ATH1-0
Showing 51 measure(s) of 54 gene(s) on selection: AT-1
Fold-Change
-4.0
-2.0
1.0
2.0
4.0
Down-regulated
Up-regulated
17 of 3243 perturbations fulfilled the filter criteria
Filter values for selected measure(s)
Arabidopsis thaliana (17)
AtRLP1
RLP2
AtRLP3
AtRLP4
AtRLP5
AtRLP6
AtRLP7
RLP9
CLV2
AtRLP11
RLP12
AtRLP13
AtRLP14
AtRLP15
AtRLP16
TMM
AT2G15042
AtRLP19
AtRLP20
AtRLP21
AtRLP22
AtRLP23
AtRLP24
AtRLP25
AtRLP27, ...
AtRLP28
AtRLP29
AtRLP30
AtRLP31
AtRLP32
AtRLP33
AtRLP53, ...
AtRLP35
AtRLP36
AtRLP37, ...
AtRLP39
ATRLP40
AtRLP41
AtRLP42
AtRLP43
AtRLP44
AtRLP45
AtRLP46
AtRLP47
AtRLP48
AtRLP50
AtRLP52
AtRLP54
AtRLP55
ATRLP56
ATRLP57
Pi-score
Log2-ratio
| 2 |
Fold-Change
0.01
p-value
Nutrient
Other
Stress
Temperature
Genotype
created with GENEVESTIGATOR

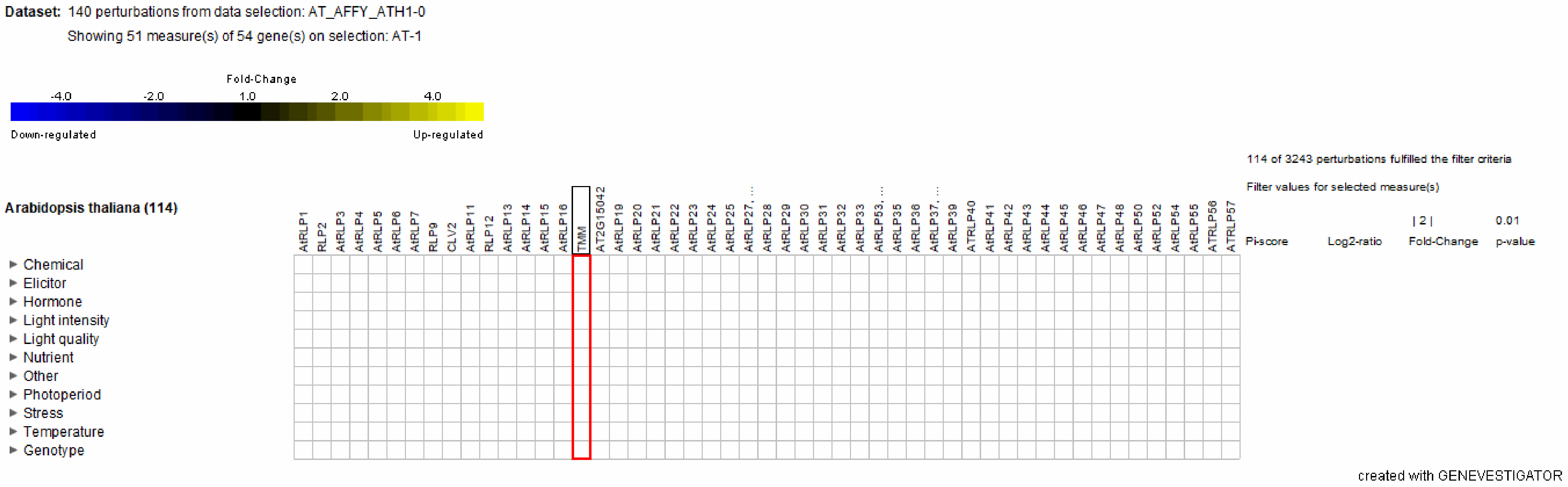

Dataset: 140 perturbations from data selection: AT_AFFY_ATH1-0
Showing 51 measure(s) of 54 gene(s) on selection: AT-1
Fold-Change
-4.0
-2.0
1.0
2.0
4.0
Down-regulated
Up-regulated
114 of 3243 perturbations fulfilled the filter criteria
Filter values for selected measure(s)
Arabidopsis thaliana (114)
AtRLP1
RLP2
AtRLP3
AtRLP4
AtRLP5
AtRLP6
AtRLP7
RLP9
CLV2
AtRLP11
RLP12
AtRLP13
AtRLP14
AtRLP15
AtRLP16
TMM
AT2G15042
AtRLP19
AtRLP20
AtRLP21
AtRLP22
AtRLP23
AtRLP24
AtRLP25
AtRLP27, ...
AtRLP28
AtRLP29
AtRLP30
AtRLP31
AtRLP32
AtRLP33
AtRLP53, ...
AtRLP35
AtRLP36
AtRLP37, ...
AtRLP39
ATRLP40
AtRLP41
AtRLP42
AtRLP43
AtRLP44
AtRLP45
AtRLP46
AtRLP47
AtRLP48
AtRLP50
AtRLP52
AtRLP54
AtRLP55
ATRLP56
ATRLP57
| 2 |
0.01
Pi-score
Log2-ratio
Fold-Change
p-value
Chemical
Elicitor
Hormone
Light intensity
Light quality
Nutrient
Other
Photoperiod
Stress
Temperature
Genotype
created with GENEVESTIGATOR
